# CD22 TCR-engineered T cells exert antileukemia cytotoxicity without causing inflammatory responses

**DOI:** 10.1126/sciadv.adq4297

**Published:** 2025-04-09

**Authors:** Kilyna A. Nguyen, Zhihui Liu, John S. Davies, Crystal P. McIntosh, Lindsey M. Draper, Scott M. Norberg, Zachary Rae, Sooraj R. Achar, Gregoire Altan-Bonnet, Ling Zhang, Xiaolin Wu, Thomas J. Meyer, Michael C. Kelly, Naomi Taylor, Christian S. Hinrichs, Kazusa Ishii

**Affiliations:** ^1^ Center for Immuno-Oncology, Center for Cancer Research (CCR), National Cancer Institute (NCI), National Institutes of Health (NIH), Bethesda, MD, USA.; ^2^ Pediatric Oncology Branch, CCR, NCI, NIH, Bethesda, MD, USA.; ^3^ Department of Safety Assessment, Genentech Inc., South San Francisco, CA, USA.; ^4^ Single Cell Analysis Facility, CCR, NCI, NIH, Bethesda, MD, USA.; ^5^ Laboratory of Integrative Cancer Immunology, CCR, NCI, NIH, Bethesda, MD, USA.; ^6^ Cancer Research Technology Program, Frederick National Laboratory for Cancer Research, NCI, NIH, Frederick, MD, USA.; ^7^ CCR Collaborative Bioinformatics Resource, CCR, NCI, NIH, Bethesda, MD, USA.; ^8^ Duncan and Nancy MacMillan Center of Excellence in Cancer Immunotherapy and Metabolism, Rutgers Cancer Institute of New Jersey, New Brunswick, NJ, USA.

## Abstract

Chimeric antigen receptor (CAR) T cells effectively treat B cell malignancies. However, CAR-T cells cause inflammatory toxicities such as cytokine release syndrome (CRS), which is in contrast to T cell receptor (TCR)–engineered T cells against various antigens that historically have rarely been associated with CRS. To study whether and how differences in receptor types affect the propensity for eliciting inflammatory responses in a model system wherein TCR and CAR target equalized sources of clinically relevant antigen, we discovered a CD22-specific TCR and compared it to CD22 CAR. Both CD22 TCR-T and CD22 CAR-T cells eradicated leukemia in xenografts, but only CD22 CAR-T cells induced dose-dependent systemic inflammation. Compared to TCR-T cells, CAR-T cells disproportionately upregulated inflammatory pathways without concordant augmentation in pathways involved in direct cytotoxicity upon antigen engagement. These differences in antileukemia responses comparing TCR-T and CAR-T cells highlight the potential opportunity to improve therapeutic safety by using TCRs.

## Teaser

Antileukemia cytotoxicity can be separated from dose-dependent inflammatory toxicity by using TCR-T as opposed to CAR-T cells.

## INTRODUCTION

Chimeric antigen receptor T cells (CAR-T) are effective against B cell malignancies (*1*–*11*). However, treatment of B cell malignancies with CAR-T is associated with cytokine-driven inflammatory toxicities, such as cytokine release syndrome (CRS) and immune effector cell-associated neurotoxicity syndrome (*12*, *13*). Systemic inflammatory response is a toxicity profile shared across different CAR-T products targeting diverse B cell antigens (*11*, *14*–*18*) and is dose limiting because of the associated morbidity and even mortality.

Historically, before CAR-T therapies were widely introduced into clinical practice, cytokine-driven inflammatory toxicities in the context of adoptive cell therapies (ACTs) were uncommon. Cell dose–limiting severe inflammatory responses rarely occur with other forms of ACT including infusions of ex vivo–expanded polyclonal antigen-specific T cells (*19*–*22*), tumor-infiltrating lymphocytes (TILs) (*23*–*27*), and T cell receptor (TCR)–engineered T cells (TCR-T) (*28*–*32*) even when coadministered with systemic interleukin-2 (IL-2). Consequently, clinically safe and recommended doses of TILs and TCR-T are generally more than 1000- to 10,000-fold higher than the doses of CAR-T (*23*–*33*). However, it is challenging to compare heterogeneous cell products containing varying frequencies of antigen-specific cells against various antigens, often used in divergent clinical contexts. Differences in toxicity profile cannot be simply attributed to CAR versus TCR dichotomy.

Preclinical comparisons of CARs to TCRs have consistently shown that the sensitivity of CARs for antigen is substantially lower compared to that of TCRs (*34*–*37*). In models of CAR constructs derived from an antibody against the peptide–major histocompatibility complex (pMHC) (as opposed to cell-surface proteins), CARs required a higher amount of cell-surface pMHCs to trigger T cell effector functions compared to TCRs against the identical pMHCs (*35*, *36*). Similarly, in other studies comparing CARs and TCRs targeting different antigens, CAR-T were less sensitive to the cognate antigens, which was attributed to inefficient signaling downstream of CAR compared to TCR (*34*, *38*). Indeed, limited reactivity of CAR-T against low-antigen leukemia cells results in disease relapses (*39*). It remains unknown how these differences in signaling and effector functions comparing CAR-T to TCR-T would affect clinical outcomes, including a propensity for causing inflammatory toxicities, if each receptor-expressing T cell was used in the same clinical context.

Here, we discovered an HLA-A*02:01–restricted TCR targeting CD22, a B cell antigen expressed by various B cell malignancies, and compared T cells transduced to express the TCR (CD22 TCR-T) with the CAR-T against the same antigen (CD22 CAR-T). The CD22 CAR-T is clinically successful but is associated with dose-limiting inflammatory toxicities revealed during dose-escalation trials (*10*, *17*). T cells activated through the TCR and CAR by the identical leukemia cell line exhibited differential transcriptional responses characterized by disproportionate and significant upregulation of inflammatory pathways in CAR-T compared to TCR-T. Despite the inflammatory transcriptional responses and high secretion of various cytokines by CAR-T compared to TCR-T, CAR-T exhibited inferior antileukemia in vitro cytotoxicity against low-antigen tumors. Consequently, the CD22 CAR-T given at the dose necessary to control leukemia in xenograft models led to systemic inflammatory responses, while the CD22 TCR-T cleared leukemia without inducing systemic cytokine elevation. These results suggest that cell dose–dependent inflammatory responses can be separated from antileukemia efficacy by using TCR-activated T cells as opposed to CAR-T, providing the insights into how to control inflammatory toxicities of adoptive T cell therapies.

## RESULTS

### A TCR specific to an HLA-A*02:01–restricted epitope of CD22 was isolated from allogeneic TCR repertoire

**Fig. 1.**
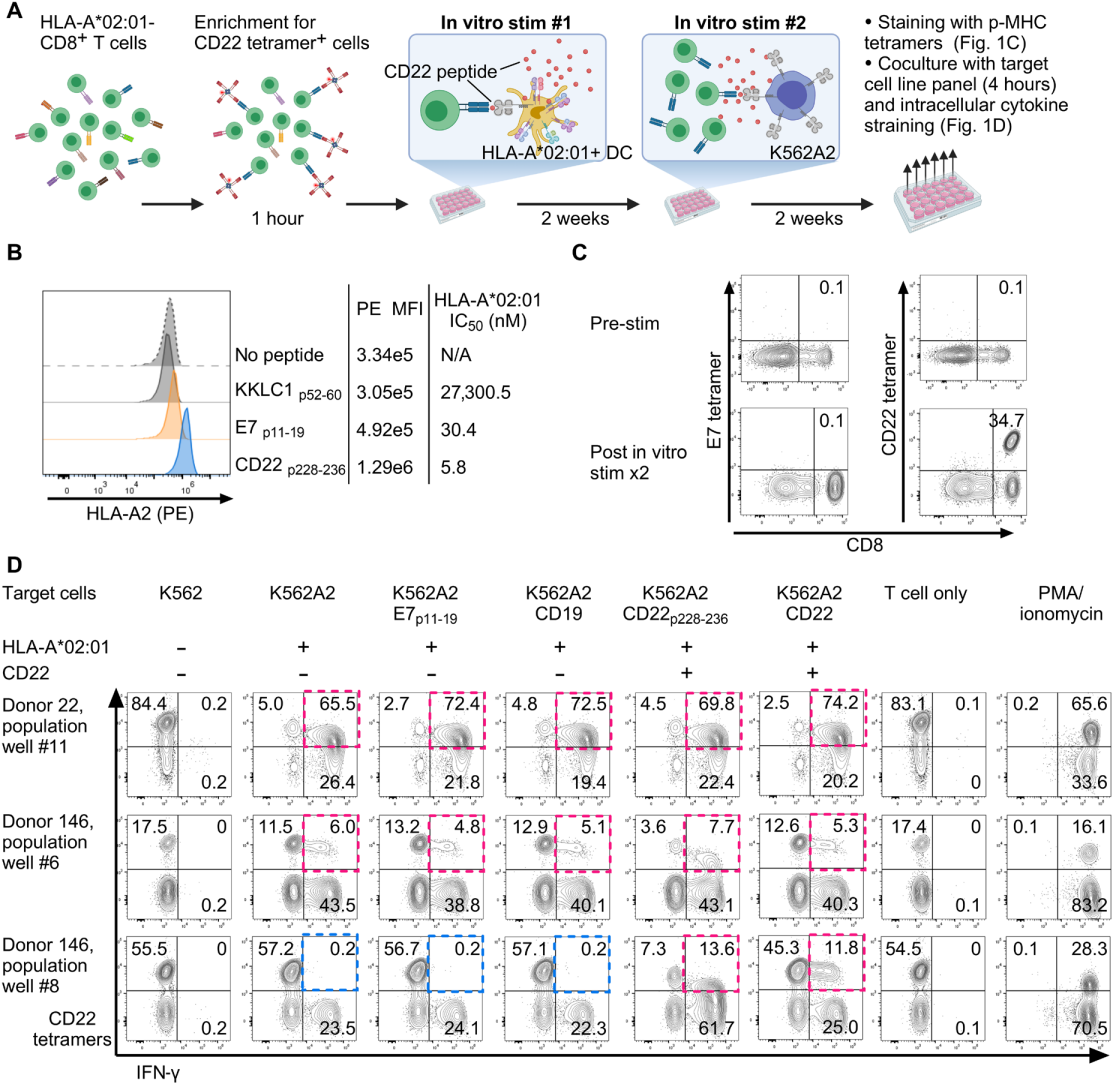
Identification of HLA-A*02:01–restricted TCRs against CD22 from HLA-A*02:01–negative donors. (**A**) HLA-A*02:01^−^ CD8^+^ T cells were isolated from PBMC by magnetic negative selection. CD8^+^ T cells were incubated with CD22 peptide–HLA-A*02:01 tetramers for 1 hour, and the tetramer-bound population was positively enriched. Tetramer-bound CD8^+^ T cells were stimulated with HLA-A*02:01^+^ DC loaded with the CD22_p228–236_ peptide. Second stimulation was provided with irradiated K562A2 (K562 expressing only HLA-A*02:01 allele) in the presence of irradiated PBMC autologous to the CD8^+^ T cells. Illustrations were made with BioRender.com. (**B**) HLA-A2 peptide-binding assay (Supplementary Methods). Cell-surface HLA-A2 expression on a TAP-deficient cell line (T2) was assessed by flow cytometry after overnight incubation with indicated peptides in serum-free media. HPV-16 E7_p11–19_: HLA-A*02:01–restricted (*42*). KKLC1_p52–60_: HLA-A*01:01–restricted (*108*). (**C**) Cells from the in vitro stimulation wells were assessed for binding to CD22 tetramers and E7 tetramers by flow cytometry. Representative dot plots are shown. (**D**) Cells in in vitro stimulation plate were cocultured for 4 hours with the indicated cell lines (E:T = 1:1, 1 × 10^5^ cells each), and tetramer binding and IFN-γ production were assessed by flow cytometry. Representative dot plots of donor 22-well #11 and donor 146-wells #6 and #8 are shown. Among the CD22 tetramer^+^ population, the presence and absence of IFN-γ responses are highlighted in pink and blue, respectively.

CD22 is a nonmutated self-antigen expressed by B cells. Given physiological thymic selection processes, high-affinity TCRs against self-antigen–derived peptides are expected to be absent from a healthy individual’s TCR repertoire. To circumvent the issue of central tolerance, we used an approach of allogeneic stimulation to enrich T cells expressing TCRs specific to an HLA-A*02:01–restricted epitope of CD22 (CD22_p228–236_, amino acid FLSNDTVQL) (Fig. 1A). CD22_p228–236_ is a peptide estimated to bind to HLA-A*02:01 at high affinity based on a prediction algorithm (IEDB, www.iedb.org) (*40*) and reported to be a naturally processed and presented epitope of B cells (*41*). Consistent with these reports, CD22_p228–236_ peptide stabilized and augmented cell-surface expression of HLA-A*02:01 complexes in a transporter associated with antigen processing (TAP)-deficient cell line (Fig. 1B), confirming that the peptide binds to HLA-A*02:01 and is likely able to form a stable p-MHC tetramer. To increase the precursor frequencies of TCRs that recognize the CD22_p228–236_ peptide–HLA-A*02:01 complex, CD8^+^ T cells from an HLA-A*02:01^−^ donor were first pre-enriched for the population bound to CD22_p228–236_ peptide–HLA-A*02:01 tetramers (CD22 tetramers) using magnetic positive isolation. Then, monocyte-derived dendritic cells (DCs) were generated from an HLA-A*02:01^+^ healthy donor. CD8^+^ CD22 tetramer^+^ enriched T cells were stimulated with CD22 peptide–loaded DCs. Two weeks after the initial stimulation, CD8^+^ T cells were restimulated using irradiated K562 cells engineered to express HLA-A*02:01 as artificial antigen-presenting cells (APCs). K562 lacks cell-surface expression of endogenous HLA molecules. HLA-A*02:01 mono-allele-transduced K562 was used with an assumption that it would preferentially expand T cells expressing HLA-A*02:01–restricted TCRs while mitigating allogeneic expansion of T cells unrelated to the HLA-A*02:01 restriction element. This method efficiently expanded the CD8^+^ population that bound to CD22 tetramers but not to irrelevant tetramers such as HPV-16 E7_p11–19_ HLA-A*02:01 tetramers (E7 tetramers) (Fig. 1C and fig. S1). To further assess the specificity of the TCRs expressed by the CD22 tetramer–binding T cell population, polyclonal cells in each well of in vitro stimulation plate were cocultured with a panel of cell lines, and interferon-γ (IFN-γ) responses were assessed with intracellular cytokine staining (Fig. 1D and fig. S2). In donor 22 (Fig. 1D, top), there were CD22 tetramer–binding CD8^+^ T cells, but tetramer-bound cells produced IFN-γ in response to CD22-negative HLA-A*02:01^+^ cells, indicating their reactivity to ligands unrelated to CD22. Similarly, in donor 146 in vitro stimulation well #6 (middle), CD22 tetramer–binding population had IFN-γ responses unrestricted by target cells’ CD22 expression status. Conversely, in a separate well #8 from the same donor 146, CD22 tetramer–binding population produced IFN-γ only when the target cells expressed both HLA-A*02:01 and CD22, suggesting that this T cell population expressed TCRs of the desired specificity.

### CD22 TCR-transduced T cells recognize targets in a manner restricted by both HLA-A*02:01 and CD22

**Fig. 2.**
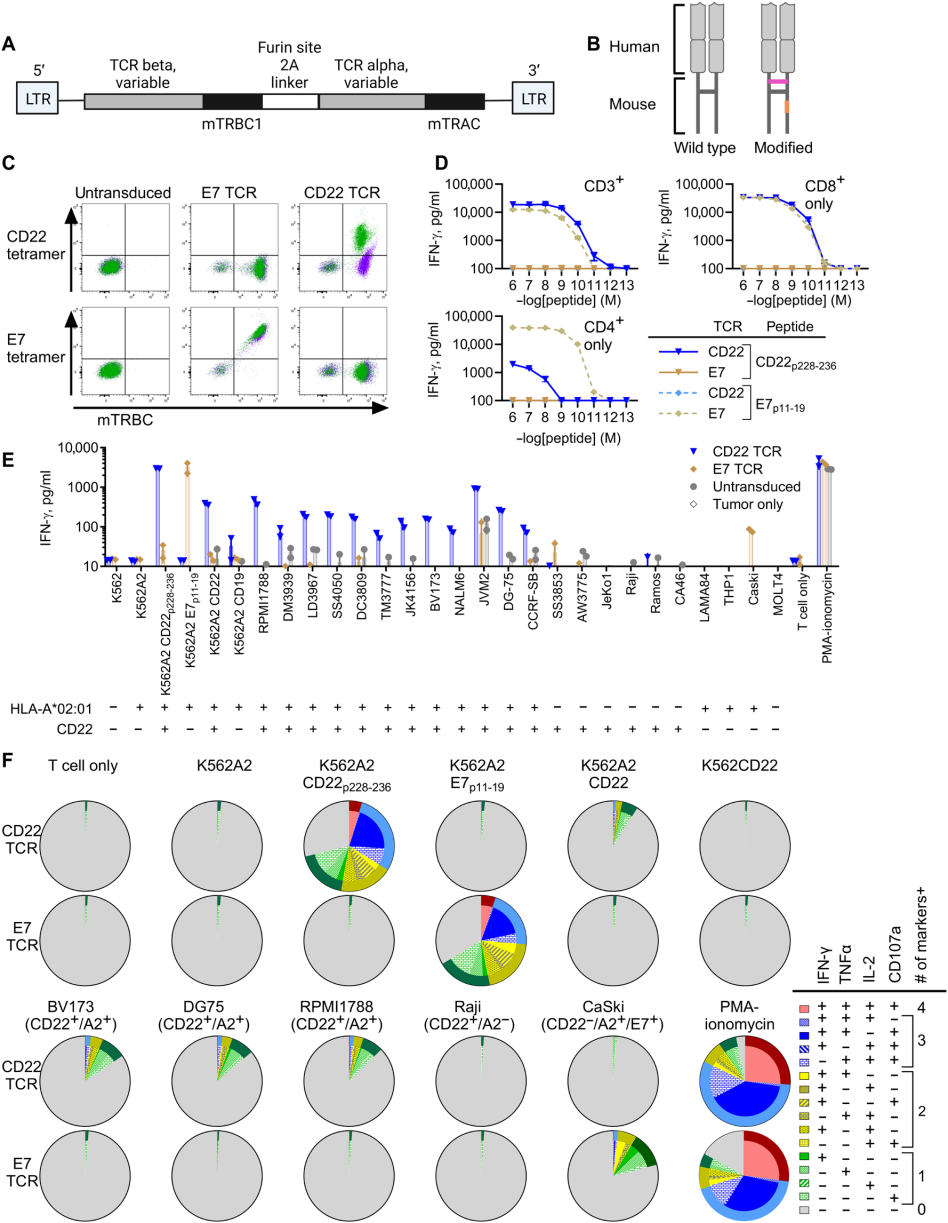
The CD22 TCR recognizes B cell leukemia and lymphoma cells in an HLA-A*02:01– and CD22-dependent manner. (**A**) Schematic of the gammaretroviral vector encoding the CD22 TCR alpha and beta chains. (**B**) Design of the TCR composed of human variable regions and mouse constant regions with modifications as previously described (*42*–*44*) (Supplementary Methods). (**C**) CD22 TCR– and E7 TCR–transduced T cells were stained with CD22 or E7 tetramers: green (CD8^+^) and purple (CD4^+^). Murine TCR beta constant region (mTRBC) is a marker of transduction efficiency. (**D**) Bulk (CD3^+^), CD8^+^, and CD4^+^ enriched T cells transduced to express either the CD22 TCR or E7 TCR were cocultured with K562A2 loaded with the indicated peptides (E:T = 1:1). IFN-γ levels in the overnight coculture were measured by ELISA. (**E**) CD22 TCR-T and E7 TCR-T (bulk, CD3^+^) were cocultured overnight with the indicated cell lines (E:T = 1:1). IFN-γ levels were measured by ELISA. The CD22 and HLA-A*02:01 expression of each cell line is indicated as + or –. (**F**) CD22 TCR-T and E7 TCR-T were cocultured with the indicated cell lines for 4 hours (E:T = 2:1). CD107a and intracellular IFN-γ, TNFα, and IL-2 were assessed by flow cytometry. Pie charts show the percentages of TCR-T expressing each combination of markers. Representative figure from three independent experiments, each performed with two biological replicates (independent PBMC donors). Technical replicates: *n* = 3 (D) and *n* = 2 [(E) and (F)]. Illustrations [(A) and (B)] were made with BioRender.com.

CD22 tetramer–binding cell population in the donor 146 well #8 was magnetically isolated and single-cell TCR α/β paired sequencing was performed. There was only one predominant clonotype comprising 96% of productive V(D)J pairs sequenced (table S1). The TCR was reconstructed and cloned into a gamma-retroviral vector, pMSGV1 (CD22 TCR hereafter). TCR constant regions were substituted with murine counterparts with modifications known to facilitate correct TCR α/β pairing and improve cell-surface TCR expression as described previously (*42*–*44*) (Fig. 2, A and B). Human T cells transduced with the CD22 TCR (CD22 TCR-T) bound to CD22 tetramers but not the unintended HPV-16 E7 tetramers (Fig. 2C). The CD22 TCR-T had high functional avidity, recognizing the cognate peptide at concentrations as low as 10 pM, albeit dependent on CD8 coreceptors (Fig. 2D and fig. S3A). In addition to the exogenously added epitope peptide, CD22 TCR-T were able to recognize and polyfunctionally respond to multiple leukemia and lymphoma cell lines naturally expressing HLA-A*02:01 and full-length CD22 protein (Fig. 2, E and F). Functional responses of CD22 TCR-T were next compared to those of the TCR-T specific to an HLA-A*02:01–restricted viral epitope of EBV LMP2 (LMP2_p426–434_) (*45*). Despite the fact that EBV LMP2 TCR-T have exquisitely high functional avidity with an ability to recognize the epitope independently of CD8 coreceptor engagement (fig. S3A), the CD22 TCR-T demonstrated stronger IFN-γ responses and in vitro cytotoxicity against HLA-A*02:01^+^ EBV-transformed B cell lymphoblastoid cells and EBV^+^ lymphoma/leukemia cells that naturally express both CD22 and EBV LMP2 antigens (fig. S3, B and C). This observation suggests that naturally processed and presented target epitopes derived from CD22, compared to EBV LMP2, are likely more abundant in these target cells. Collectively, the data show that the CD22 TCR redirects human polyclonal T cells to an HLA-A*02:01–restricted CD22 epitope that is naturally processed and presented by malignant cells.

### CD22 TCR does not cross-react with unintended peptides from human proteome at physiological concentrations

**Fig. 3.**
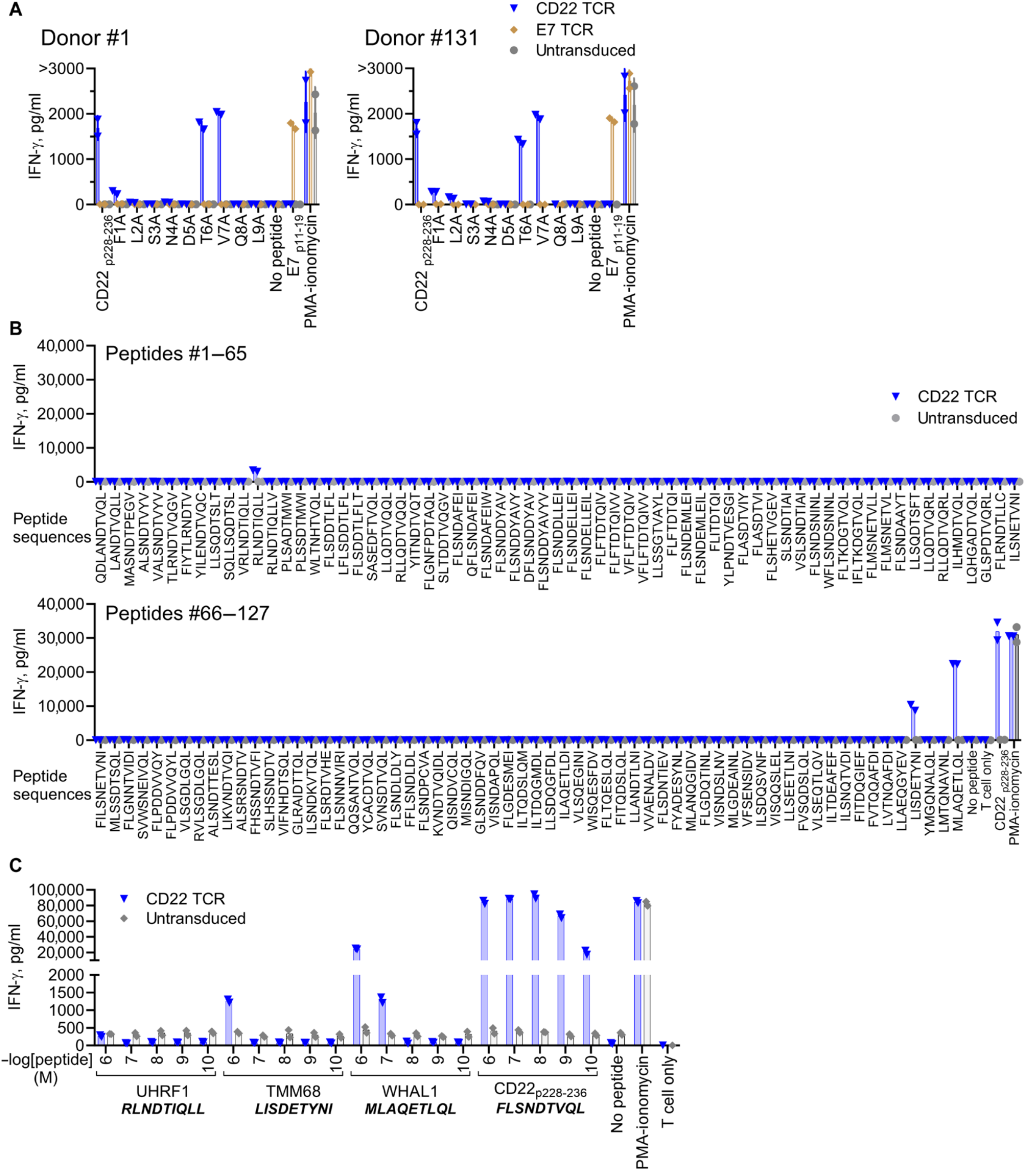
The CD22 TCR does not cross-react with other peptides from human proteome at physiological concentrations. (**A**) TCR-transduced T cells were cocultured with K562A2 loaded with 1 μM of the CD22 epitope peptide (CD22_p228–236_) or altered-ligand peptides with alanine substitution of each residue. IFN-γ levels in the overnight coculture supernatant were measured by ELISA. (**B**) CD22 TCR-T were cocultured with K562A2 loaded with cross-reactive screening candidate peptides #1 to 127 (1 μM). IFN-γ levels in the overnight coculture supernatant were measured by ELISA. (**C**) CD22 TCR-T were cocultured with K562A2 loaded with the indicated concentrations of peptides. IFN-γ levels in the overnight coculture supernatant were measured by ELISA. Coculture assays were set up at an E:T = 1:1. Representative figures from three independent experiments, each performed with two biological replicates (independent PBMC donors). Technical replicates: *n* = 2.

Given that the CD22 TCR was isolated from an HLA-A*02:01^−^ allogeneic repertoire, the risk of cross-reactivity with unintended self-antigens may be higher than the risk associated with TCRs isolated from HLA-A*02:01^+^ individuals. To determine the residues critical for contacting the TCR, CD22 TCR-T were stimulated with the CD22 epitope peptide and the altered peptide ligands with alanine or glycine substitution of each residue (Fig. 3A and fig. S4A). Substitutions of the second and the ninth residues were estimated to reduce the peptide affinity for HLA-A*02:01 (table S2), indicating that these residues are involved in HLA-A*02:01 anchoring. Substitution of nonanchor residues 3, 4, 5, and 8 almost completely abrogated IFN-γ production by T cells, suggesting that these are nonanchor residues critical for TCR contact. On the basis of this motif information, in silico search was carried out to identify peptides sharing the contact motif sequence from human proteome (Supplementary Methods, “Search 1” of fig. S4B). Because cross-reactive peptides may not necessarily share the TCR recognition motif sequences, in silico search rule was expanded to allow substitution of each residue with amino acids with physicochemical similarity as previously described (*45*) (“Search 2” of fig. S4B). In addition, in silico search using protein BLAST (blast.ncbi.nlm.nih.gov) was performed (“Search 3” of fig. S4B). All these searches combined, a total of 170 unique peptides were selected as cross-reactivity screening candidates. Of 170 candidates, three peptides—UHRF1, TMM68, and WHAL1-derived peptides—were recognized by CD22 TCR-T at a supraphysiologically (*46*) high concentration of 1 μM (Fig. 3B and fig. S4C). However, these three peptides did not elicit IFN-γ responses from CD22 TCR-T at physiologically relevant (*46*) concentrations (Fig. 3C). Together, the data demonstrate that the CD22 TCR-T are unlikely to cross-react with other human proteome-derived peptides in physiological contexts.

### CD22 TCR-T mediate antileukemia and lymphoma activity

**Fig. 4.**
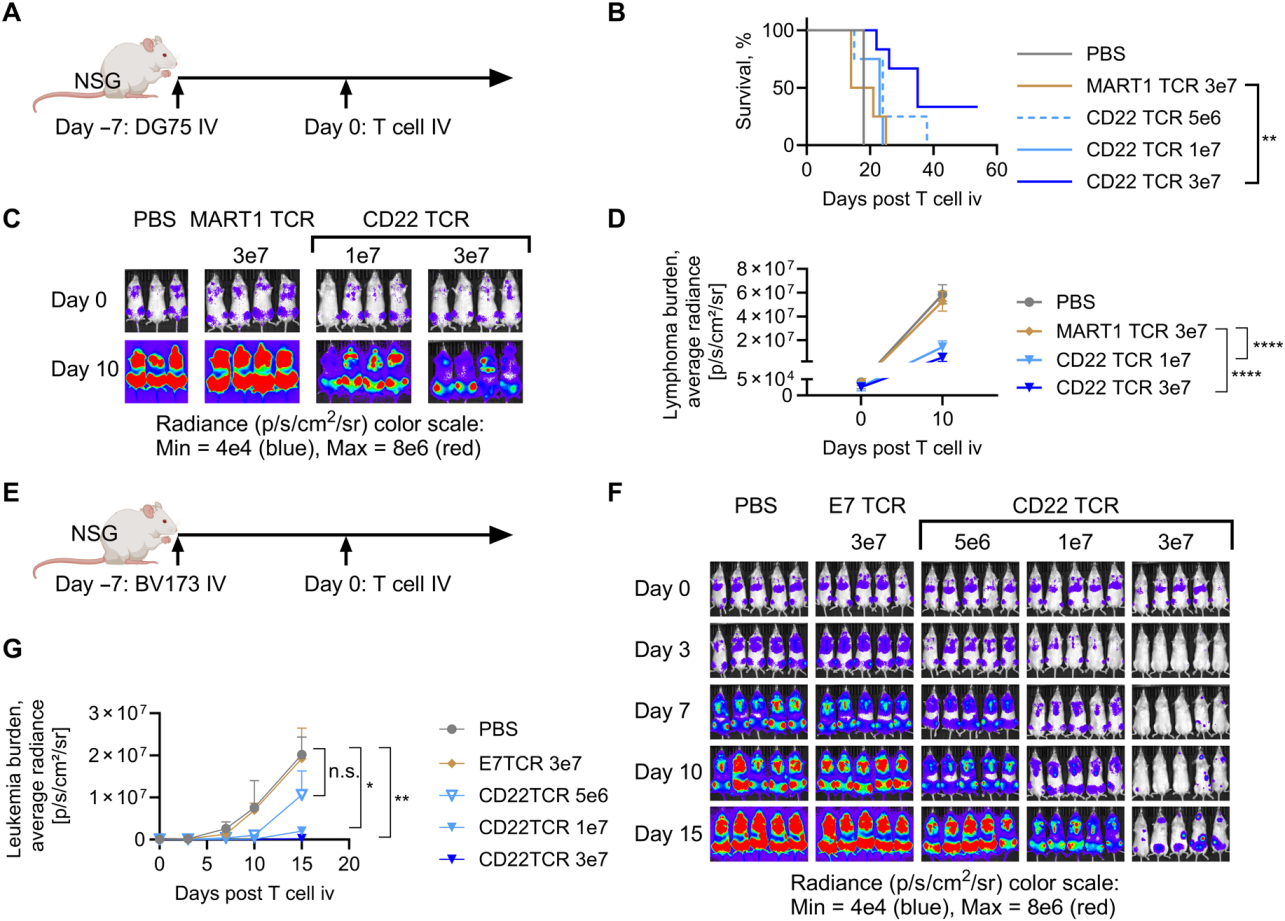
The CD22 TCR-T mediates in vivo antileukemia/lymphoma activity. (**A**) NSG mice were intravenously injected with human lymphoma cell line DG-75 expressing firefly luciferase (DG-75-ffLuc) 1 × 10^6^ cells per mouse on day −7, and T cells were intravenously administered on day 0 at doses described in subsequent figure legends. (**B**) Survival of treated mice are shown. MART1 TCR (clone DMF5) is specific to an HLA-A*02:01–restricted epitope MART-1_p26–35_, an antigen not expressed by the target cell line. (**C** and **D**) Bioluminescent signals (tumor burden) were measured by IVIS (Perkin Elmer). Color scale is shown in fig. S21. (C) IVIS images and (D) average radiance of bioluminescent signals are shown. (**E**) NSG mice were intravenously injected with human leukemia cell line BV173 expressing firefly luciferase (BV173-ffLuc) 1 × 10^6^ cells per mouse on day −7, and T cells were intravenously administered on day 0. (**F** and **G**) Bioluminescent signals were measured by IVIS, and images (F) and average radiance (G) are shown. Biological replicates: *n* = 4 (*n* = 5 in CD22 TCR 3 × 10^7^ group) (B), *n* = 4 (*n* = 3 in PBS group) [(C) and (D)], and *n* = 5 [(F) and (G)]. Representative figure from three independent experiments. *P < 0.05, **P < 0.01, and ****P < 0.0001 by log-rank Mantel-Cox method (B), one-way ANOVA with Holm-Sidak correction (D), and Kruskal-Wallis test with Dunn’s correction (G). n.s., not significant. Illustrations [mouse in (A) and (E)] were made with BioRender.com.

CD22 TCR-T were tested in NSG xenograft models. CD22 TCR-T exerted in vivo cytotoxicity against Burkitt lymphoma cell line (DG-75) (Fig. 4, A to D). Similarly, in a model of CML blast crisis (BV173) (Fig. 4E), systemic leukemia burden was significantly reduced in groups of mice treated with CD22 TCR-T in a cell dose–dependent manner (Fig. 4, F and G). CD22 TCR-T (and E7 TCR-T) were detectable beyond 3 to 4 weeks after T cell infusion depending on the administered cell doses (fig. S6, A and B), although the interpretation of in vivo T cell data at a later time point in xenograft models is generally limited by the well-known confounding effects of xenogeneic reactions (fig. S5) (*47*). Leukemia cells that have lost both CD22 and HLA-A2 expression (fig. S6, C to E) emerged in groups of mice that received CD22 TCR-T at suboptimal dosages.

**Fig. 5.**
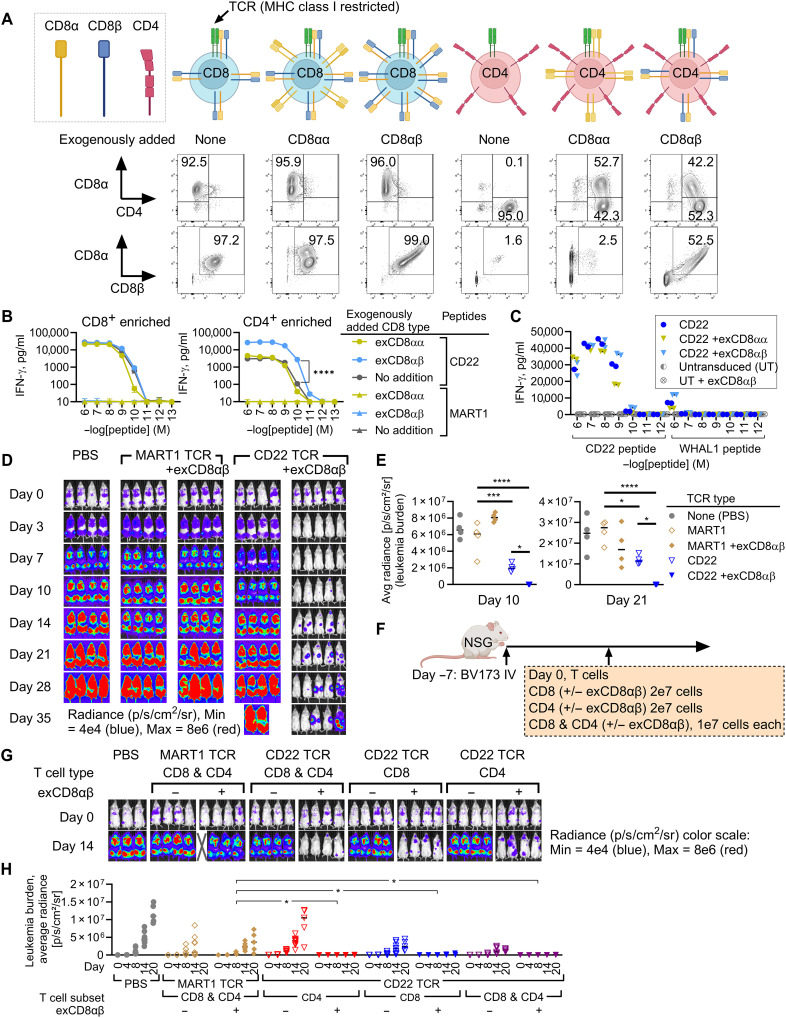
Exogenous addition of CD8αβ heterodimers augments antileukemia activity of the CD22 TCR-T. (**A**) CD8^+^ or CD4^+^ T cells were co-transduced with TCR and either CD8α or CD8α + CD8β chains. Schematic illustration and representative flow cytometry dot plots show the cell-surface coreceptor expression patterns. (**B**) CD22 TCR-T were cocultured overnight with peptide-loaded K562A2, and IFN-γ levels were measured (ELISA). Exogenously added CD8αα and CD8αβ; exCD8αα and exCD8αβ, respectively. (**C**) CD8^+^ CD22 TCR-T with or without exCD8αα or exCD8αβ were cocultured with peptide-loaded K562A2. WHAL1 peptide is weakly cross-reactive with the CD22 TCR (Fig. 3). (**D** and **E**) Using the experimental schema in Fig. 4E, mice were treated with bulk T cells (CD3^+^) expressing either CD22 TCR or MART1 TCR with or without exCD8αβ (1 × 10^7^ cells per mouse). Bioluminescent signals measured by IVIS are shown as images (D) and average radiance (E). (**F** to **H**) Using the experimental schema in Fig. 4E, mice were treated with CD8^+^, CD4^+^, or 1:1 mixture of CD8^+^ and CD4^+^ T cells transduced with CD22 TCR or MART1 TCR, each with and without exCD8αβ. IVIS images (G) and average radiance (H) are shown. [(B to (E), (G), and (H)] Representative of three independent experiments. Technical replicates: *n* = 3 (B) and *n* = 2 (C). Biological replicates: *n* = 4 to 5 [(D) to (H)] (except PBS in G; *n* = 3). *P < 0.05, ***P < 0.001, and ****P < 0.0001, by Kruskal-Wallis test with Dunn’s correction [(B), (E), and (H)]. Illustrations [(A), mouse in (F)] were made with BioRender.com.

Because the CD22 TCR-T recognized targets in a CD8 coreceptor–dependent manner (Fig. 2, C and D, and fig. S3A), we next assessed whether antimalignancy activities of CD22 TCR-T can be improved by exogenously adding CD8 coreceptor. There are two types of CD8 chains, CD8α and CD8β, which form CD8αα homodimer and CD8αβ heterodimer (*48*). To make TCR-T products with transgenic expression of CD8αα homodimer or CD8αβ heterodimer, CD22 TCR-T were co-transduced with either a CD8α vector or a bicistronic vector encoding both CD8α and CD8β (Fig. 5A and Supplementary Methods). Cognate CD22 tetramer binding of CD22 TCR-T was augmented in the presence of exogenously added CD8αβ heterodimers (exCD8αβ) without increasing nonspecific binding to irrelevant tetramers (fig. S7). Functional avidity of CD4^+^ CD22 TCR-T measured by IFN-γ responses improved with exCD8αβ. On the other hand, coexpression of exogenously added CD8αα (exCD8αα) increased nonspecific tetramer binding (fig. S7) without improving IFN-γ responses (Fig. 5B), which may be an observation consistent with the reports that CD8αα functions as an adhesion molecule (*49*–*51*). The functional avidity of CD22 TCR-T against the WHAL1 peptide—a top candidate peptide identified to be weakly cross-reactive (Fig. 3C)—barely changed with either exCD8αβ or exCD8αα, suggesting that the risk of cross-reactivity of the CD22 TCR-T did not increase (Fig. 5C). To evaluate in vivo cytotoxicity of CD22 TCR-T coexpressing exCD8αβ, leukemia-bearing mice were treated with bulk CD22 TCR-T (mixture of both CD8 and CD4) with or without exCD8αβ. The CD22 TCR-T coexpressing exCD8αβ achieved better control of leukemia (Fig. 5, D and E) and lymphoma (fig. S8) compared to the TCR-T alone.

IFN-γ responses of CD4^+^ CD22 TCR-T, but not CD8^+^ CD22 TCR-T, substantially improved with exCD8αβ (Fig. 5B). To assess whether exCD8αβ differently affects T cells’ in vivo cytotoxicity depending on the endogenous coreceptor types, leukemia-engrafted mice were treated with CD8^+^, CD4^+^, or a 1:1 mixture of CD8^+^ and CD4^+^ CD22 TCR-T, each with and without exCD8αβ (Fig. 5F). Leukemia clearance by CD4^+^ CD22 TCR-T improved with exCD8αβ as expected (Fig. 5, G and H). In vivo T cell frequencies at a later time point were generally higher in CD4^+^ compared to CD8^+^ T cell products, with or without exCD8αβ (fig. S9). Notably, the antileukemia activity of CD8^+^ CD22 TCR-T also improved when CD8αβ expression was exogenously augmented, suggesting that the quantity of CD8αβ was one of the limiting factors of antileukemia effector functions exerted by the MHC class I–restricted CD22 TCR-T. Collectively, the data indicate that CD22 TCR-T mediate antileukemia and antilymphoma in vivo activity, which can be enhanced by CD8αβ heterodimer coexpression.

### Leukemia cells elicit proinflammatory responses in CAR-T but not in TCR-T

**Fig. 6.**
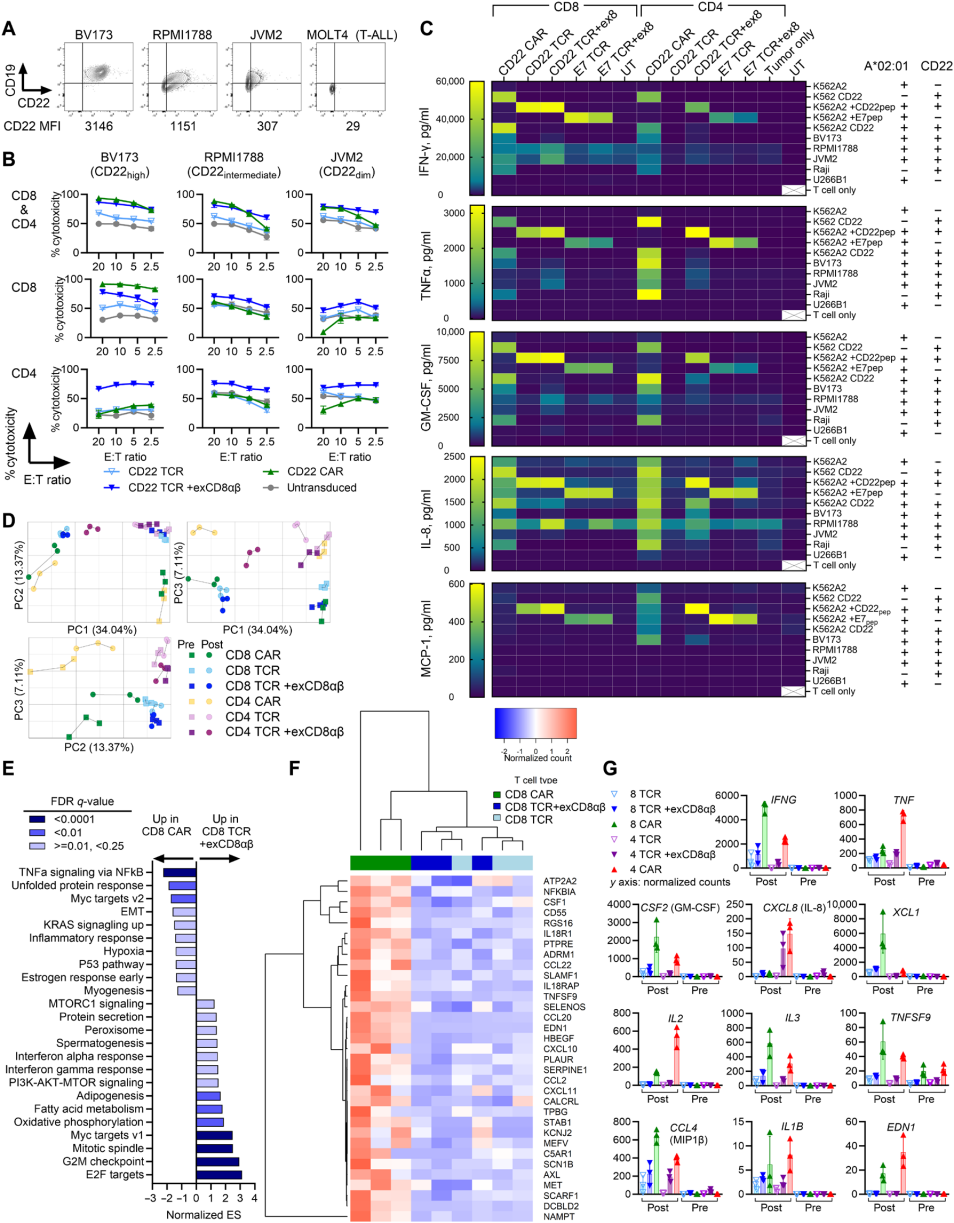
Pro-inflammatory responses are overrepresented in CAR-activated T cells compared to TCR-activated T cells. (**A**) CD22 and CD19 cell-surface expression on B cell leukemia and lymphoma lines. MOLT4 is a T-acute lymphoblastic leukemia line (negative control). Mean fluorescence intensity (MFI). (**B**) In vitro cytotoxicity of CD8^+^ and CD4^+^ CD22 CAR-T and CD22 TCR-T with or without exCD8αβ against indicated cell lines after 4 hours of coculture. (**C**) Levels of indicated cytokines in overnight coculture supernatant (E:T = 1:1) are shown (ELISA). (**D** to **G**) Transcriptional profiling of CD22 CAR-T and CD22 TCR-T with or without exCD8αβ at baseline and 6 hours following stimulation with BV173 (fig. S11). (D) The 2D plots of top three principal components (PCs). (E) GSEA of RNA-seq data for contrasts between CD8 CAR and CD8 TCR + exCD8αβ post-stimulation. Gene sets (hallmark) that were enriched in either direction with FDR *q*-value <0.25 and adjusted *P* value <0.05 are shown. (F) Unsupervised clustering analysis of RNA normalized counts of genes identified in the leading edge subset of inflammatory response gene sets. (G) RNA normalized counts of select genes are shown for CD8^+^ (*8*) and CD4^+^ (*4*) cell products pre- and post-stimulation. ExCD8αβ is abbreviated as +ex8 (C). Representative figure from three independent experiments [(B) and (C)]. Technical replicates: *n* = 3 (B) and *n* = 2 (C). Biological replicates: *n* = 3 [(D) to (G)].

We next compared the in vitro activities of the CD22 TCR-T to those of the CD22 CAR-T (m971-41BBz), the CAR construct in clinic against the same source of CD22 antigen (*10*, *17*, *52*). CD22 is expressed at varying cell-surface densities by B cell leukemia and lymphoma (Fig. 6A). Comparison of the in vitro cytotoxicity of the CD22 TCR-T and the CD22 CAR-T (Fig. 6B) showed findings consistent with prior reports describing relative insensitivity of CAR-T against low-antigen tumors (*34*–*37*): Among the group of CD8^+^ purified products, CD8^+^ CAR-T demonstrated the highest cytotoxicity against CD22-high expressors (BV173). However, CD8^+^ CAR-T could not mediate cytotoxicity above the nonspecific background level (i.e., untransduced T cell) when target cells had lower levels of cell-surface CD22 expression. Conversely, CD8^+^ TCR-T with exCD8αβ retained cytotoxicity against all cell lines. Among the CD4^+^ purified products, CD4^+^ TCR-T with exCD8αβ had the best cytotoxicity. Differential cytotoxicity comparing bulk (i.e., mixture of CD8 and CD4) CAR-T to TCR-T plus exCD8αβ was manifested as better clearance of CD22-low leukemia by TCR-T at low effector-to-target (E:T) ratios. Curiously, despite the antigen density–dependent reduction in cytotoxicity, CAR-T, particularly the CD4^+^ CAR-T that mediated dismal in vitro cytotoxicity, produced larger amounts of various cytokines compared to TCR-T regardless of target cell-surface CD22 levels (Fig. 6C and fig. S10). Of note, CD22 TCR-T with or without exCD8αβ produced equivalently large amounts of various cytokines as CD22 CAR-T when they received stimulations through high concentration (1 μM) of exogenously added epitope peptides (Fig. 6C and fig. S10), suggesting that cytokine responses of CAR-T are equivalent to TCR-T receiving supraphysiologically strong inputs.

To explore why the magnitude of cytotoxicity and cytokine responses are discordant in CD22 CAR-T compared to TCR-T, we compared the gene expression profile of CD22 CAR-T and CD22 TCR-T with and without exCD8αβ, before and 6 hours after in vitro stimulation with leukemia cells (fig. S11A). Principal component analysis of RNA sequencing (RNA-seq) data showed that the top three principal components (PCs) accounted for approximately 55% of variability (fig. S11B), visually distinguishing pre- from post-stimulation state (reflected in PC1), CAR-T from TCR-T (PC2), and CD4^+^ from CD8^+^ (PC3) (Fig. 6D). CAR-T had a transcriptional state that was distinct from TCR-T with or without exCD8αβ at both pre- and post-stimulation. Gene set enrichment analysis (GSEA) of the contrast between post-stimulation CD8^+^ CAR-T to CD8^+^ TCR-T with or without exCD8αβ showed that CAR-mediated activation of T cells resulted in significant positive enrichment of “inflammatory response (hallmark)” (Fig. 6E and fig. S12) and numerous cytokine- and chemokine-related gene sets (Fig. 6F and fig. S13). Before antigen-specific stimulation, these inflammation-related gene sets were not enriched in either direction comparing CD8^+^ CAR-T to CD8^+^ TCR-T with or without exCD8αβ (fig. S12). Among the significantly differentially expressed transcripts, cytokines implicated in the CRS pathology such as *IFNG*, *TNF*, *CSF2* [granulocyte-macrophage colony-stimulating factor (GM-CSF)], and *IL1B* were higher in CAR-T compared to TCR-T with or without exCD8αβ (Fig. 6G and fig. S14). In addition, coagulation-related gene transcripts such as *PRSS23* and *SERPINE2* and transcripts for *EDN1* (endothelin 1) that contributes to endothelial dysfunction—one of the hallmarks of severe CRS after CAR-T (*16*, *53*)—were elevated in CAR-T (Fig. 6G and fig. S14).

In contrast, T cells’ responsiveness to type I and type III interferons and the receptor downstream signaling such as “PI3K-AKT-MTOR signaling” were significantly positively enriched in post-stimulation TCR-T with or without exCD8αβ compared to CAR-T (Fig. 6E and fig. S12). Gene sets related to granule-mediated direct cytotoxicity such as “GOCC_Cytolytic_Granule (C5, GO:0044194)” and “GOBP_Leukocyte_Degranulation (C5, GO:0043299)” did not emerge as differentially enriched (fig. S14). The contrast between CD8^+^ TCR-T to CD8^+^ TCR-T with exCD8αβ showed that an addition of exCD8αβ resulted in significant positive enrichment of gene sets related to cell proliferation pre- and post-stimulation, such as “E2F targets” and “G2M checkpoint,” as well as gene sets related to T cell activation and functions including “MTORC1 signaling” and “IL-2–STAT5 signaling” (fig. S12). Importantly, the addition of exCD8αβ that augmented antileukemia cytotoxicity of TCR-T did not accompany concurrent upregulation of inflammation-related gene sets (figs. S12 and S13). Contrasts among CD4^+^ cell products revealed similar findings (fig. S15). These data collectively show that CAR-mediated activation of T cells disproportionately upregulated inflammatory transcriptional responses over other effector responses in T cells, resulting in overproduction of proinflammatory mediators compared to TCR-activated T cells.

### Cell dose–dependent systemic inflammatory responses occur with CD22 CAR-T but not with CD22 TCR-T cell therapy

**Fig. 7.**
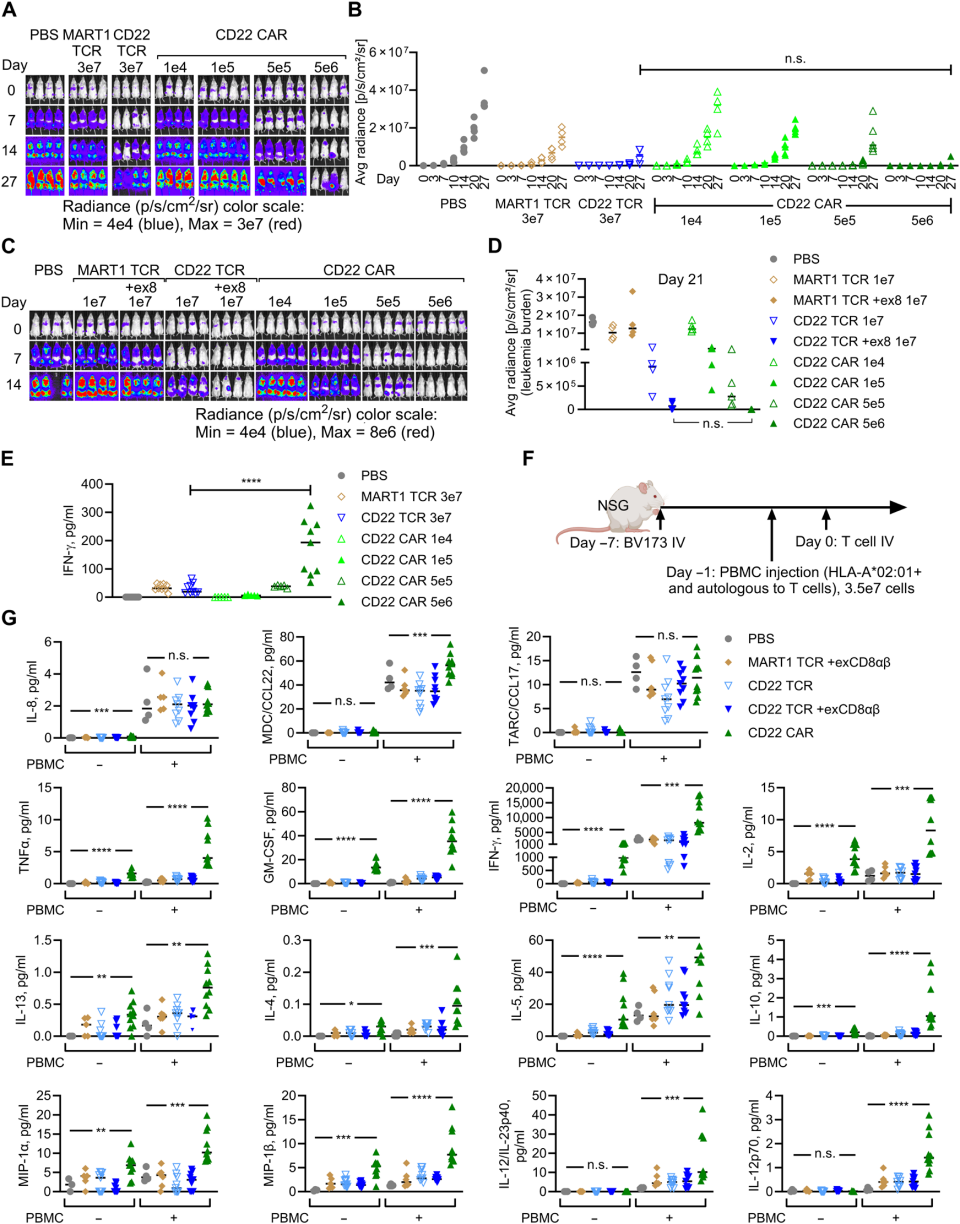
CD22 TCR-T eradicate leukemia without causing systemic inflammation, while CD22 CAR-T induce cell dose–dependent inflammation. (**A** to **E**) Using the experimental schema in Fig. 4E, mice were treated with indicated type and doses of T cells. CAR-T dose that mediates equivalent cytotoxicity as CD22 TCR-T 3 × 10^7^ [(A) and (B)] and CD22 TCR-T + exCD8αβ (+ex8) 1 × 10^7^ (C, D) were identified. Bioluminescent signals measured by IVIS are shown as images [(A) and (C)] and average radiances [(B) and (D)]. (**E**) Serum IFN-γ levels on day 2 measured by MSD assay. (**F**) Leukemia-bearing mice were injected on day −1 with bulk PBMC (3.5 × 10^7^) from the HLA-A*02:01^+^ donor autologous to the T cells. On day 0, CAR-T (5 × 10^6^) or TCR-T with or without exCD8αβ (2 × 10^7^) were injected. (**G**) Serum cytokine levels on day 2 measured with MSD assay. All experiments in this figure are with bulk T cells. Representative from three (A to E) and two (G) independent experiments. Biological replicates: [(A) and (B)] *n* = 5 (CAR 1 × 10^5^ and CAR 5 × 10^5^), *n* = 4 (all other); [(C) and (D)] *n* = 5 (CAR groups), *n* = 4 (all other). (E) *n* = 5 (CAR 1 × 10^4^, CAR 1 × 10^5^, CAR 5 × 10^5^, and CD22TCR 5 × 10^6^), *n* = 9 (CAR 5 × 10^6^, MART1TCR 3 × 10^7^, CD22TCR 3 × 10^7^, and PBS). (G) Pooled data from two independent experiments with total biological replicates: *n* = 10 (CAR and CAR + PBMC), *n* = 9 (CD22TCR, CD22TCR + PBMC, CD22TCR + exCD8αβ, and CD22TCR + exCD8αβ + PBMC), *n* = 6 (MART1TCR + exCD8αβ + PBMC), *n* = 5 (MART1 + exCD8αβ), *n* = 4 (PBS + PBMC), and *n* = 3 (PBS). *P < 0.05, **P < 0.01, ***P < 0.001, and ****P < 0.0001, by one-way ANOVA with Holm-Sidak correction (B), Mann-Whitney test (E), and Kruskal-Wallis test with Dunn’s correction [(D) and (G)]. Illustration [mouse in (F)] was made with BioRender.com.

Given the differential in vitro transcriptional and functional responses comparing CAR- to TCR-T, in vivo activities and the optimal cell dose of respective cell products may be different. First, dose-titration experiments were performed to identify the ranges of “model-therapeutic dose,” defined as the lowest cell dose necessary for leukemia control in the model. CD22 TCR-T at doses of 1 × 10^7^ to 3 × 10^7^ cells per mouse were necessary to control leukemia (as previously shown in Figs. 4 and 5 and fig. S8). In contrast, CD22 CAR-T 5 × 10^6^ cells per mouse mediated statistically equivalent leukemia control as the TCR-T 3 × 10^7^ cells per mouse and TCR-T + exCD8αβ 1 × 10^7^ cells per mouse (Fig. 7, A to D, and fig. S16, A and B). CAR-T at lower cell doses were not able to control leukemia in the model.

On the basis of weight-based dose conversion calculation, the in vivo experimental doses proportional to human clinical doses are estimated to be approximately 1 × 10^4^ to 2 × 10^4^ cells per mouse for CD22 CAR-T (*10*, *17*, *54*). Although there are no B cell antigen-directed TCR-T in clinic, various TCR-T for humans are given at the doses proportional to 3 × 10^7^ cells per mouse (datasheet S1). The CD22 CAR-T model-therapeutic dose of 5 × 10^6^ cells per mouse is approximately 250- to 500-fold above the dose proportional to what can be safely given to humans, while the model-therapeutic doses of CD22 TCR-T with or without exCD8αβ are proportional to clinical doses.

Leukemia eventually relapsed in mice treated with CD22 CAR-T 5 × 10^6^ cells per mouse, which was characterized by growth of leukemia with reduced cell-surface CD22 expression and retained HLA-A2 expression (fig. S16, C to E). At a later time point of leukemia progression, CD22 CAR-T that trafficked to spleens exhibited a trend toward having more differentiated phenotypes compared to the CD22 TCR-T (figs. S17 and S18), although it is challenging to make a biological inference about T cell phenotypic data in xenograft models given confounding by xenogeneic reactions.

Next, circulating cytokine levels in recipients that received each cell product at the model-therapeutic dose were evaluated. Treatment of mice with CD22 CAR-T at the model-therapeutic dose was associated with increased circulating IFN-γ levels 2 days after T cell infusion (Fig. 7E and fig. S19A). In contrast, circulating IFN-γ levels in the CD22 TCR-T recipients did not elevate despite the equivalent antileukemia responses. In addition to IFN-γ, levels of other cytokines also increased after CD22 CAR-T treatment, which was reminiscent of cytokine elevations seen in humans during CRS (Fig. 7G and fig. S19B).

Recipients’ myeloid cell populations that respond to the soluble mediators secreted by adoptively transferred T cells have been shown to play critical roles in the establishment of cytokine-driven systemic inflammatory toxicities after CAR-T infusion (*55*, *56*). Therefore, we next evaluated mice that were infused with HLA-A*02:01^+^ human peripheral blood mononuclear cells (PBMCs) autologous to the T cells (Fig. 7F). In the presence of PBMCs including myeloid lineage cells, CAR-T recipient mice experienced more pronounced increases in the levels of various inflammatory cytokines including those generally derived from myeloid cells, such as IL-12p70 (Fig. 7G and fig. S19B). In contrast, TCR-T treatment did not cause similar augmentation in cytokine levels in the presence of PBMCs. Together, these data demonstrate that CD22 TCR-T mediate antileukemia activity at the dose proportional to estimated human clinical dose without provoking cell dose–dependent inflammatory responses seen with CD22 CAR-T treatment.

## DISCUSSION

Our work supports the concept that TCR-T can mediate cytotoxicity with less proinflammatory cytokine production compared to CAR-T in the equalized disease context. Consistent with prior reports of various self-antigen–directed TCRs isolated from human allogeneic TCR repertoire (*57*–*62*), our study serves to corroborate the potential therapeutic values of allogeneic B cell antigen-targeting TCR-T. Our work further extended the potential benefits of TCR-T to the standpoint of mitigating systemic inflammatory toxicity, which is one of the known challenges of existing CAR-T therapies.

In our model, although the source of antigen CD22 was the same for CAR and TCR, the molecules recognized by the respective receptors were different—one being the CD22 cell-surface protein and the other being the CD22-derived peptide presented on HLA-A*02:01. The abundance and the density of each molecule on the cells are likely unequal; therefore, CD22 CAR-T and CD22 TCR-T are encountering different amounts of targets. This limited our ability to quantitatively compare the antigen sensitivity of the CAR to the TCR at a level of single antigen-receptor interaction. However, comparing receptors at a lower resolution of cell-cell interaction level, as in this study, is informative for studying clinic-oriented questions (such as toxicity) because the amount of target molecules naturally expressed by the leukemia cell is likely inconstant over time depending on environmental conditions in clinical scenarios [such as CD22 internalization after antibody/CAR engagement (*63*) and pMHC upregulation in response to IFN-γ]. It is important to recognize that functional responses of a T cell depend on the specific TCR clone and its cognate pMHC. The generalizability of our findings should be tested in other models involving alternative antigens. Another limitation of this study is the use of xenograft models in immunodeficient mice, which do not approximate the clinical contexts in which complex interactions between adoptively transferred cells, soluble mediators, and recipients’ cells culminate in systemic inflammatory toxicities. Xenogeneic graft-versus-host disease further compromises reliable evaluation of long-term in vivo outcomes and T cell biology. Nonetheless, the comparison of CAR to TCR as a function of exposure to an equalized target cell illuminated how T cells activated through each receptor differ in their initial transcriptional and functional responses in the same disease context.

CD22 TCR-T exerted superior in vitro cytotoxicity against low-antigen tumors compared to CD22 CAR-T (Fig. 6), consistent with works by others describing the relative insensitivity of CAR-T (*34*–*37*). This observation suggests that potential translational benefits of CD22 TCR-T may also extend to combating low-antigen relapses after CAR-T cell therapies (*39*, *64*), in addition to mitigating the risk of inflammatory toxicities. Cell line xenograft data from the current study provided initial insights, but further studies in patient-derived xenograft models, and ultimately clinical trials, would be necessary to establish whether TCR-T can indeed ameliorate CAR-antigen escape issues.

The concept of CD8 coreceptor addition to improve anticancer functions of TCR-T has been used by other groups (*65*–*72*). Consistent with prior works, CD8αβ-coexpressing CD22 TCR-T had better antileukemia cytotoxicity compared to TCR-T alone. However, adding CD8αβ to TCR-T in a therapeutic context requires caution because the effects of CD8αβ on T cell effector functions vary depending on the TCR; removing CD8αβ has been shown to improve antitumor functions of high-affinity TCR-Ts (*73*) and the risk of cross-reactivity changed depending on affinities between pMHC and TCR and between MHC and CD8 (*74*–*78*). Additional CD8αβ may be beneficial when the TCR’s affinity is relatively low, as in the settings of TCRs against cancer-associated self-antigens. Notably, the notion that there is an optimal “total” signal input, which is achieved by the sum of effects of all signaling-associated molecules rather than by the antigen-receptor affinity alone, also applies to CAR (*79*, *80*).

CD4^+^ CD22 CAR-T generally secreted larger amounts of various cytokines compared to the CD8^+^ counterparts (Fig. 6C and fig. S10), which is congruent with the reports that CD4^+^ CAR-T is responsible for CRS (*81*, *82*). Multifaceted roles played by CD4^+^ T cells for cancer controls span from helper functions to direct cytotoxicity (*83*–*87*), and cell products containing CD4^+^ (*4*, *88*–*91*) achieves better antitumor functions compared to CD8^+^-only products in some contexts. Recent studies showed that CD4^+^ CAR-T with less direct granule-mediated cytotoxicity relied on an IFN-γ–mediated antitumor mechanism while CD8^+^ CAR-T that predominantly mediated direct target lysis relied less on IFN-γ (*92*, *93*). In line with the finding, we have previously shown that CAR-T defective in perforin overproduced cytokines in a syngeneic murine system (*94*). IFN-γ not only was necessary for antileukemia control in perforin-deficient settings but also ultimately contributed to inflammatory toxicities. Some CD19 CAR-T–treated patients who experienced severe CRS were found to have genetic variants of STXBP2 (*95*), a gene involved in granule-mediated cytotoxicity pathway, further bolstering the potential link between defective granule-mediated cytotoxicity and post–CAR-T inflammation. Impaired granule-mediated cytotoxicity is well established to drive T cell–triggered systemic inflammation (*96*). It is tempting to imagine that inefficient direct cytotoxicity of adoptively transferred T cells may be associated with cytokine oversecretion, provoking inflammation in hosts.

Aside from cytokine production, various gene sets related to other antileukemia functions were either equivalently or positively enriched in TCR-T compared to CAR-T, which suggest that inflammatory responses are disproportionately overrepresented in CAR-T. This observation raises the possibility that the inflammatory transcriptional responses in T cells are the byproducts of artificial CAR signaling, which stands in contrast to the TCR signaling that has been shown to elicit granule release and cytotoxicity at lower signaling threshold compared to the threshold required for IFN-γ production (*97*). It remains unknown how the CAR signaling mechanistically induces inflammatory responses, and how the disparate pre-stimulation transcriptional states comparing CAR-T to TCR-T affected the post-stimulation inflammatory outcomes. Proximal signaling downstream of CAR has been shown to be distinct and inefficient compared to TCR (*34*, *38*, *98*). Modifying the CAR design to enhance the signaling to approximate natural TCR signaling (*38*, *99*) or to bypass proximal signaling (*100*) has been reported to improve antitumor CAR-T cytotoxicity. It may also be interesting to study whether these newer CARs with more efficient TCR-like signaling capacity would be less prone to induce inflammation.

One of many outstanding questions is how to harness cytokine-mediated antitumor effects without compromising safety because pleotropic cytokine effects are important for tumor control. Given that tumors often contain heterogeneous cell populations including antigen-negative or MHC-deficient cells, cytokine-related various killing mechanisms are invaluable to maximize curative potentials of ACT. Systemic cytokine elevations may not be a prerequisite to achieving favorable antitumor effects of ACT considering that IFN-γ broadly diffuses at tissue level (*101*). CAR-T designed to produce more cytokines have been demonstrated to mediate promising antitumor effects (*102*–*106*). However, CAR-T with augmented cytokine activities could narrow the therapeutic window depending on the clinical contexts. In our model, TCR-T executed tumor lysis with less accompanying cytokine secretion compared to CAR-T, addressing one of the clinical challenges of CAR-T infusion. Therefore, TCR-T may be a safe adjunctive or complementary treatment option. Identifying the optimal combination of CAR- and TCR-T (*61*) may be able to achieve the balance of desired antitumor effects and undesired inflammatory toxicities while synergistically overcoming heterogeneous resistance mechanisms employed by the tumor.

## MATERIALS AND METHODS

### In vitro allogeneic stimulation of human PBMCs to isolate CD22-specific TCR

Monocyte-derived DCs were generated using the adherence technique from HLA-A*02:01^+^ human PBMCs. Briefly, PBMCs were resuspended in CellGenix GMP DC medium (Cellgenix) with 1% heat-inactivated human AB serum (HS) (Gemini Bio) and cultured in a six-well TC-treated plate at 1 × 10^7^ cells per well. Following 90 min of incubation in 37°C, nonadherent cells were gently removed by swirling and pipetting with warm serum-free media. Cells adherent on the bottom of the plate were cultured overnight with Cellgenix DC medium, 1% HS with IL-4 1000 IU/ml (PeproTech), and GM-CSF 800 IU/ml (PeproTech) to make immature DCs. After culturing overnight, the following cytokines were added to the culture to achieve final concentrations of tumor necrosis factor–α (TNFα) 10 ng/ml (PeproTech), IL-1β 10 ng/ml (PeproTech), IL-6 1000 IU/ml (PeproTech), and prostaglandin E2 (PGE2) 1 μg/ml (Sigma-Aldrich) to mature DCs. Matured DCs were harvested about 24 hours later and loaded with minimal epitope CD22 peptide (FLSNDTVQL) at 1 μM for 30 to 60 min at 37°C and irradiated with 40 Gy.

CD8^+^ T cells were enriched using magnetic negative selection (Miltenyi Biotec) from an HLA-A*02:01–negative human PBMCs from the donor allogeneic to the donor of the DCs. Then, CD8^+^ T cells were incubated with CD22 tetramers conjugated with phycoerythrin (PE) at 4°C in the dark for 1 hour (the method for tetramer synthesis is found in Supplementary Methods). Then, tetramer-binding fraction was positively selected using anti-PE beads (Miltenyi Biotec) and the magnetic isolation technique. CD22 peptide–loaded and irradiated mature DCs from HLA-A*02:01^+^ donor and allogeneic CD22 tetramer–binding CD8^+^ T cells from HLA-A*02:01^−^ donor were cocultured at a ratio of 1:10 (DC 2 × 10^4^ cells per well with T cells 2 × 10^5^ cells per well) in a 96-well flat-bottom plate in RPMI-based media (RPMI, 10% HS, l-alanyl-l-glutamine dipeptide and penicillin/streptomycin 100 μg/ml) in the presence of IL-21 30 ng/ml (PeproTech): This represented the allogeneic in vitro stimulation #1. Three days later, the IL-7, IL-2, and IL-15 (all from PeproTech) were added to achieve final concentrations of IL-7 10 ng/ml, IL-2 25 IU/ml, and IL-15 2 ng/ml. When cells became confluent, cells were transferred to a 48-well plate, and fresh media containing IL-7, IL-15, and IL-2 at the same concentration were added every 3 days. Two weeks after the allogeneic in vitro stimulation #1, the second in vitro stimulation (#2) was set up: K562A2 expressing only HLA-A*02:01 but no other HLA class I or II alleles was loaded with CD22 peptide 1 μM for 30 to 60 min at 37°C and irradiated with 100 Gy, and PBMCs autologous to the CD8^+^ T cells were irradiated at 40 Gy. Peptide-loaded and irradiated K562A2 cells representing artificial APCs 1 × 10^5^ cells per well, autologous irradiated PBMCs (feeder cells) 5 × 10^6^ cells per well, and cells from each well of in vitro stimulation #1 (T cells) 1 × 10^6^ cells per well were cocultured in a 24-well plate at the ratio of 1:50:10. Three days later, IL-7, IL-2, and IL-15 were added to achieve final concentrations of IL-7 10 ng/ml, IL-2 25 IU/ml, and IL-15 2 ng/ml. Two weeks after in vitro stimulation #2, the presence of CD22 tetramer–binding CD8^+^ T cells in each coculture well was evaluated with flow cytometry analysis. CD22 tetramer–binding CD8^+^ T cells from coculture wells were magnetically isolated using positive enrichment (tetramer-PE staining and anti-PE microbeads) and were subject to single-cell TCR α/β paired sequencing (Supplementary Methods).

### Generation of TCR- and CAR-expression vectors

MSGV1 gammaretroviral bicistronic vector encoding the CD22 TCR was designed as previously described (*42*–*44*). The vector encoded TCR β chain and TCR α chain separated by a cleavable linker composed of a P2A and a furin recognition site sequence. The constant region was substituted with the mouse counterpart with modifications. HPV-16 E7 TCR (*42*), MART 1 TCR (clone DMF5) (*107*), and KK-LC-1 TCR (*108*) used as negative controls in this project, and EBV LMP2 TCR (*45*) against a viral antigen coexpressed with CD22 by EBV^+^ B cell malignancies (fig. S3), were also cloned in the identical MSGV1 backbone. The design of cleavable linker and mouse constant regions was the same across all TCRs. The design of CD22 CAR with scFv clone m971 with the 4-1BB costimulatory domain was previously described (*10*, *52*), and the CD22 CAR sequence was cloned into the MSGV1 backbone identical to that of the TCR. Gene synthesis, subcloning, and plasmid preparation were performed by GenScript Biotech. CD8 coreceptor-expression vectors are described in Supplementary Methods. Generation of gamma-retroviral supernatant and receptor-transduced T cells is described in Supplementary Methods.

### T cell in vitro functional assays

For the assessment of select in vitro cytokine production (Figs. 2, D and E, 3, 5, B and C, and 6C and figs. S4 and S10), T cells were cocultured with target cell lines at an E:T ratio of 1:1, 5 × 10^4^ cells each in a 96-well U-bottom plate at a final volume of 200 μl per well. After overnight coculture, supernatant was harvested and the levels of cytokines were assessed using enzyme-linked immunosorbent assay (ELISA; IFN-gamma DuoSet ELISA DY285, R&D; Human TNF-alpha DuoSet ELISA DY210, R&D; Human CCL2/MCP-1 DuoSet ELISA DY279, R&D; Human GM-CSF DuoSet ELISA, DY215, R&D; Human IL-8/CXCL8 DuoSet ELISA DY208, R&D; Multiscan FC Microplate Reader, Thermo Fisher Scientific). Detailed methods of intracellular cytokine staining, CD107a assay, and in vitro cytotoxicity assay are described in Supplementary Methods.

### Murine xenograft models of adoptive TCR-T and CAR-T therapy

Age- and sex-matched NSG mice were intravenously injected with HLA-A*02:01^+^ and CD22^+^ B cell leukemia (BV173) or lymphoma (DG-75) cell lines transduced to express firefly luciferase. Seven days after tumor cell infusion, T cells were adoptively transferred (intravenous) at the dose indicated in figure legends. Transduction efficiency across all receptor groups were above 80 to 90% (i.e., less than 10% variability in transduction efficiency across groups within an experiment) (fig. S20), and the cell dose was adjusted for transduction efficiency. All intravenous injections were performed via tail vein. Chronological changes of in vivo tumor burden were assessed with bioluminescent imaging of mice using In Vivo Imaging System (IVIS) (Promega). A magnified color scale of the IVIS luminescent image output (radiance, photons/s/cm^2^/sr) is shown in fig. S21. Each independent experiment was performed with T cell products manufactured from an independent donor. In Fig. 7, F and G, PBMCs given on day −1 were given via both intravenous (1 × 10^7^) and intraperitoneal (2.5 × 10^7^) routes for a total dose of 3.5 × 10^7^ cells per mouse to avoid the risk of cell embolism associated with high-dose intravenous cell infusions.

### Bulk RNA-seq

T cells were cocultured with HLA-A*02:01^+^ leukemia cells, BV173, at an E:T ratio of 1:1 (2 × 10^7^ cells each per well) in a 24-well plate. Following 6 hours of coculture, leukemia was depleted from each sample using anti–HLA-A2-PE antibody and anti-PE microbeads, and the purity of T cells in the post–leukemia-depletion samples were confirmed with flow cytometry analysis. T cells were snap frozen on dry ice and thawed once immediately before RNA extraction using the Qiagen RNeasy Plus Mini Kit (Qiagen). The experiment was set up in three biological replicates and RNA extraction was executed in a single batch. The concentration of RNA was measured with Nanodrop (Invitrogen, then QC was performed with Agilent TapeStation by the CCR Genomics Core). Bulk RNA-seq was performed at the CCR Sequencing Facility using the NovaSeq 6000 SP using Illumina Stranded mRNA Prep and paired-end sequencing with a reference genome of hg38. The FASTQ files with paired-end reads were processed using Partek Flow. The raw reads were aligned to the reference genome using STAR and the aligned reads were quantified to the annotation model using Partek E/M. The normalized counts per million (CPM) from Partek Flow were used for expression quantification. GSEA was further performed, with a false discovery rate (FDR) threshold of less than 0.25 being considered significant. Unsupervised clustering and visualization with a heatmap were performed with the ggplot2 package of R (RStudio 4.2.2). The RNA-seq data are available in Gene Expression Omnibus (GEO) accession GSE255323.

### Statistical analyses

Continuous variables are presented as means ± SD. Mann-Whitney test was used for two-group comparisons. For comparison among groups of three or more, the Brown-Forsythe test and the Shapiro-Wilk test were used to test the equal variance assumption and normality assumption. Then, data appropriate for parametric methods and nonparametric methods were analyzed with one-way analysis of variance (ANOVA) and Kruskal-Wallis test, respectively. *P* values were adjusted for multiple comparisons using the Holm-Sidak method (for one-way ANOVA) or Dunn’s method (for Kruskal-Wallis). Two-tailed *P* values < 0.05 were considered significant. GraphPad Prism version 9 (GraphPad Software) was used for analyses.

## Supplementary Materials

### The PDF file includes:

Supplementary Methods Tables S1 to S3 Figs. S1 to S21 Legend for datasheet S1 References

### Other Supplementary Material for this manuscript includes the following:

Datasheet S1
