# Supplementary material for: CD22 TCR-engineered T cells exert antileukemia cytotoxicity without causing inflammatory responses
Source: Sci Adv. 2025 Apr 9;11(15):eadq4297. doi: 10.1126/sciadv.adq4297 (PMC11980841; doi:10.1126/sciadv.adq4297)
Supplement: Supplementary file 1 — Supplementary Methods Tables S1 to S3 Figs. S1 to S21 Legend for datasheet S1 References [file sciadv.adq4297_sm.pdf]

Science Advances
AAAS

Supplementary Materials for

CD22 TCR-engineered T cells exert antileukemia cytotoxicity without causing inflammatory responses

Kilyna A. Nguyen *et al.*

Corresponding author: Kazusa Ishii, kazusa.ishii@nih.gov

Sci. Adv. **11**, eadq4297 (2025)
DOI: 10.1126/sciadv.adq4297

The PDF file includes:

Supplementary Methods
Figs. S1 to S21
Tables S1 to S3
Legend for datasheet S1
References

Other Supplementary Material for this manuscript includes the following:

Datasheet S1

Supplementary Methods

Mice

NOD.Cg-Prkdc^{scid} Il2rg ^{tm1Wjl}/SzJ (NSG) mice were obtained through NCI Animal Resource Program (Frederick, MD, USA). All animals were cared in accordance with the protocol (GMB-011) approved by the Animal Care and Use Committee at the National Cancer Institute and housed in a standard clean facility. All experiments were performed using sex-matched mice between 5 and 7 weeks of age.

Cell lines

K562, T2, RPMI1788, NALM6, JVM2, DG-75, CCRF-SB, JeKo-1, Raji, Ramos, CA46, LAMA84, THP-1, CaSki, MOLT-4, U266B1, and RPMI6666 were purchased from ATCC. Cell line BV173 was purchased from DSMZ. K562A2 is K562 transduced to express HLA-A*02:01, K562A2 CD22$_{p228-236}$ and E7$_{p11-19}$ are K562A2 loaded with respective peptides at 1 μM, K562A2 CD22 and K562A2 CD19 are K562A2 transduced to express full-length CD22 or CD19 protein, respectively. Cell lines transduced to express firefly luciferase (BV173-ffLuc and DG-75-ffLuc) were made by transducing the cells with firefly luciferase tagged with GFP, then sorting for the GFP+ fraction. DM3939, LD3967, SS4050, DC3809, TM3777, JK4156, SS3853, LCL-S, LCL-C, LCL-R, LCL-K, 43MW, 23FA, D37, 19MW, D28, D731, D83, 56FW, 35FW, and AW3775 are EBV-immortalized lymphoblastoid cell lines (EBV-LCL). Production of EBV-LCL and HLA typing were previously described (108). Cell lines were tested negative for mycoplasma using the Mycoplasma PCR Detection Kit (Applied Biological Materials Inc.)

Human PBMC / lymphocytes

De-identified HLA-typed PBMCs in select experiments were obtained through the Center for Immuno-Oncology clinical trial (NCT02821806) at the NCI in which study participants provided written informed consent. High-resolution HLA class I typing data were available through the NIH Clinical Center HLA laboratory (108). T cells transduced with either TCR or CAR were made from human buffy coats from healthy volunteer blood donors as byproducts of allogeneic blood donation at the NIH Department of Transfusion Medicine (DTM). Blood donors provided written informed consent for the use of their blood for research purposes and blood samples were de-identified prior to distribution from the DTM.

Cell culture media and incubator condition

Human cancer cell lines were cultured in RPMI 1640 with 10% heat-inactivated fetal calf serum (Omega Scientific), L-alanyl-L-glutamine dipeptide (Gibco, Life Technologies, GlutaMAX diluted to 1x), nonessential amino acids (Gibco, Life Technologies, MEM-NEAA diluted to 1x), sodium pyruvate 1 mM (Gibco, Life Technologies), HEPES 15 mM (Gibco, Life Technologies), penicillin 100 units/mL and streptomycin 100 μg/mL (Gibco, Life Technologies). For activation, expansion, and culturing of human T cells for the purpose of manufacturing TCR- or CAR-transduced T cells, "T cell media" was used containing AIM-V media (Gibco, Life Technologies) supplemented with 5% heat-inactivated fetal calf serum, L-alanyl-L-glutamine dipeptide (Gibco, Life Technologies, GlutaMAX diluted to 1x), nonessential amino acids (Gibco, Life Technologies, MEM-NEAA diluted to 1x), sodium pyruvate 1 mM (Gibco, Life Technologies), HEPES 15 mM (Gibco, Life Technologies), penicillin 100 units/mL and streptomycin 100 μg/mL (Gibco, Life Technologies). Cell culture media condition for an allogeneic *in vitro* stimulation (Figure 1) is

described in the Materials and Methods section "*In vitro allogeneic stimulation of human PBMC to isolate CD22-specific TCR*". All cultures were performed in humidified incubator at 37°C with 5% CO2.

UV-activated peptide exchange and p-MHC tetramer synthesis

UV-mediated ligand exchange and p-MHC tetramer production were performed as previously described (109) using Flex-T HLA-A*02:01 Monomer UVX (BioLegend), streptavidin-PE (Life Technologies), and a UV lamp (Camag).

Single cell TCR paired sequencing

Single cell TCR alpha/beta paired sequencing and all analyses were performed at the National Cancer Institute Center for Cancer Research Single Cell Analysis Facility. This work utilized the computational resources of the NIH HPC Biowulf cluster (http://hpc.nih.gov). The sequencing of the donor 146 T cells yielded productive V(D)J spanning pairs with only one predominant clonotype (Supplementary Table S1).

Single Cell – Partitioning and Library Preparation: Single cell suspensions of T cells stored in RNA Protect (Qiagen) were aliquoted 25 µL each into several microcentrifuge tubes and volumed up to 1 mL with PBS. These were centrifuged at 300g for 5 minutes, supernatant removed, and then cells were resuspended in approximately 50 µL of remaining buffer volume. Cell counts were measured using a fluorescent cell counter with propidium iodide and acridine orange (LunaFL, Logos Biosystems). Cell suspensions were loaded at maximum volume onto the 10x Genomics Chromium platform using the 5' v1 immune profiling chemistry (10x Genomics). Following partitions, reverse transcription, and cDNA amplification, TCR enrichment libraries were prepared according to vendor recommendations.

Single Cell – Sequencing: Sequencing of single cell human TCR libraries were performed on an Illumina MiSeq instrument with paired end 150bp reads and an 8bp read for sample indices. Samples were sequenced to over 2,000 reads per cell on average for the TCR libraries for all samples.

Single Cell – Data Processing: Data was processed using the 10x Genomics cellranger pipeline (v3.0.2) to demultiplex reads and then align reads to a GRCh38 VDJ reference for single cell TCR data (refdata-cellranger-vdj-GRCh38-alts-ensembl-2.0.0). UMI-adjusted aligned reads were used to generate a TCR clonotype matrix that was used for downstream analysis.

Gamma-retroviral supernatant production

293GP cells were co-transfected with a plasmid encoding the TCR and a plasmid encoding the envelope RD114 using the lipofectamine 3000 (Life Technologies) following the manufacturer's protocol. Viral supernatant was collected 48, 72 and 96 hours after the transfection and frozen and stored in -80°C until use.

Human T cell transduction

Non-tissue-culture treated plates were coated with anti-CD3 antibody (Miltenyi) 1 µg/mL and anti-CD28 antibody (clone CD28.2, BD Biosciences) 1 µg/mL for 3 hours in 37°C. Then, human PBMCs resuspended in T cell media were added to the plate. Approximately 16-24 hours later, rh-

IL-2 100 IU/mL (Clinigen / Novartis) was added to the media and further cultured for another day. After two days of T cell activation, retronectin (Takara Bio USA)-coated non-tissue-culture-treated plates were spun with retroviral supernatant for 2 hours at 2000 G and 32°C. Then, activated T cells were added to the plate. T cells were cultured for a total of 6-12 days after the completion of transduction in media containing IL-2 200 IU/mL before being assessed for transduction efficiency and used for intended assays. Each independent experiment was performed with an independent set of PBMC donors. All T cell experimental groups within a given *in vivo* experiment used the identical donor (for example, TCR- and CAR-T cells within a given experiment were made from the same donor). Cell doses were adjusted for transduction efficiency, which had variability of less than 10% across receptor-T cell groups (Supplementary Figure S20).

CD8 co-receptor constructs

CD8 alpha chain sequence (UniProt, P01732-1) and CD8 beta chain sequence (UniProt, P10966-1) were cloned into the gamma retroviral vector backbone (MSGV1). TCR-T cells with additional CD8αα homodimers were made by co-transducing the TCR-T cells with CD8α-encoding vector. TCR-T cells with additional CD8αβ heterodimers were made by co-transducing the TCR-T cells with bicistronic vector encoding the insert sequence in the order (5' to 3') of CD8 beta chain and CD8 alpha chain.

Flow cytometry

CAR detection was performed using biotinylated protein L (Thermo Scientific) and streptavidin conjugated with PE or APC (BD Biosciences) as previously described (110). Antibodies and reagents used for flow cytometry analysis are listed in Supplementary Table S3. Samples were analyzed on BD LSR Fortessa (BD Biosciences) or ACEA NovoCyte Flow Cytometer (ACEA Biosciences, Agilent). FACS Diva and FlowJo version 10 (FlowJo, BD Biosciences) were used for data collection and analysis, respectively.

T cell in vitro cytotoxicity assay

Flow cytometry-based assessment of *in vitro* cytotoxicity was performed as previously described with modifications (111-113). Antigen-negative control cells HLA-A2+/CD22- (K562A2) were stained with CellTrace Far Red (Invitrogen), and HLA-A2+/CD22+ target cells (BV173, RPMI1788, or JVM2) were stained with CellTrace Yellow (Invitrogen) following manufacturer's recommendations. Stained antigen-negative control cells and target cells were mixed at 1:1 ratio (5e4 cells each) in 96-well U-bottom plate. Then effector cells were added at an E:T ratios of 20:1, 10:1, 5:1, and 2.5:1. Some wells contained only tumor cells without an addition of effector cells. Co-culture was set up in 3 technical replicates. Four hours after the co-cultures, cells were stained with Live/Dead Aqua (Invitrogen), fixed with 4% PFA, and washed twice before samples were ran on flow cytometry. Cytotoxicity was calculated as below to account for spontaneous death of target cells: %survival = (%live target cells / %live antigen-negative control cells) x100; corrected %survival = (%survival / (mean %survival in the absence of effectors)) x100; %cytotoxicity = 100 – corrected %survival.

HLA-A2-peptide-binding assay

Whether a peptide is likely to bind or not to HLA-A2 was assessed using the transporter associated with the antigen (TAP)-deficient and HLA-A*02:01+ cell line T2 as described previously (114) with some modifications. T2 cells were washed and resuspended in serum-free media and loaded with each peptide at 100 µg/mL overnight in 37C^{o}, then cell-surface HLA-A2 expression was assessed with flow cytometry. Control peptides with known HLA class I binding used in the assay were an HLA-A*02:01-restricted HPV-16 $E7_{p11-19}$ peptide (42) and an HLA-A*01:01-restricted $KKLC1_{p52-60}$ peptide (108).

Intracellular cytokine staining and CD107a degranulation assay

Target cells (Figure 1D and Figure 2F) were co-cultured with effector cells at an E:T ratio of 2:1 in 96-well U-bottom plate (1e5 effector and 5e4 target cells each per well). For the target cell conditions requiring peptide loading, target cells were loaded with respective peptide at 1 µM concentration for 30 minutes in 37°C, then washed twice before being used for co-culture. One hour after starting the co-culture, brefeldin A and monensin (eBioscience Protein Transport Inhibitor Cocktail 500x, Invitrogen) were added to co-culture well at the final concentration of 1x of the reagent as recommended by the manufacturer. Anti-CD107a antibody or isotype control were added to each well (1 µL per 200 µL of total final volume). Three to four hours after adding protein transport inhibitor cocktail (i.e. 4-5 hours after starting co-culture), cells were washed once, then stained with fixable live-dead dye (Live/Dead Fixable Aqua, Invitrogen) followed by antibody staining for cell surface antigens in 4°C in the dark for 30 min. Cell-surface stained samples were then washed twice before fixed and permeabilized using BD Cytofix/Cytoperm Fixation Permeabilization kit (BD Biosciences) for 20 minutes in 4°C. After two washing steps, samples were stained with antibodies against IFNγ, IL-2, and TNFα for 30 minutes in 4°C, then washed again twice with BD Perm/Wash buffer and analyzed with flow cytometry.

Murine serum cytokine measurement

Serum samples were harvested 2 days after T cell infusion from each mouse and snap frozen on dry ice. Samples were thawed once immediately prior to cytokine measurement using the Meso Scale Discovery (Meso Scale Diagnostics) assay, which was performed at Clinical Support Laboratory of the NCI at Frederick.

Peptides and Peptide-MHC IC50 prediction

HPLC-purified peptides were synthesized by Peptide 2.0 Inc and GenScript Biotech. The HLA-A*02:01 binding predictions were performed on 9/5/2020 using the IEDB analysis resource Consensus tool (115) (www.iedb.org, (40)), which combines predictions from ANN aka NetMHC (4.0) (116-118), SMM (119) and Comblib (120).

In silico search of cross-reactive candidate peptides

Candidate peptides from human proteome were curated through *in silico* searches following the previously published framework (45) which consisted of multi-layer approaches. Briefly, ScanProsite webtool was used to identify peptides derived from human proteome (121) based on the *in silico* search rules of 1) TCR/peptide-HLA*02:01 contact motif-guided search, and 2) amino acid substitution-based contact motif-unguided search. In alanine and glycine scanning (Figure 3A, Supplementary Figure S4A), substitution of non-anchor residues 3, 4, 5, and 8 almost completely abrogated IFNγ production by T cells, suggesting these are residues critical for TCR contact. Based

on this motif information, *in silico* search was carried out to identify peptides sharing the contact motif sequence from human proteome. Search was performed for x-x-S-N-D-x-x-Q-x (Search 1 of Supplementary Figure S4B) where x indicates that any one of natural amino acids are allowed. These *in silico* search rules provided 4 unique peptides with predicted HLA-A*02:01 IC_{50} of <10,000 nM. Because cross-reactive peptides may not necessarily share the TCR recognition motif sequences, *in silico* search rule was expanded to allow substitution of each residue with amino acids with physicochemical similarity as previously described (45). *In silico* search input sequence was [FWY]-[VILM]-[SATG]-[DENQ]-[DENQ]-[SATG]-[VILM]-[DENQ]-[VILM] (Search 2a of Supplementary Figure S4B). The amino acids in brackets [] indicate the list of amino acids considered substitutable in the position based on size, hydrophobicity, and the property of side chains (45, 122-124). Search 2a identified 7 peptides with HLA-A*02:01 IC_{50} of <10,000 nM. *In silico* search was further expanded for an abundance of precaution, using more lenient substitution rule in which large aromatic amino acids ([FWY]) and non-aromatic hydrophobic amino acids with medium-large mass ([VILM]) were grouped together: [FWYVILM]-[FWYVILM]-[SATG]-[DENQ]-[DENQ]-[SATG]-[FWYVILM]-[DENQ]-[FWYVILM] (Search 2b of Supplementary Figure S4B). Search 2b resulted in 34 peptides, including 27 additional unique peptides with HLA-A*02:01 IC_{50} of <10,000 nM. Finally, *in silico* search using protein BLAST (blast.ncbi.nlm.nih.gov) with lenient parameters provided one peptide shared with the Search 2b output and 132 additional unique candidate peptides unfiltered for peptide-HLA-A*02:01 IC_{50} (Search 3 of Supplementary Figure S4B). In BLAST, query sequence was the $CD22_{p228\text{-}236}$ peptide sequence (FLSNDTVQL), and the search database was set as "Non-redundant protein sequences (nr)" with a filter for homo sapiens (taxid:9606). The "blastp" algorithm was chosen, and the algorithm parameters were set as follows: max target sequences of 500, check the box for "automatically adjust parameters for short input sequences" which led the blastp program to automatically use PAM30 matrix. All these results combined, a total of 170 unique peptides were selected as cross-reactivity screening candidates. Search input definitions are described in Supplementary Figure S4B, and amino acid sequences of resulting unique candidate peptides are listed in x-axis of Figure 3B and Supplementary Figure S4C.

ScanProsite was accessed between August 1, 2019 and January 2, 2023. In ScanProsite search, reference protein sequence database selected were UniProtKB/Swiss-Prot including isoforms, and search was restricted only to homo sapiens. Protein BLAST was accessed between March 29, 2021 and August 3, 2023 (121, 125).

Supplementary Figure S1

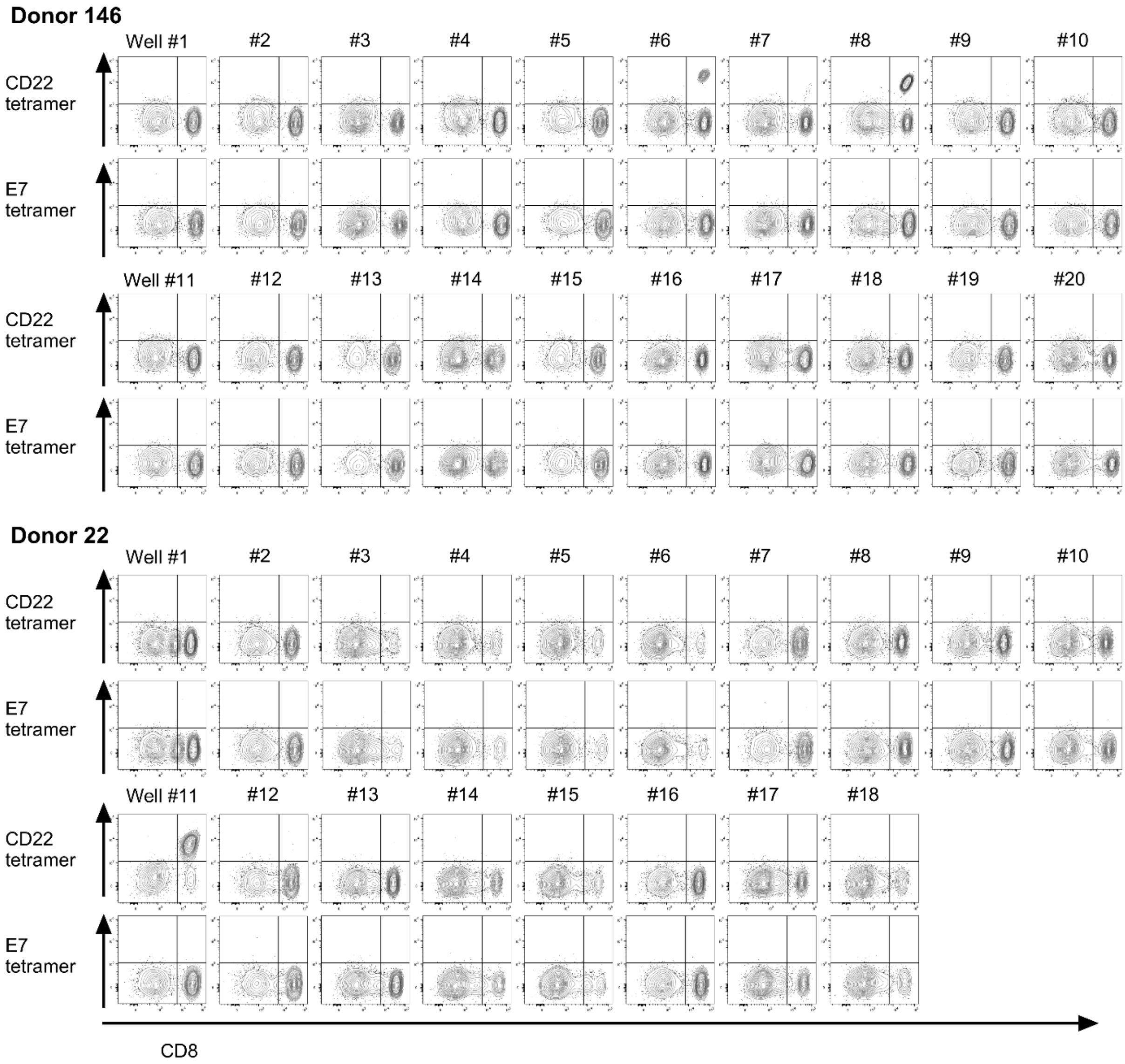

Fig. S1. Presence of CD22 tetramer-binding CD8+ T cells in HLA-A*02:01 negative PBMCs

As shown in the Fig. 1A, HLA-A*02:01 negative donor's PBMCs were stimulated with an allogeneic monocyte-derived dendritic cells (DC) from HLA-A*02:01+ donors loaded with CD22 minimal epitope peptide, then with K562A2 loaded with the CD22 peptide. Each *in vitro* allogeneic stimulation well was set up at PBMC to DC ratio of 10:1 as detailed in the Methods. *In vitro* stimulation experimental setup amounted to a total of 20 wells for the Donor 146 and 18 wells for Donor 22. Cells in each well were assessed for the presence of T cell populations that bind to CD22 tetramers and E7 tetramers using flow cytometry analysis. Dot plots from each well are shown.

Supplementary Figure S2

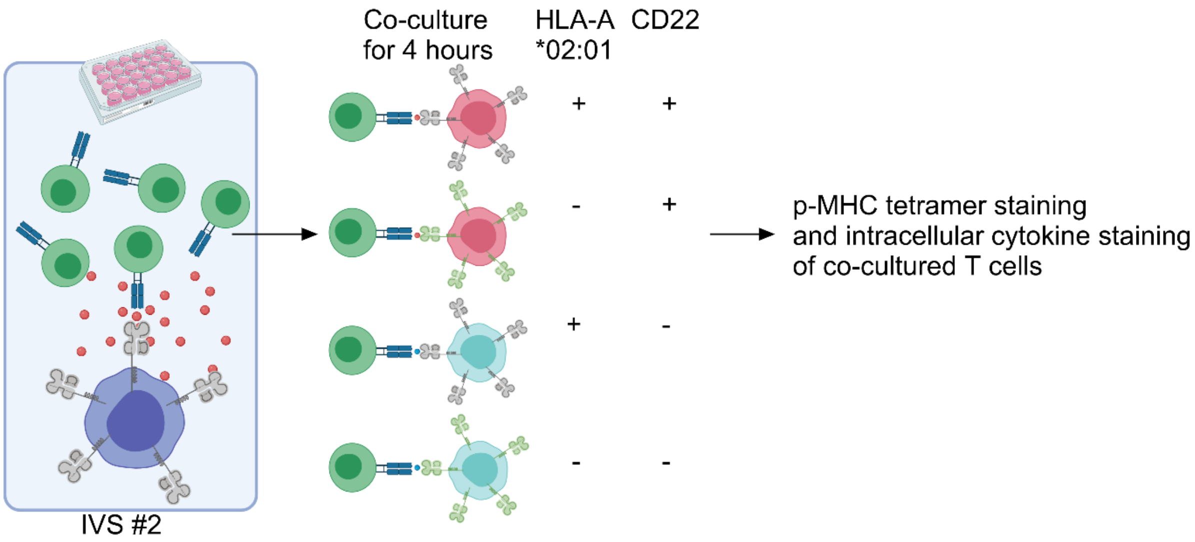

Fig. S2. Scheme of coculture assay performed upon completion of two rounds of *in vitro* allogeneic stimulation

Polyclonal T cells that expanded after two rounds of *in vitro* stimulation in each well were cocultured with a panel of target cell lines covering different combinations of HLA-A*02:01 and CD22 expression status. Four hours after the coculture, the presence of CD22 tetramer-binding populations and IFNγ production were assessed with flow cytometry. Illustrations were made with BioRender.com.

Supplementary Figure S3

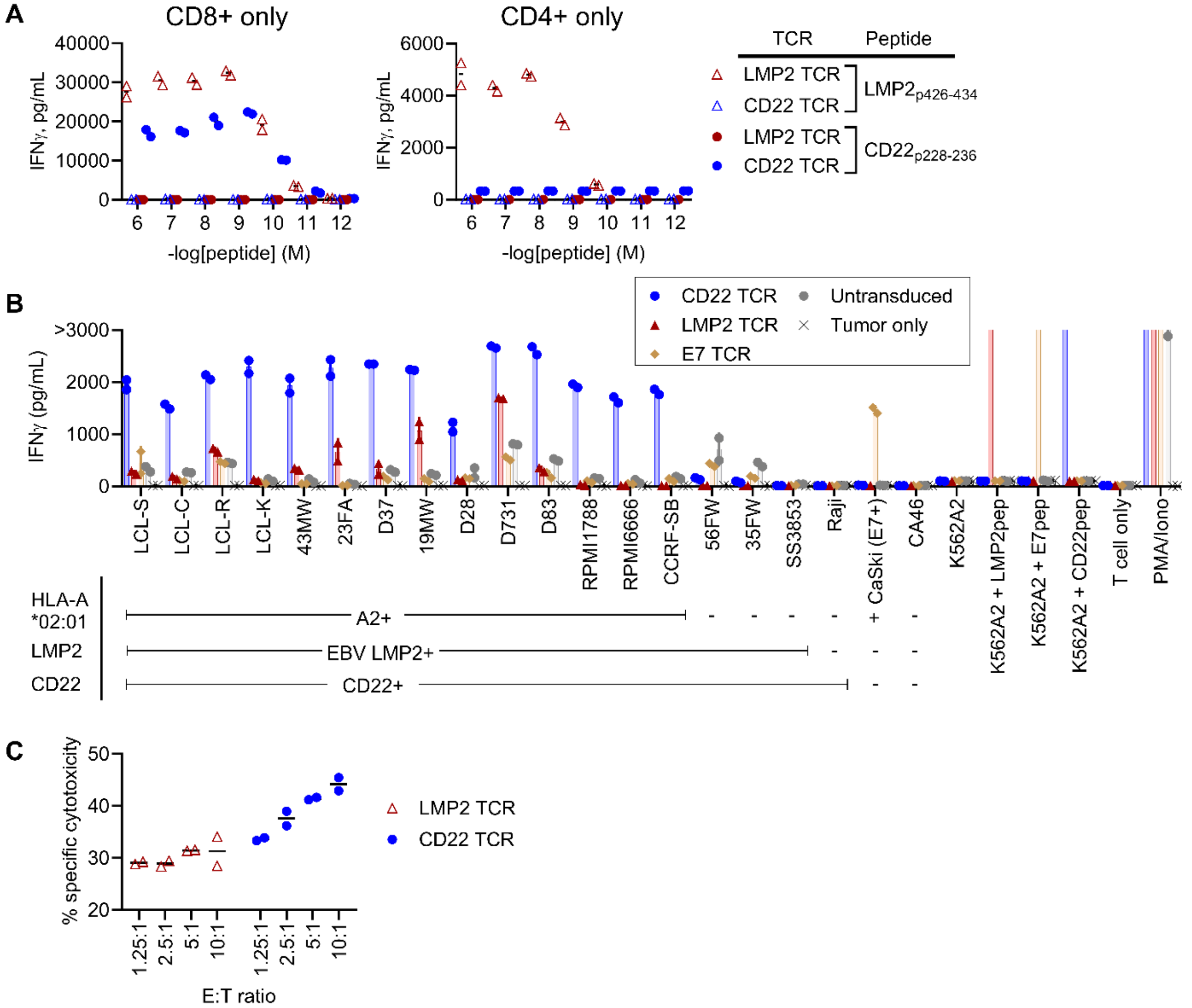

Fig. S3. CD22 TCR-T cells, compared to EBV LMP2 TCR-T cells, demonstrate superior IFNγ responses and *in vitro* cytotoxicity against EBV+ lymphoblastoid cells and leukemia / lymphoma cell lines that naturally co-express both CD22 and EBV LMP2

(**A**) CD8+ and CD4+ enriched T cells transduced to express either the CD22 TCR or EBV LMP2 TCR were cocultured with K562A2 loaded with the indicated peptides (E:T = 1:1). IFNγ levels in the overnight coculture were measured by ELISA. (**B**) CD22 TCR-T and LMP2 TCR-T (bulk, CD3+) were cocultured overnight with the indicated cell lines (E:T = 1:1). IFNγ levels were measured by ELISA. The CD22, EBV LMP2, and HLA-A*02:01 expression of each cell line is indicated as + or –. K562A2 cells loaded with 1 µM of minimal epitope peptides for each TCR are indicated as K562A2 + LMP2pep, + E7pep, and + CD22pep on the x-axis. (**C**) *In vitro* cytotoxicity of CD22 CAR-T and EBV LMP2 TCR-T against a cell line JVM2 (HLA-A*02:01+ EBV+) after 4 hours of coculture. The method of the *in vitro* cytotoxicity assay is described in the Supplementary Methods. Representative figures from 3 independent experiments. Technical replicates: n=2 (A-C).

Supplementary Figure S4

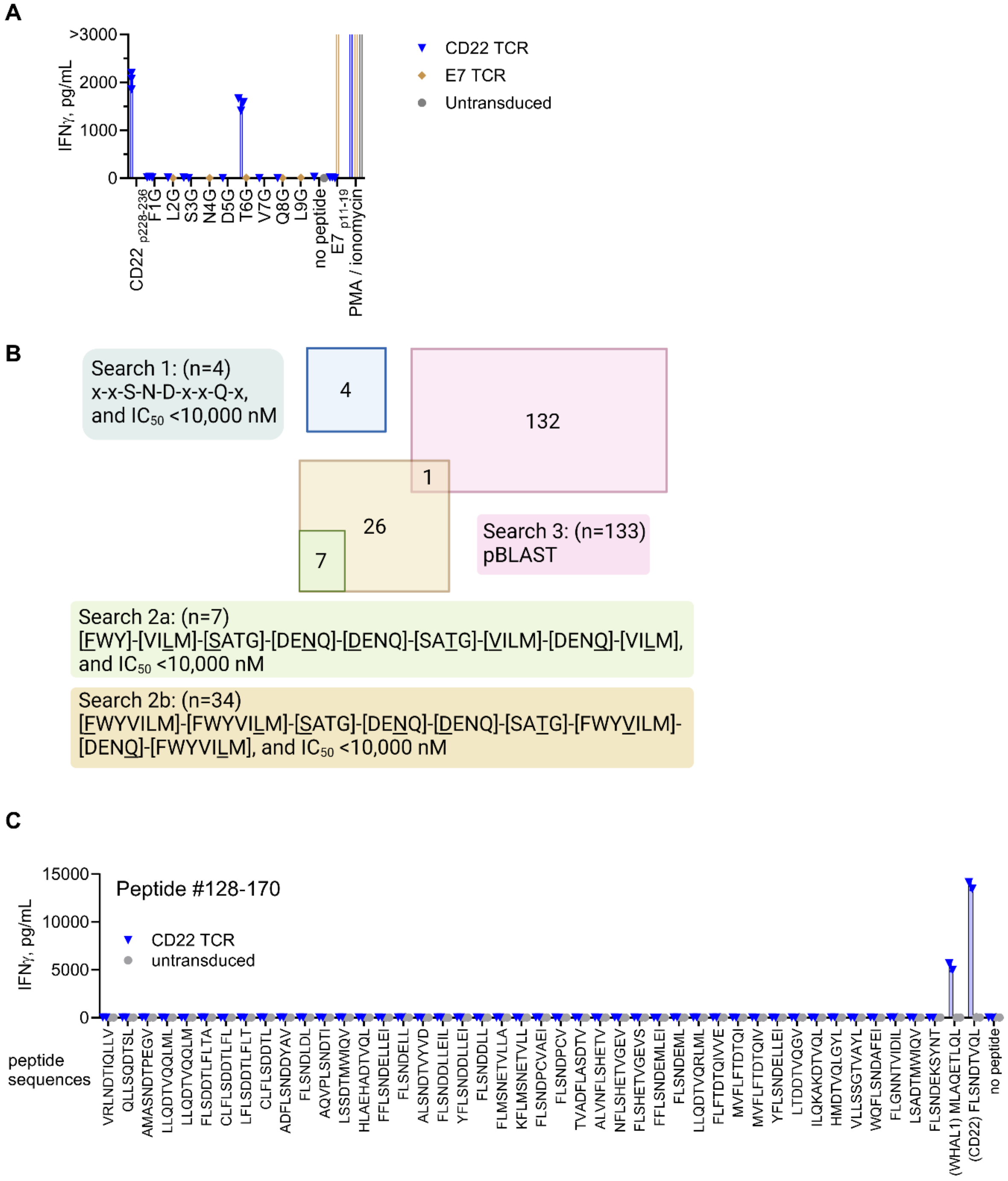

Fig. S4. Cross-reactivity evaluation

(**A**) TCR-transduced T cells were cocultured with K562A2 loaded with the CD22 epitope peptide ($CD22_{p228\text{-}236}$) or altered ligand peptides with glycine substitution of each residue. IFNγ levels in the overnight coculture supernatant were measured by ELISA. (**B**) Venn diagram of candidate peptides for cross-reactivity screening identified through different *in silico* search rules. The Venn

diagram was made with BioRender.com. (**C**) CD22 TCR-T cells were cocultured with K562A2 loaded with cross-reactive screening candidate peptides #128-170 (1 μM). IFNγ levels in the overnight coculture supernatant were measured by ELISA. Positive controls in this assay include the CD22 epitope peptide and WHAL1-derived peptide with an amino acid sequence of "MLAQETLQL", which was one of the candidate peptides found to be weakly cross-reactive with the CD22 TCR in Fig. 3B and 3C. Representative figures from 3 independent experiments, each with two biological replicates. Technical replicates: n=3 (A) and n=2 (C).

Supplementary Figure S5

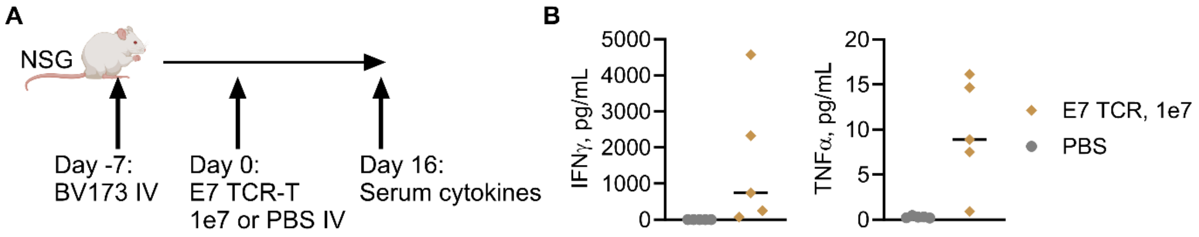

Fig. S5. Xenograft models are limited by subclinical xenogeneic GVHD that starts in less than 3 weeks after T cell infusion

(**A**) NSG mice were intravenously injected with human leukemia cell line BV173 expressing firefly luciferase (BV173-ffLuc) 1e6 cells/mouse on day -7. PBS or HPV-16 E7 TCR-T cells (1e7 cells/mouse) were intravenously administered on day 0. Target leukemia BV173 does not express the E7 antigen. The mouse illustration was made with BioRender.com. (**B**) Serum levels of IFNγ and TNFα were measured with an MSD assay on day 16. Mice that received T cells (irrespective of antigen specificity) exhibited systemic cytokine elevation approximately 2 weeks after T cell infusion even in the absence of overt xeno-GVHD symptoms. The data are consistent with limitations of xenograft models well-described in the field, which include subclinical systemic cytokine elevation related to xeno-GVHD that starts as early as day 14 (126). Biological replicates: n=5 (B).

Supplementary Figure S6

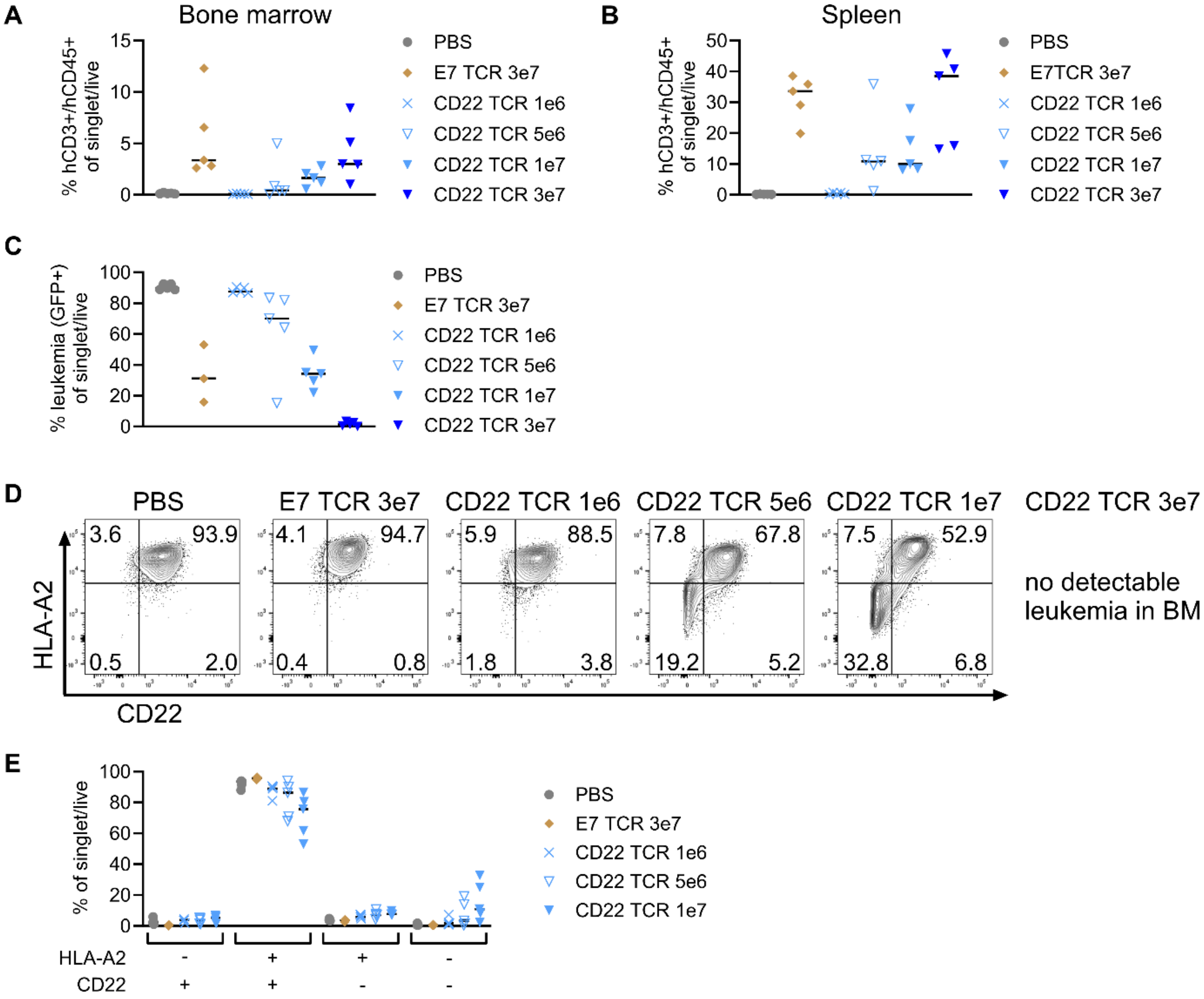

Fig. S6. CD22 and HLA-A2 expressions are lost in a population of leukemia cells that progress after CD22 TCR-T cell infusion

Following the experimental schema shown in Fig. 4E, NSG mice were intravenously injected with human leukemia cell line BV173 expressing firefly luciferase (BV173-ffLuc) 1e6 cells/mouse on day -7, and T cells were intravenously administered on day 0. Mice were sacrificed on day 24, and the frequencies of adoptively transferred T cells in (**A**) bone marrow and (**B**) spleen were assessed. (**C**) Frequencies of leukemia cells detectable in bone marrow on day 24. (**D, E**) Cell-surface HLA-A2 and CD22 expression on leukemia cells were assessed on day 24 with flow cytometry analysis. (D) Representative dot plots from each treatment group and (E) frequencies of leukemia cells with indicated phenotypes are shown. Biological replicates: n=5.

Supplementary Figure S7

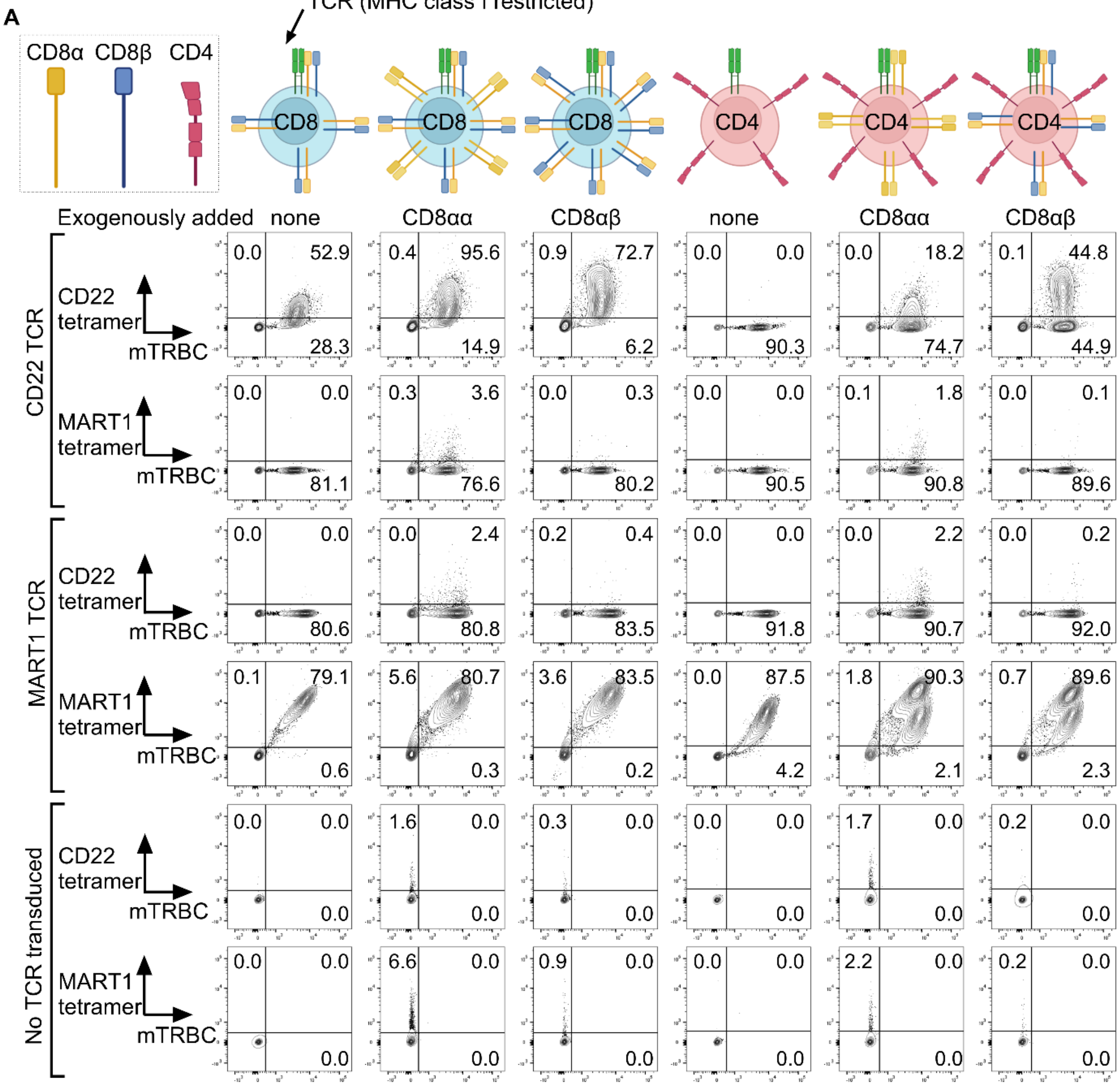

Fig. S7. The CD22 TCR-T co-expressing exCD8αβ demonstrated augmented and specific bindings to the cognate p-MHC tetramers

CD8+ or CD4+ T cells were transduced with either CD22 TCR or MART1 TCR (clone DMF5). Each TCR-T underwent second transduction to express either CD8αα or CD8αβ (the same schematic illustration as the main Fig. 5A). Flow cytometry dot plots show CD22 tetramer-binding and MART1 tetramer-binding of each cell product. Murine TCR beta constant region (mTRBC) is a marker of transduction efficiency. Schematic illustrations were made with BioRender.com.

Supplementary Figure S8

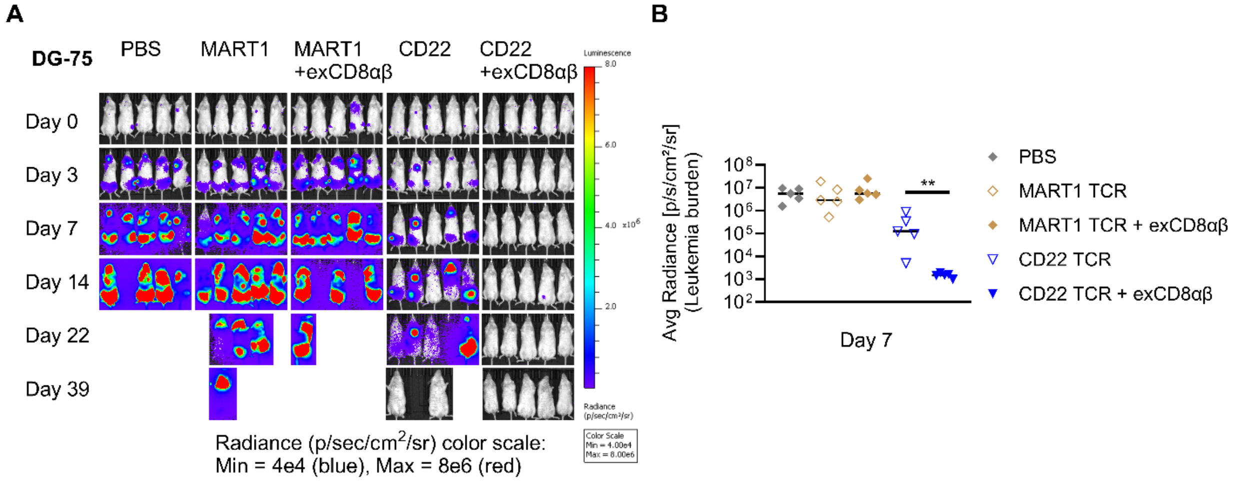

Fig. S8. The CD22 TCR-T co-expressing exCD8αβ heterodimers mediate robust anti-lymphoma *in vivo* activity

NSG mice were intravenously injected with human lymphoma cell line DG-75-ffLuc 1e6 cells/mouse on day -7. On day 0, mice were treated with intravenous infusion of CD22 TCR-T cells or MART1 TCR-T cells, each with or without exCD8αβ, 2e7 cells/mouse. Bioluminescent signals (tumor burden) were measured with IVIS (Perkin Elmer). (**A**) IVIS images and (**B**) average radiance of bioluminescent signals on day 7 are shown. *P < 0.05 and **P < 0.01 by Kruskal-Wallis test with Dunn's correction (B). Representative figures from 2 independent experiments. Biological replicates: n=5.

Supplementary Figure S9

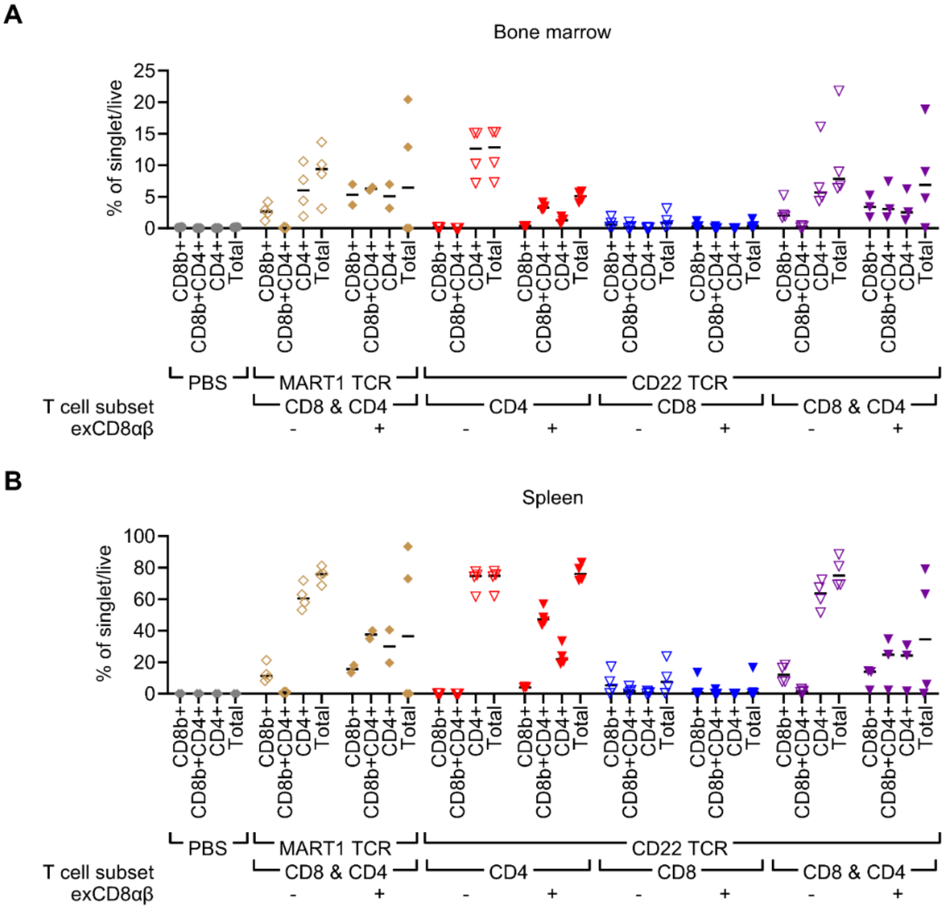

Fig. S9. Frequencies of adoptively transferred TCR-T cells with and without exCD8αβ

Leukemia-engrafted mice were treated as shown in the schema Fig. 5F. Frequencies of adoptively transferred TCR-T cells and their cell-surface CD4 and CD8 co-receptor expression were assessed with flow cytometry on day 30 in (**A**) bone marrow and (**B**) spleen. T cells either expressed only CD8αβ (labeled as CD8b+ in the x-axis), only CD4 (labeled as CD4+ in the x-axis), or both CD8αβ and CD4 (labeled as CD8b+CD4+ in the x-axis). Biological replicates: n=4-5 (except PBS; n=3).

Supplementary Figure S10

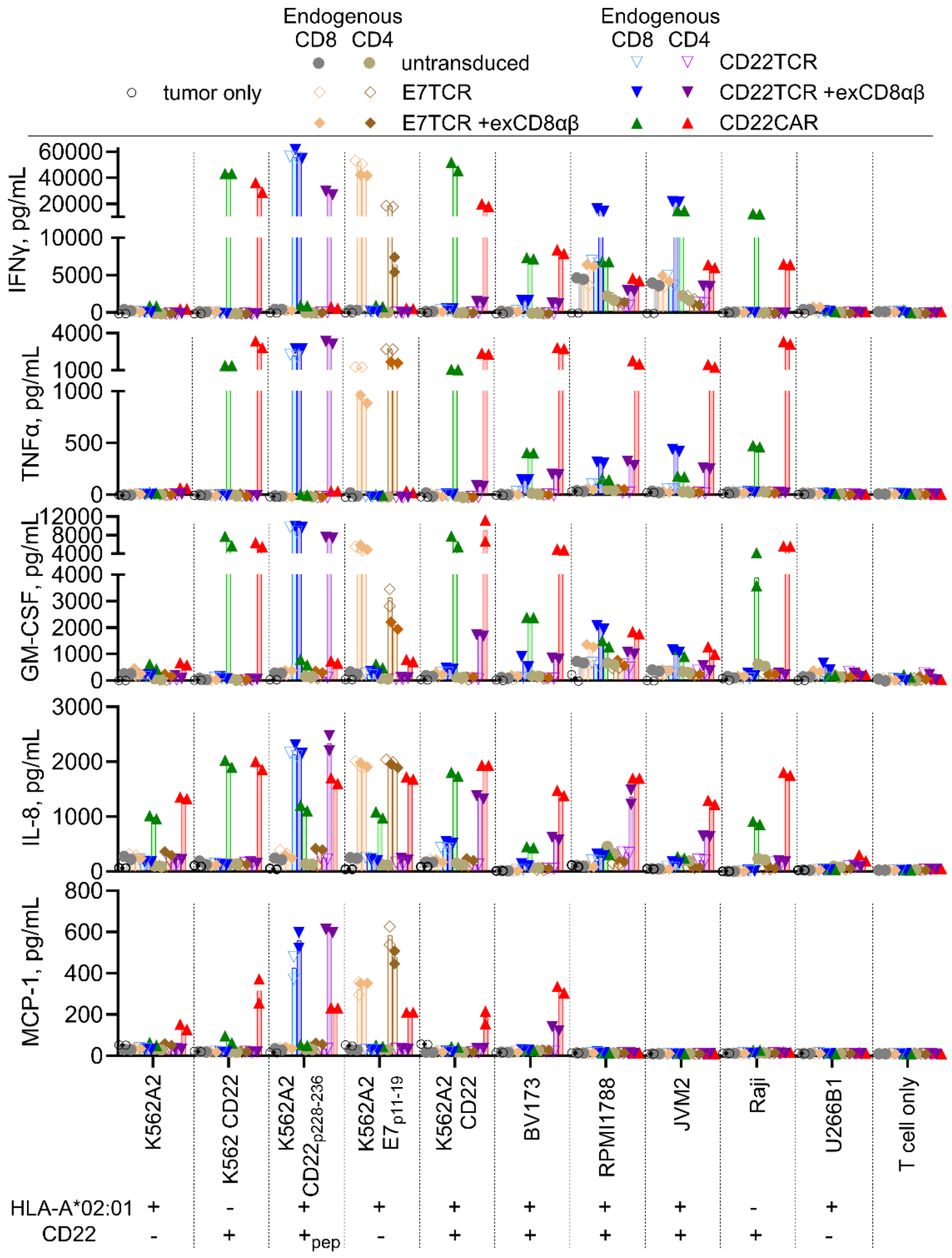

Fig. S10. *In vitro* cytokine production by CD22 CAR-T and CD22 TCR-T with or without exCD8αβ

Each cell product was cocultured with the indicated target cells at an E:T = 1:1 (5e4 cells each/well in 96-well U-bottom plate). Levels of IFNγ, TNFα, GM-CSF, IL-8, and MCP-1 in overnight coculture supernatant were measured by ELISA. The same dataset is shown in a heatmap format in Fig. 6C. Technical duplicate.

Supplementary Figure S11

A

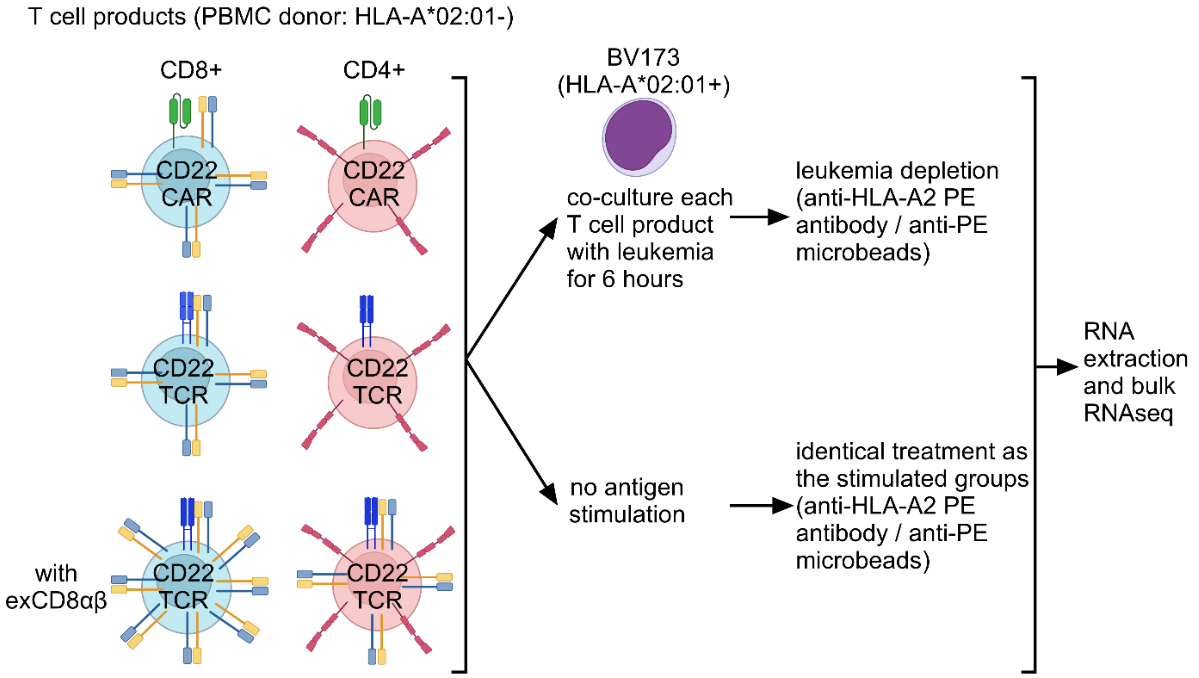

B

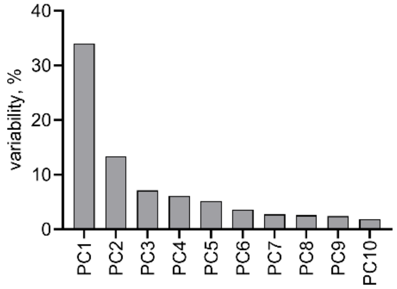

Fig. S11. CD22 CAR-T and CD22 TCR-T cells have distinct transcriptional states before and after antigen encounter

(**A**) Bulk RNAseq experiment design. Either CD8+ or CD4+ T cells were transduced with CD22 CAR, CD22 TCR, or CD22 TCR + exCD8αβ. After the completion of transduction, T cells were rested in culture in the presence of recombinant human IL-2 (200 IU/mL) for 1 week. Then T cells were cocultured with leukemia cells (BV173) for 6 hours at an E:T = 2:1. Upon completion of coculture, residual leukemia cells were depleted with magnetic isolation (anti-HLA-A2 PE antibody and anti-PE microbeads). T cells that were not stimulated with BV173 were treated equally as the stimulated groups and underwent the identical depletion step. Enriched T cells were snap frozen (a small portion of enriched T cells were set aside and assessed for T-cell purity using flow cytometry analysis). Snap-frozen cell pellets were thawed once immediately prior to RNA extraction, which was performed in a single batch. Experiment was performed with 3 biological replicates. Schematic illustrations were made with BioRender.com. (**B**) Principal component analysis of bulk RNAseq data was performed. The plot shows the variability accounted by top ten principal components (PCs) out of a total of 36 PCs.

Supplementary Figure S12

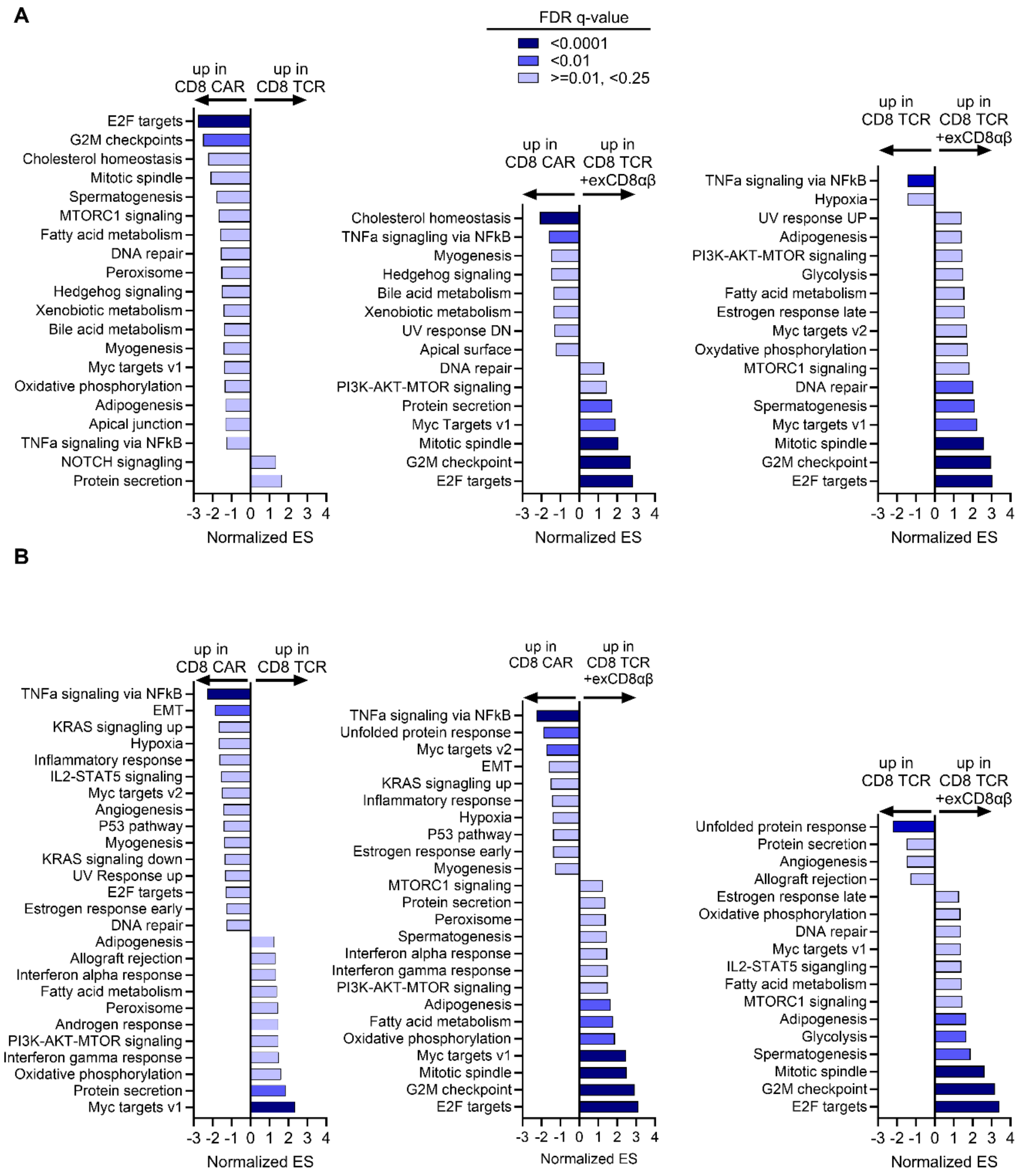

Fig. S12. GSEA of RNAseq data for contrasts among CD8+ T cell products before and after stimulation by CD22+ leukemia cells

Gene sets (Hallmark) that were significantly enriched in either direction with FDR q-value <0.25 and adjusted p-value <0.05 are shown. Analyses (**A**) before stimulation and (**B**) after stimulation with BV173 are shown. The middle graph of the S12B (the contrast between antigen-stimulated CD8 CAR vs. CD8 TCR +exCD8αβ) is identical to the Fig. 6E but shown here again for the ease of visual comparison with other graphs.

Supplementary Figure S13

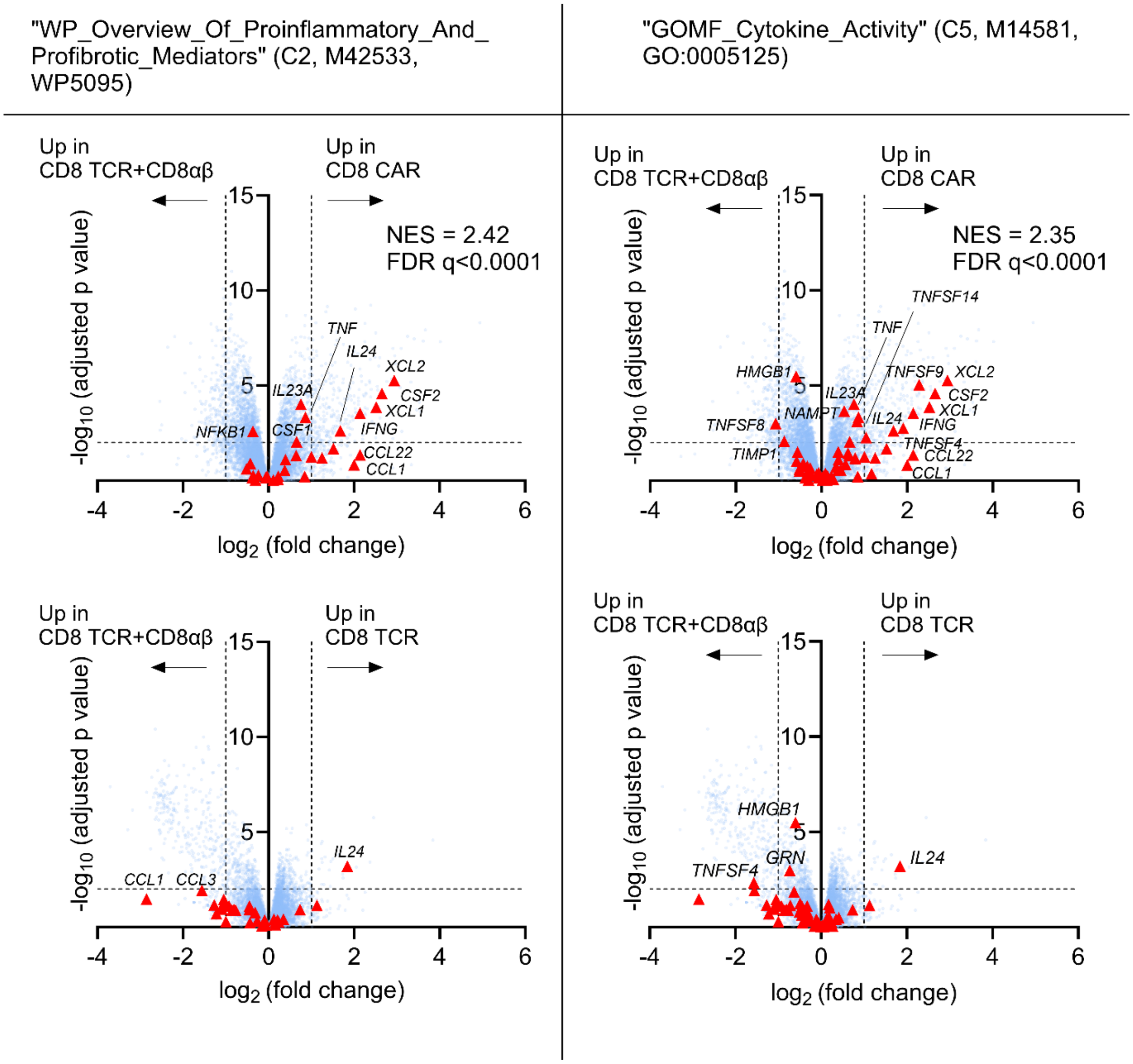

Fig. S13. Proinflammatory gene transcripts and cytokine/chemokine transcripts are upregulated in CD22 CAR-T cells after antigen stimulation

Volcano plots show gene expression data comparing CD8+ CAR-T cells and CD8+ TCR-T + exCD8αβ (top row), and CD8 TCR-T and CD8 TCR-T + exCD8αβ (bottom row) ([–log10(P value) vs. log2(fold change)]). Red triangle symbols are genes that belong to the human "C2 curated gene sets, WP_OVERVIEW_OF_PROINFLAMMATORY_AND_PROFIBROTIC_MEDIATORS (M42533)" (left column) and "C5 gene ontology gene sets, C5, GOMF_CYTOKINE_ACTIVITY (M14581)" (right column).

Supplementary Figure S14

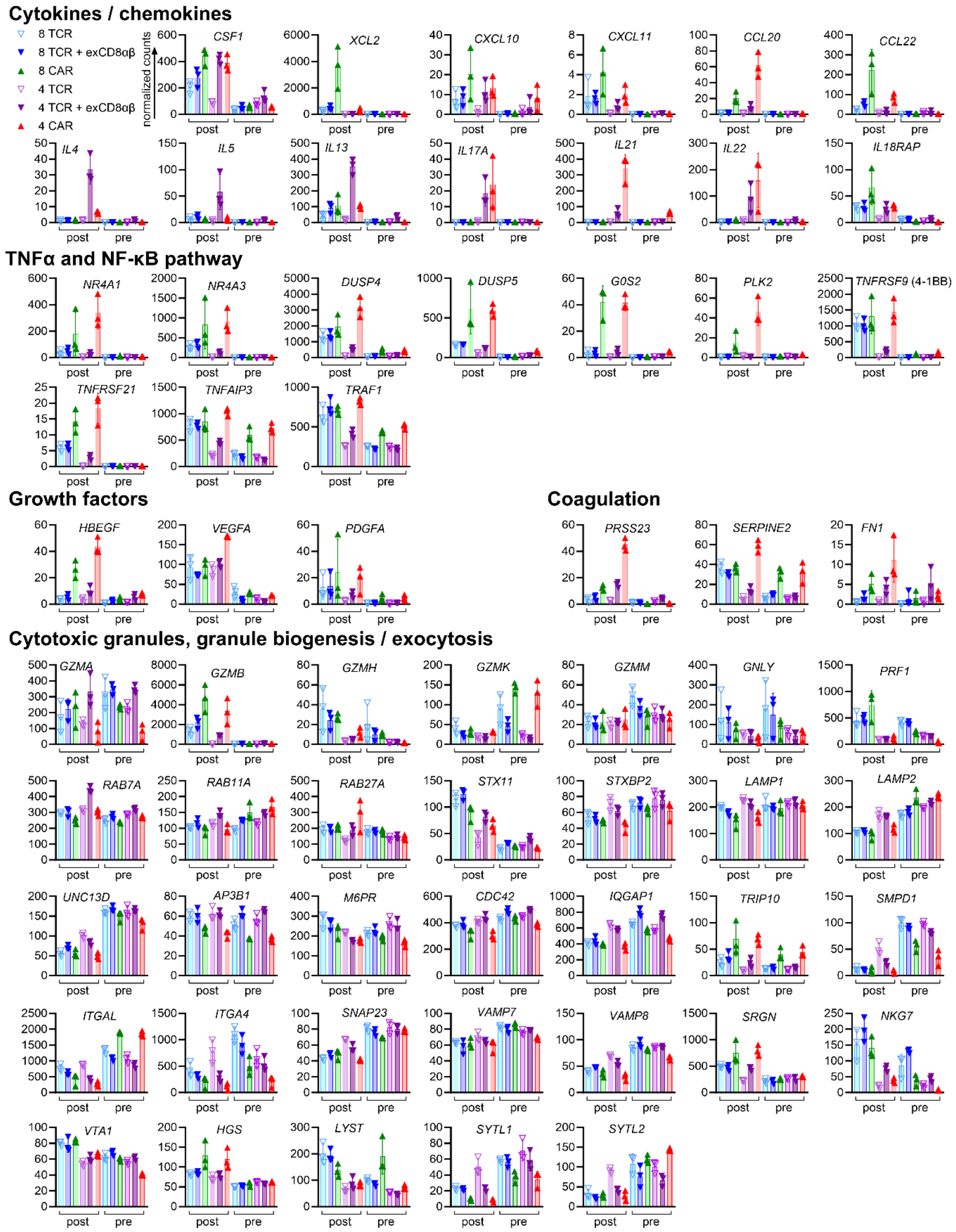

Fig. S14. Transcripts of CD22 CAR-T, CD22 TCR-T, and CD22 TCR-T + exCD8αβ before and after *in vitro* antigen stimulation

RNA normalized counts of select genes are shown for CD8+ (8) and CD4+ (4) T cell products pre- and 6 hours post-stimulation with CD22+ leukemia cells.

Supplementary Figure S15

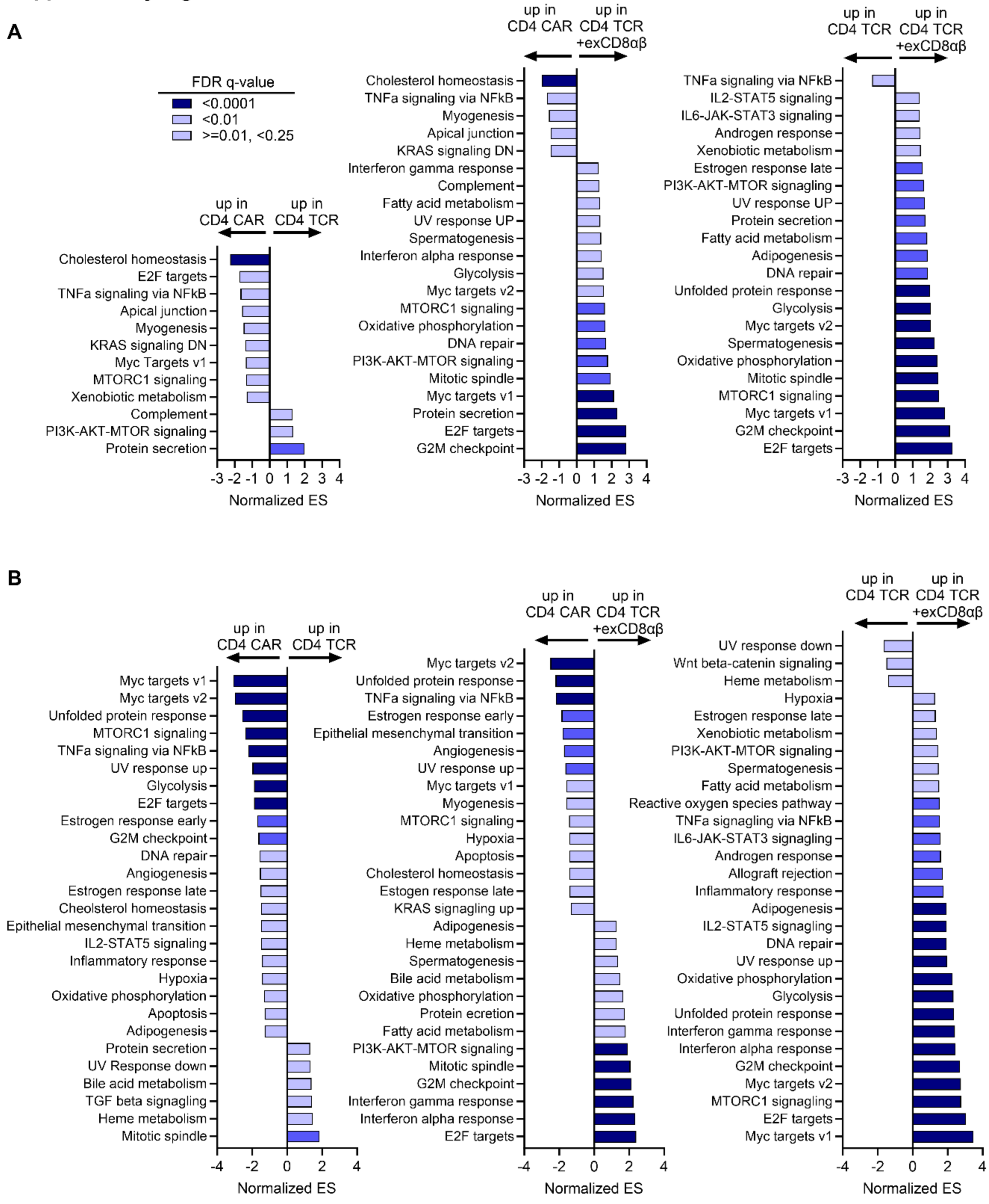

Fig. S15. GSEA of RNAseq data for contrasts among CD4+ T cell products before and after stimulation by CD22+ leukemia cells

Gene sets (Hallmark) that were significantly enriched in either direction with FDR q-value <0.25 and adjusted p-value <0.05 are shown. Contrasts among pre-stimulation (**A**) and post-stimulation (**B**) samples are shown.

Supplementary Figure S16

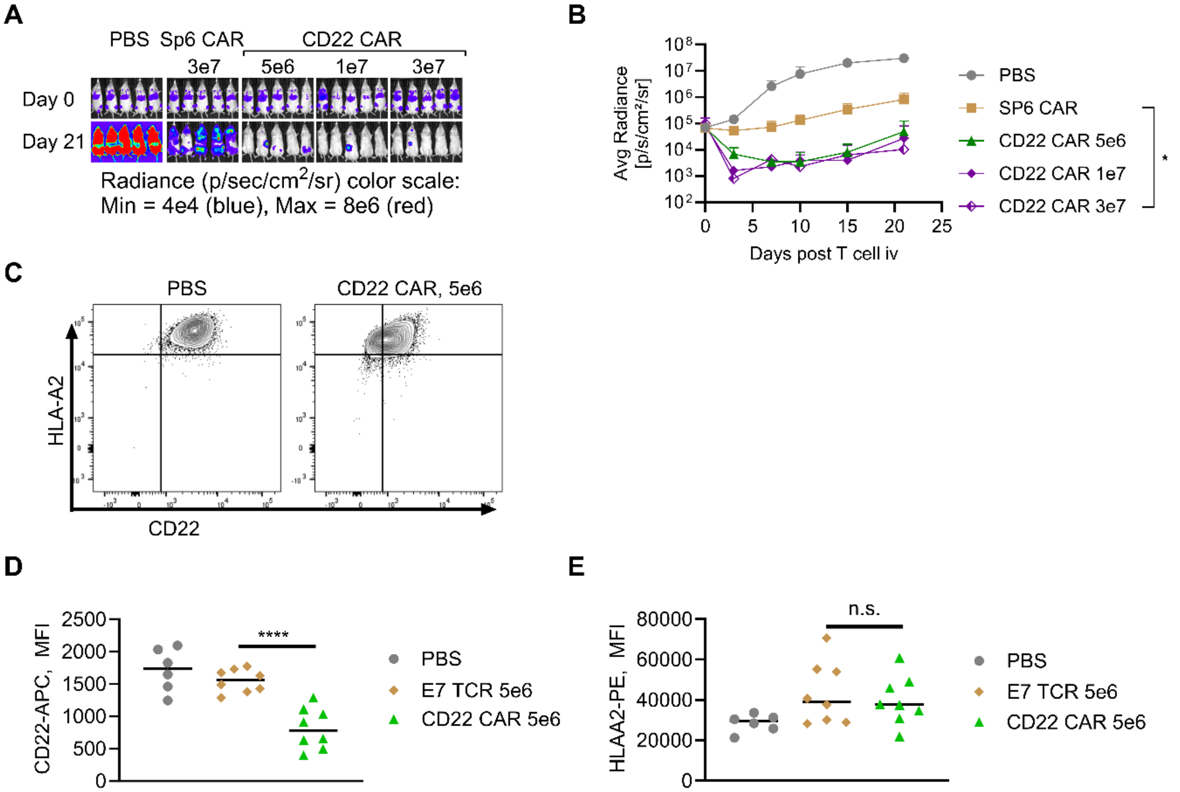

Fig. S16. CD22 CAR-T cells eradicate leukemia in murine xenograft model

(**A, B**) Using the experimental schema in Fig. 4E, leukemia-engrafted mice were treated with CD22 CAR-T cells 5e6, 1e7, or 3e7 cells/mouse. Sp6 CAR used as a negative control recognizes the hapten 2,4,6-trinitrophenyl (127). Bioluminescent signals were measured by IVIS, and (**A**) images and (**B**) average radiance are shown. (**C-E**) Using the experimental schema in Fig. 4E, leukemia-engrafted mice were treated with CD22 CAR-T cells or E7 TCR-T cells (5e6 cells/mouse). Mice were sacrificed on day 30 and cell-surface HLA-A2 and CD22 expression on leukemia cells (in bone marrow) were assessed with flow cytometry. (**C**) Representative dot plots and mean fluorescent intensity (MFI) of (**D**) CD22-APC (CD22 stained with APC-conjugated anti-CD22 antibody) and (**E**) HLA-A2-PE (HLA-A2 stained with PE-conjugated anti-HLA-A2 antibody) are shown. Biological replicates: n=5 (A, B), n=8 (E7 TCR-T, CD22 CAR-T) and n=6 (PBS) (D, E).

Supplementary Figure S17

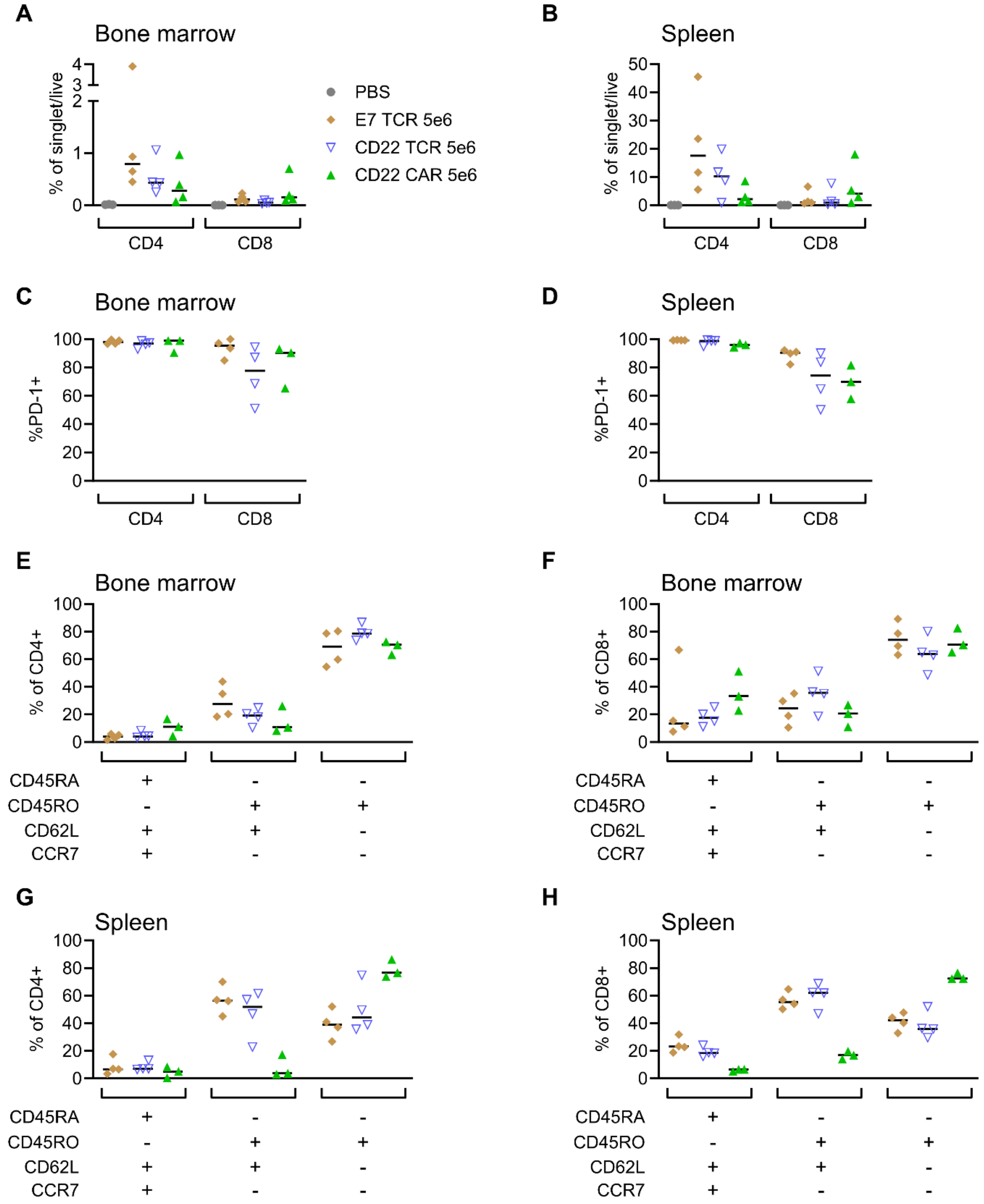

Fig. S17. Frequencies and phenotype of adoptively transferred T cells detectable on day 30

Using the experimental schema in Fig. 4E, Leukemia-bearing mice were treated with 5e6 cells/mouse of E7 TCR-T, CD22 TCR-T, or CD22 CAR-T cells. Frequencies and phenotype of adoptively transferred T cells were assessed on day 30 in bone marrow (A, C, E, F) and spleens (B, D, G, H). Of note, it is challenging to make a biological inference about these T-cell phenotype data at a late timepoint in xenograft model due to known confounding effects by xenogeneic reactions, which have been known to affect *in vivo* T cell frequencies and phenotypes (128).

Supplementary Figure S18

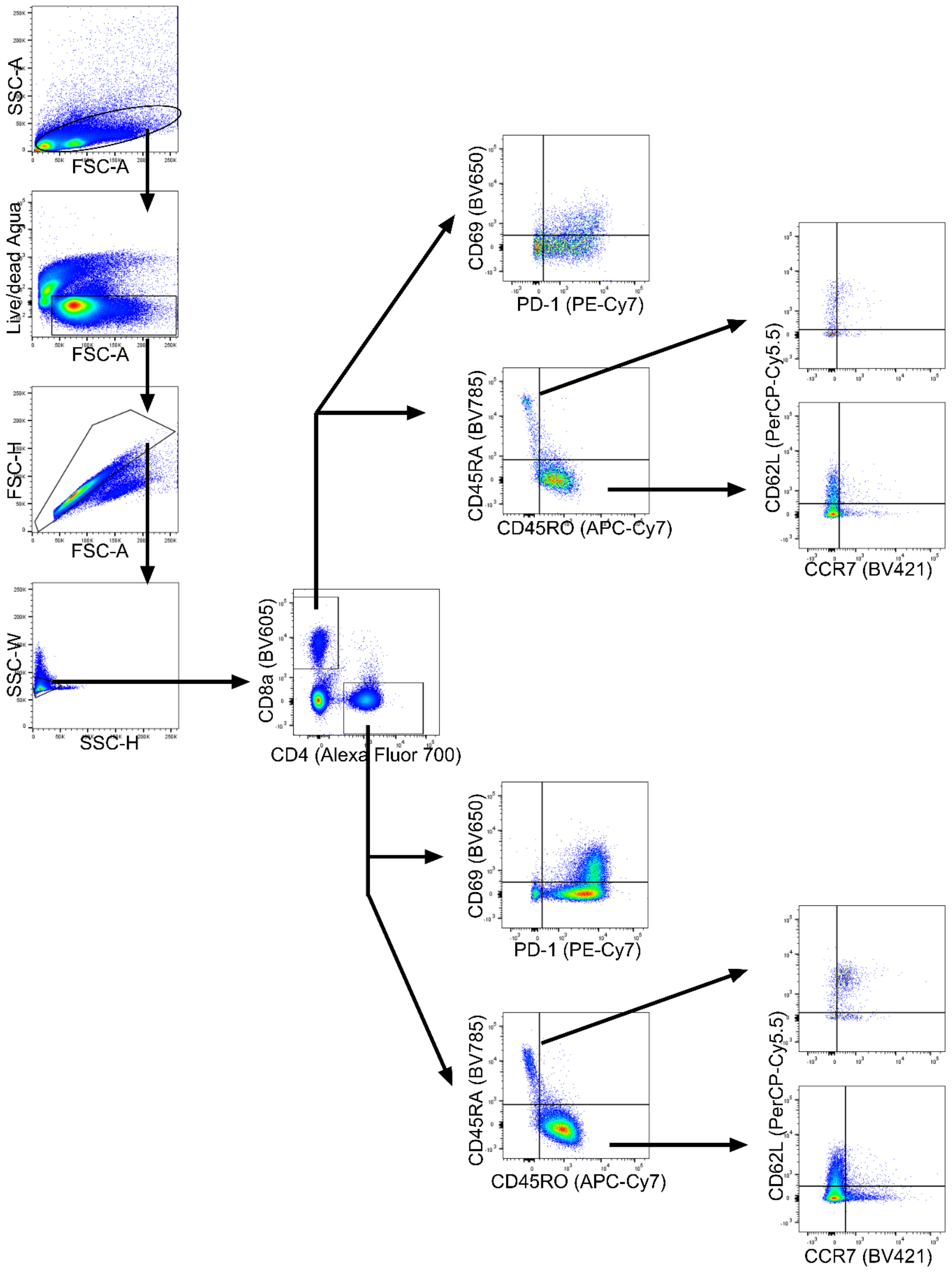

Fig. S18. Flow cytometry gating strategy for T cell phenotype assessment shown in Fig. S17

Supplementary Figure S19

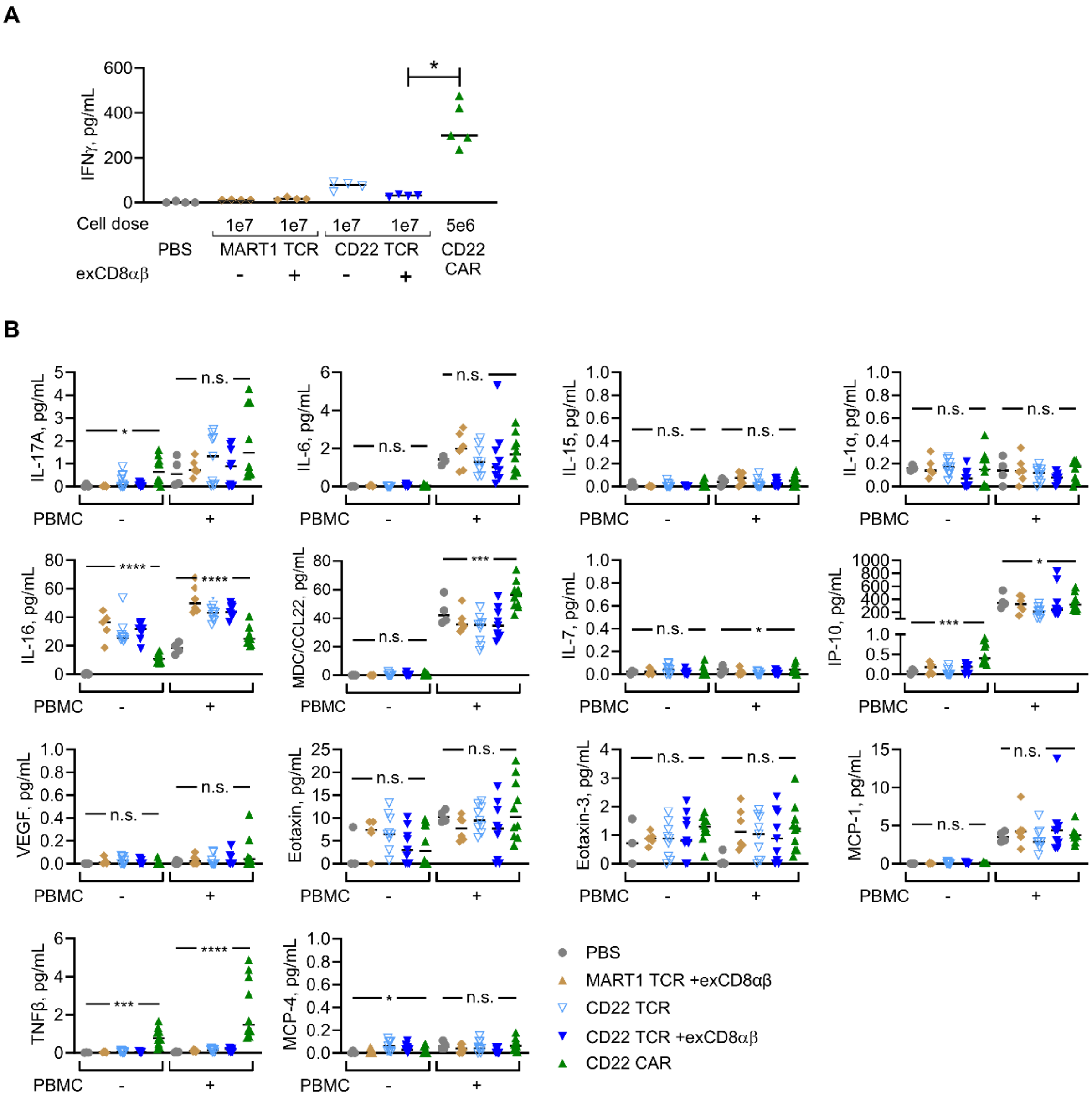

Fig. S19. The CD22 CAR-T cell treatment increases the levels of circulating pro-inflammatory cytokines

In vivo experiments were executed as described in Fig. 4E (for Fig. S19A) and Fig. 7F (for Fig. S19B). Serum cytokine levels measured 2 days after T cell infusion using Meso Scale Discovery assay. (A) Representative of 3 independent experiments. Biological replicates: n=4, except for n=5 in CD22 TCR 3e7 and CD22 CAR 5e6 groups. (B) Pooled data from 2 independent experiments with total biological replicates: n=10 (CD22 CAR, CD22 CAR +PBMC), n=9 (CD22 TCR, CD22 TCR + PBMC, CD22 TCR+exCD8αβ, CD22 TCR+exCD8αβ +PBMC), n=6 (MART1 TCR+ exCD8αβ +PBMC), n=5 (MART1 TCR+exCD8αβ), n=4 (PBS +PBMC), n=3 (PBS). *P < 0.05 **P < 0.01, ***P<0.001, and ****P<0.0001 by Kruskal-Wallis test with Dunn’s correction.

Supplementary Figure S20

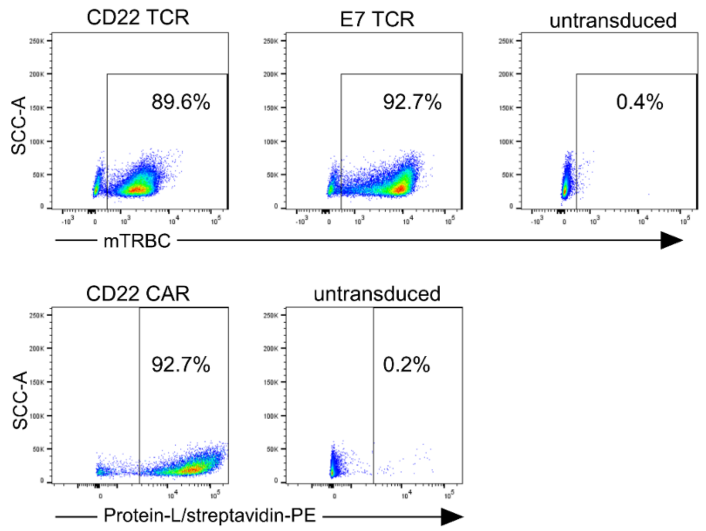

Fig. S20. Transduction efficiency of receptor-transduced T cells

Flow cytometry dot plot shows transduction efficiency of T cells transduced with MSGV1-based vector encoding indicated receptors.

Supplementary Figure S21

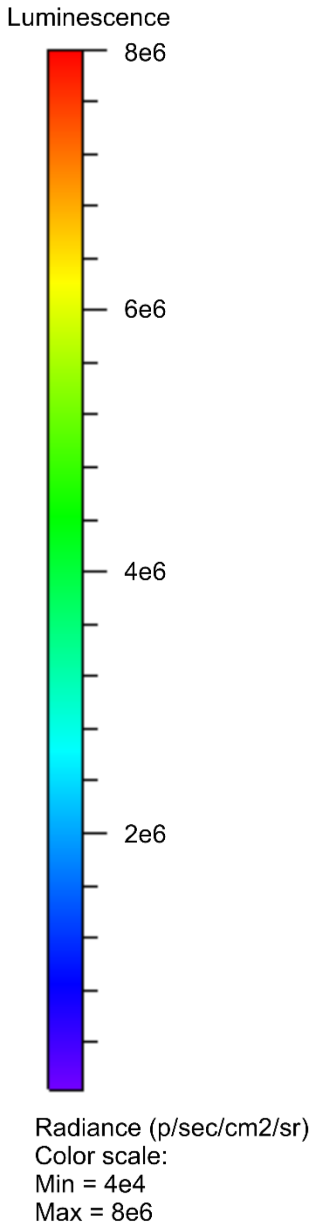

Fig. S21. Color scale of IVIS bioluminescence imaging data

The example shows the radiance (p/sec/cm2/sr) in the color scale ranging from the minimum of 4e4 (blue) to the maximum of 8e6 (red).

Supplementary Table S1: Paired TCR alpha and beta chain CDR3 sequences of the CD22 tetramer-binding T cell population in donor 146 well #8

TCR chain	Allele name	CDR3 amino acid sequence
Alpha	TRAV12-2*01, TRAJ9*01	CAVKGAGGFKTIF
Beta	TRBV7-9*01, TRBJ1-1*01, TRBD1*01	CASSPGNTEAFF

Supplementary Table S2: Predicted HLA-A*02:01 IC_{50} of $CD22_{p228\text{-}236}$ epitope and alanine- and glycine-substituted peptides

Peptide amino acid sequences	IC_{50} (nM)
FLSNDTVQL ($CD22_{p228\text{-}236}$)	5.8
**A**LSNDTVQL	80.7
F**A**SNDTVQL	1974.1
FL**A**NDTVQL	4.4
FLS**A**DTVQL	5.1
FLSN**A**TVQL	5.3
FLSND**A**VQL	10.2
FLSNDT**A**QL	6.8
FLSNDTV**A**L	5.5
FLSNDTVQ**A**	26.8
**G**LSNDTVQL	129.5
F**G**SNDTVQL	8459.1
FL**G**NDTVQL	9.6
FLS**G**DTVQL	7
FLSN**G**TVQL	5.5
FLSND**G**VQL	21.6
FLSNDT**G**QL	43.1
FLSNDTV**G**L	5.5
FLSNDTVQ**G**	3062.6

Supplementary Table S3: Antibodies and reagents used for flow cytometry analysis

Target antigen	Clone	Conjugate	Company	Catalog #
TCRβ (TRBC), mouse	H57-597	FITC	Invitrogen / eBioscience	11-5961-85
TCRβ (TRBC), mouse	H57-597	PE	Invitrogen / eBioscience	12-5961-83
TCRβ (TRBC), mouse	H57-597	APC-Cy7	BioLegend	109220
CD19, human	SJ25C1	eFluor 450	Invitrogen / eBioscience	48-0198-42
CD22, human	S-HCL-1	APC	BioLegend	363506
CD20, human	2H7	FITC	BioLegend	302304
CD3, human	SK7	PerCP-Cy5.5	BioLegend	344808
CD8a, human	SK1	eFluor 450	Invitrogen / eBioscience	48-0087-42
CD8a, human	RPA-T8	PerCP	BioLegend	301030
CD8a, human	RPA-T8	BV421	BioLegend	301036
CD8a, human	HIT8a	BV605	BioLegend	300936
CD8b, human	QA20A40	APC	BioLegend	376706
CD4, human	RPA-T4	APC-eFluor 780	Invitrogen / eBioscience	47-0049-42
CD4, human	RPA-T4	BV421	BioLegend	300532
CD4, human	RPA-T4	Alexa Fluor 700	BioLegend	344622
CD45, human	HI30	PE-Cy7	BioLegend	304016
CD107a	H4A3	PerCP-Cy5.5	BioLegend	328616
IFNγ, human	4S.B3	PE-Cy7	Invitrogen / eBioscience	25-7319-82
IL-2, human	MQ1-17H12	APC	Invitrogen / eBioscience	17-7029-82
TNFα, human	MAb11	PE	Invitrogen / eBioscience	12-7349-82
HLA-A2	BB7.2	PE-Cy7	BioLegend	343314
HLA-A2	BB7.2	PE	BioLegend	343306
PD-1, human	EH12.2H7	PE-Cy7	BioLegend	329918
CD69, human	FN50	BV650	BioLegend	310934
CD45RA, human	HI100	BV785	BioLegend	304140
CD45RO, human	UCHL1	APC-Cy7	BioLegend	304228
CD62L, human	DREG-56	PerCP-Cy5.5	BioLegend	304824
CCR7, human	G043H7	BV421	BioLegend	353208
Streptavidin-PE conjugate	N/A	PE	Invitrogen / eBioscience	12-4317-87
Streptavidin-APC conjugate	N/A	APC	Invitrogen / eBioscience	17-4317-82
Viability dye (fixable)	N/A	eFluor 506	Invitrogen / eBioscience	65-0866-14

7AAD (BD Via-Probe)	N/A	N/A	BD Biosciences	555815
Live/Dead Fixable Aqua	N/A	405 nm excitation	Invitrogen	L34966
CellTrace Far Red Cell Proliferation Kit	N/A	630 nm excitation	Invitrogen	C34564
CellTrace Yellow Cell Proliferation Kit	N/A	546 nm excitation	Invitrogen	C34567
Flex-T HLA-A*02:01 Monomer UVX	N/A	N/A	BioLegend	280004

Supplementary Datasheet S1 (separate file)

Clinical cell doses of representative TCR-T cells and CAR-T cells in clinic

REFERENCES AND NOTES

1. J. N. Kochenderfer, M. E. Dudley, R. O. Carpenter, S. H. Kassim, J. J. Rose, W. G. Telford, F. T. Hakim, D. C. Halverson, D. H. Fowler, N. M. Hardy, A. R. Mato, D. D. Hickstein, J. C. Gea-Banacloche, S. Z. Pavletic, C. Sportes, I. Maric, S. A. Feldman, B. G. Hansen, J. S. Wilder, B. Blacklock-Schuver, B. Jena, M. R. Bishop, R. E. Gress, S. A. Rosenberg, Donor-derived CD19-targeted T cells cause regression of malignancy persisting after allogeneic hematopoietic stem cell transplantation. *Blood* **122**, 4129–4139 (2013).

2. S. L. Maude, N. Frey, P. A. Shaw, R. Aplenc, D. M. Barrett, N. J. Bunin, A. Chew, V. E. Gonzalez, Z. Zheng, S. F. Lacey, Y. D. Mahnke, J. J. Melenhorst, S. R. Rheingold, A. Shen, D. T. Teachey, B. L. Levine, C. H. June, D. L. Porter, S. A. Grupp, Chimeric antigen receptor T cells for sustained remissions in leukemia. *N. Engl. J. Med.* **371**, 1507–1517 (2014).

3. D. W. Lee, J. N. Kochenderfer, M. Stetler-Stevenson, Y. K. Cui, C. Delbrook, S. A. Feldman, T. J. Fry, R. Orentas, M. Sabatino, N. N. Shah, S. M. Steinberg, D. Stroncek, N. Tschernia, C. Yuan, H. Zhang, L. Zhang, S. A. Rosenberg, A. S. Wayne, C. L. Mackall, T cells expressing CD19 chimeric antigen receptors for acute lymphoblastic leukaemia in children and young adults: A phase 1 dose-escalation trial. *Lancet* **385**, 517–528 (2015).

4. C. J. Turtle, L. A. Hanafi, C. Berger, T. A. Gooley, S. Cherian, M. Hudecek, D. Sommermeyer, K. Melville, B. Pender, T. M. Budiarto, E. Robinson, N. N. Steevens, C. Chaney, L. Soma, X. Chen, C. Yeung, B. Wood, D. Li, J. Cao, S. Heimfeld, M. C. Jensen, S. R. Riddell, D. G. Maloney, CD19 CAR-T cells of defined $CD4^+$:$CD8^+$ composition in adult B cell ALL patients. *J. Clin. Invest.* **126**, 2123–2138 (2016).

5. R. A. Gardner, O. Finney, C. Annesley, H. Brakke, C. Summers, K. Leger, M. Bleakley, C. Brown, S. Mgebroff, K. S. Kelly-Spratt, V. Hoglund, C. Lindgren, A. P. Oron, D. Li, S. R. Riddell, J. R. Park, M. C. Jensen, Intent-to-treat leukemia remission by CD19 CAR T cells of defined formulation and dose in children and young adults. *Blood* **129**, 3322–3331 (2017).

6. S. S. Neelapu, F. L. Locke, N. L. Bartlett, L. J. Lekakis, D. B. Miklos, C. A. Jacobson, I. Braunschweig, O. O. Oluwole, T. Siddiqi, Y. Lin, J. M. Timmerman, P. J. Stiff, J. W. Friedberg,

I. W. Flinn, A. Goy, B. T. Hill, M. R. Smith, A. Deol, U. Farooq, P. McSweeney, J. Munoz, I. Avivi, J. E. Castro, J. R. Westin, J. C. Chavez, A. Ghobadi, K. V. Komanduri, R. Levy, E. D. Jacobsen, T. E. Witzig, P. Reagan, A. Bot, J. Rossi, L. Navale, Y. Jiang, J. Aycock, M. Elias, D. Chang, J. Wiezorek, W. Y. Go, Axicabtagene ciloleucel CAR T-cell therapy in refractory large B-cell lymphoma. *N. Engl. J. Med.* **377**, 2531–2544 (2017).

7. S. L. Maude, T. W. Laetsch, J. Buechner, S. Rives, M. Boyer, H. Bittencourt, P. Bader, M. R. Verneris, H. E. Stefanski, G. D. Myers, M. Qayed, B. De Moerloose, H. Hiramatsu, K. Schlis, K. L. Davis, P. L. Martin, E. R. Nemecek, G. A. Yanik, C. Peters, A. Baruchel, N. Boissel, F. Mechinaud, A. Balduzzi, J. Krueger, C. H. June, B. L. Levine, P. Wood, T. Taran, M. Leung, K. T. Mueller, Y. Zhang, K. Sen, D. Lebwohl, M. A. Pulsipher, S. A. Grupp, Tisagenlecleucel in children and young adults with B-cell lymphoblastic leukemia. *N. Engl. J. Med.* **378**, 439–448 (2018).

8. J. H. Park, I. Riviere, M. Gonen, X. Wang, B. Senechal, K. J. Curran, C. Sauter, Y. Wang, B. Santomasso, E. Mead, M. Roshal, P. Maslak, M. Davila, R. J. Brentjens, M. Sadelain, Long-term follow-up of CD19 CAR therapy in acute lymphoblastic leukemia. *N. Engl. J. Med.* **378**, 449–459 (2018).

9. S. J. Schuster, M. R. Bishop, C. S. Tam, E. K. Waller, P. Borchmann, J. P. McGuirk, U. Jager, S. Jaglowski, C. Andreadis, J. R. Westin, I. Fleury, V. Bachanova, S. R. Foley, P. J. Ho, S. Mielke, J. M. Magenau, H. Holte, S. Pantano, L. B. Pacaud, R. Awasthi, J. Chu, O. Anak, G. Salles, R. T. Maziarz, J. Investigators, Tisagenlecleucel in adult relapsed or refractory diffuse large B-cell lymphoma. *N. Engl. J. Med.* **380**, 45–56 (2019).

10. T. J. Fry, N. N. Shah, R. J. Orentas, M. Stetler-Stevenson, C. M. Yuan, S. Ramakrishna, P. Wolters, S. Martin, C. Delbrook, B. Yates, H. Shalabi, T. J. Fountaine, J. F. Shern, R. G. Majzner, D. F. Stroncek, M. Sabatino, Y. Feng, D. S. Dimitrov, L. Zhang, S. Nguyen, H. Qin, B. Dropulic, D. W. Lee, C. L. Mackall, CD22-targeted CAR T cells induce remission in B-ALL that is naive or resistant to CD19-targeted CAR immunotherapy. *Nat. Med.* **24**, 20–28 (2018).

11. N. Raje, J. Berdeja, Y. Lin, D. Siegel, S. Jagannath, D. Madduri, M. Liedtke, J. Rosenblatt, M. V. Maus, A. Turka, L. P. Lam, R. A. Morgan, K. Friedman, M. Massaro, J. Wang, G. Russotti,

Z. Yang, T. Campbell, K. Hege, F. Petrocca, M. T. Quigley, N. Munshi, J. N. Kochenderfer, Anti-BCMA CAR T-cell therapy bb2121 in relapsed or refractory multiple myeloma. *N. Engl. J. Med.* **380**, 1726–1737 (2019).

12. S. S. Neelapu, S. Tummala, P. Kebriaei, W. Wierda, C. Gutierrez, F. L. Locke, K. V. Komanduri, Y. Lin, N. Jain, N. Daver, J. Westin, A. M. Gulbis, M. E. Loghin, J. F. de Groot, S. Adkins, S. E. Davis, K. Rezvani, P. Hwu, E. J. Shpall, Chimeric antigen receptor T-cell therapy—Assessment and management of toxicities. *Nat. Rev. Clin. Oncol.* **15**, 47–62 (2018).

13. E. C. Morris, S. S. Neelapu, T. Giavridis, M. Sadelain, Cytokine release syndrome and associated neurotoxicity in cancer immunotherapy. *Nat. Rev. Immunol.* **22**, 85 –96 (2022).

14. D. T. Teachey, S. F. Lacey, P. A. Shaw, J. J. Melenhorst, S. L. Maude, N. Frey, E. Pequignot, V. E. Gonzalez, F. Chen, J. Finklestein, D. M. Barrett, S. L. Weiss, J. C. Fitzgerald, R. A. Berg, R. Aplenc, C. Callahan, S. R. Rheingold, Z. Zheng, S. Rose-John, J. C. White, F. Nazimuddin, G. Wertheim, B. L. Levine, C. H. June, D. L. Porter, S. A. Grupp, Identification of predictive biomarkers for cytokine release syndrome after chimeric antigen receptor T-cell therapy for acute lymphoblastic leukemia. *Cancer Discov.* **6**, 664–679 (2016).

15. K. A. Hay, L. A. Hanafi, D. Li, J. Gust, W. C. Liles, M. M. Wurfel, J. A. Lopez, J. Chen, D. Chung, S. Harju-Baker, S. Cherian, X. Chen, S. R. Riddell, D. G. Maloney, C. J. Turtle, Kinetics and biomarkers of severe cytokine release syndrome after CD19 chimeric antigen receptor-modified T-cell therapy. *Blood* **130**, 2295–2306 (2017).

16. J. Gust, K. A. Hay, L. A. Hanafi, D. Li, D. Myerson, L. F. Gonzalez-Cuyar, C. Yeung, W. C. Liles, M. Wurfel, J. A. Lopez, J. Chen, D. Chung, S. Harju-Baker, T. Ozpolat, K. R. Fink, S. R. Riddell, D. G. Maloney, C. J. Turtle, Endothelial activation and blood-brain barrier disruption in neurotoxicity after adoptive immunotherapy with CD19 CAR-T cells. *Cancer Discov.* **7**, 1404–1419 (2017).

17. N. N. Shah, S. L. Highfill, H. Shalabi, B. Yates, J. Jin, P. L. Wolters, A. Ombrello, S. M. Steinberg, S. Martin, C. Delbrook, L. Hoffman, L. Little, A. Ponduri, H. Qin, H. Qureshi, A. Dulau-Florea, D. Salem, H. W. Wang, C. Yuan, M. Stetler-Stevenson, S. Panch, M. Tran, C. L.

Mackall, D. F. Stroncek, T. J. Fry, CD4/CD8 T-cell selection affects chimeric antigen receptor (CAR) T-cell potency and toxicity: Updated results from a phase I anti-CD22 CAR T-cell trial. *J. Clin. Oncol.* **38**, 1938–1950 (2020).

18. S. Mailankody, S. M. Devlin, J. Landa, K. Nath, C. Diamonte, E. J. Carstens, D. Russo, R. Auclair, L. Fitzgerald, B. Cadzin, X. Wang, D. Sikder, B. Senechal, V. P. Bermudez, T. J. Purdon, K. Hosszu, D. P. McAvoy, T. Farzana, E. Mead, J. A. Wilcox, B. D. Santomasso, G. L. Shah, U. A. Shah, N. Korde, A. Lesokhin, C. R. Tan, M. Hultcrantz, H. Hassoun, M. Roshal, F. Sen, A. Dogan, O. Landgren, S. A. Giralt, J. H. Park, S. Z. Usmani, I. Riviere, R. J. Brentjens, E. L. Smith, GPRC5D-targeted CAR T cells for myeloma. *N. Engl. J. Med.* **387**, 1196–1206 (2022).

19. A. Papadopoulou, U. Gerdemann, U. L. Katari, I. Tzannou, H. Liu, C. Martinez, K. Leung, G. Carrum, A. P. Gee, J. F. Vera, R. A. Krance, M. K. Brenner, C. M. Rooney, H. E. Heslop, A. M. Leen, Activity of broad-spectrum T cells as treatment for AdV, EBV, CMV, BKV, and HHV6 infections after HSCT. *Sci. Transl. Med.* **6**, 242ra83 (2014).

20. I. Tzannou, A. Papadopoulou, S. Naik, K. Leung, C. A. Martinez, C. A. Ramos, G. Carrum, G. Sasa, P. Lulla, A. Watanabe, M. Kuvalekar, A. P. Gee, M. F. Wu, H. Liu, B. J. Grilley, R. A. Krance, S. Gottschalk, M. K. Brenner, C. M. Rooney, H. E. Heslop, A. M. Leen, B. Omer, Off-the-shelf virus-specific T cells to treat BK virus, human herpesvirus 6, cytomegalovirus, Epstein-Barr virus, and adenovirus infections after allogeneic hematopoietic stem-cell transplantation. *J. Clin. Oncol.* **35**, 3547–3557 (2017).

21. L. P. McLaughlin, R. Rouce, S. Gottschalk, V. Torrano, G. Carrum, M. F. Wu, F. Hoq, B. Grilley, A. M. Marcogliese, P. J. Hanley, A. P. Gee, M. K. Brenner, C. M. Rooney, H. E. Heslop, C. M. Bollard, EBV/LMP-specific T cells maintain remissions of T- and B-cell EBV lymphomas after allogeneic bone marrow transplantation. *Blood* **132**, 2351–2361 (2018).

22. S. Vasileiou, P. D. Lulla, I. Tzannou, A. Watanabe, M. Kuvalekar, W. L. Callejas, M. Bilgi, T. Wang, M. J. Wu, R. Kamble, C. A. Ramos, R. H. Rouce, Z. Zeng, A. P. Gee, B. J. Grilley, J. F. Vera, C. M. Bollard, M. K. Brenner, H. E. Heslop, C. M. Rooney, A. M. Leen, G. Carrum, T-cell therapy for lymphoma using nonengineered multiantigen-targeted T cells is safe and produces durable clinical effects. *J. Clin. Oncol.* **39**, 1415–1425 (2021).

23. S. Stevanovic, S. R. Helman, J. R. Wunderlich, M. M. Langhan, S. L. Doran, M. L. M. Kwong, R. P. T. Somerville, C. A. Klebanoff, U. S. Kammula, R. M. Sherry, J. C. Yang, S. A. Rosenberg, C. S. Hinrichs, A phase II study of tumor-infiltrating lymphocyte therapy for human papillomavirus-associated epithelial cancers. *Clin. Cancer Res.* **25**, 1486–1493 (2019).

24. L. T. Nguyen, S. D. Saibil, V. Sotov, M. X. Le, L. Khoja, D. Ghazarian, L. Bonilla, H. Majeed, D. Hogg, A. M. Joshua, M. Crump, N. Franke, A. Spreafico, A. Hansen, A. Al-Habeeb, W. Leong, A. Easson, M. Reedijk, D. P. Goldstein, D. McCready, K. Yasufuku, T. Waddell, M. Cypel, A. Pierre, B. Zhang, S. Boross-Harmer, J. Cipollone, M. Nelles, E. Scheid, M. Fyrsta, C. S. Lo, J. Nie, J. Y. Yam, P. H. Yen, D. Gray, V. Motta, A. R. Elford, S. DeLuca, L. Wang, S. Effendi, R. Ellenchery, N. Hirano, P. S. Ohashi, M. O. Butler, Phase II clinical trial of adoptive cell therapy for patients with metastatic melanoma with autologous tumor-infiltrating lymphocytes and low-dose interleukin-2. *Cancer Immunol. Immunother.* **68**, 773–785 (2019).

25. C. Saberian, R. N. Amaria, A. M. Najjar, L. G. Radvanyi, C. L. Haymaker, M. A. Forget, R. L. Bassett, S. C. Faria, I. C. Glitza, E. Alvarez, S. Parshottam, V. Prieto, G. Lizee, M. K. Wong, J. L. McQuade, A. Diab, C. Yee, H. A. Tawbi, S. Patel, E. J. Shpall, M. A. Davies, P. Hwu, C. Bernatchez, Randomized phase II trial of lymphodepletion plus adoptive cell transfer of tumor-infiltrating lymphocytes, with or without dendritic cell vaccination, in patients with metastatic melanoma. *J. Immunother. Cancer* **9**, e002449 (2021).

26. J. Chesney, K. D. Lewis, H. Kluger, O. Hamid, E. Whitman, S. Thomas, M. Wermke, M. Cusnir, E. Domingo-Musibay, G. Q. Phan, J. M. Kirkwood, J. C. Hassel, M. Orloff, J. Larkin, J. Weber, A. J. S. Furness, N. I. Khushalani, T. Medina, M. E. Egger, F. Graf Finckenstein, M. Jagasia, P. Hari, G. Sulur, W. Shi, X. Wu, A. Sarnaik, Efficacy and safety of lifileucel, a one-time autologous tumor-infiltrating lymphocyte (TIL) cell therapy, in patients with advanced melanoma after progression on immune checkpoint inhibitors and targeted therapies: Pooled analysis of consecutive cohorts of the C-144–01 study. *J. Immunother. Cancer* **10**, e005755 (2022).

27. N. Zacharakis, L. M. Huq, S. J. Seitter, S. P. Kim, J. J. Gartner, S. Sindiri, V. K. Hill, Y. F. Li, B. C. Paria, S. Ray, B. Gasmi, C. C. Lee, T. D. Prickett, M. R. Parkhurst, P. F. Robbins, M. M.

Langhan, T. E. Shelton, A. Y. Parikh, S. T. Levi, J. M. Hernandez, C. D. Hoang, R. M. Sherry, J. C. Yang, S. A. Feldman, S. L. Goff, S. A. Rosenberg, Breast cancers are immunogenic: Immunologic analyses and a phase II pilot clinical trial using mutation-reactive autologous lymphocytes. *J. Clin. Oncol.* **40**, 1741–1754 (2022).

28. N. B. Nagarsheth, S. M. Norberg, A. L. Sinkoe, S. Adhikary, T. J. Meyer, J. B. Lack, A. C. Warner, C. Schweitzer, S. L. Doran, S. Korrapati, S. Stevanovic, C. L. Trimble, J. A. Kanakry, M. H. Bagheri, E. Ferraro, S. H. Astrow, A. Bot, W. C. Faquin, D. Stroncek, N. Gkitsas, S. Highfill, C. S. Hinrichs, TCR-engineered T cells targeting E7 for patients with metastatic HPV-associated epithelial cancers. *Nat. Med.* **27**, 419–425 (2021).

29. P. F. Robbins, S. H. Kassim, T. L. Tran, J. S. Crystal, R. A. Morgan, S. A. Feldman, J. C. Yang, M. E. Dudley, J. R. Wunderlich, R. M. Sherry, U. S. Kammula, M. S. Hughes, N. P. Restifo, M. Raffeld, C. C. Lee, Y. F. Li, M. El-Gamil, S. A. Rosenberg, A pilot trial using lymphocytes genetically engineered with an NY-ESO-1-reactive T-cell receptor: Long-term follow-up and correlates with response. *Clin. Cancer Res.* **21**, 1019–1027 (2015).

30. P. F. Robbins, R. A. Morgan, S. A. Feldman, J. C. Yang, R. M. Sherry, M. E. Dudley, J. R. Wunderlich, A. V. Nahvi, L. J. Helman, C. L. Mackall, U. S. Kammula, M. S. Hughes, N. P. Restifo, M. Raffeld, C. C. Lee, C. L. Levy, Y. F. Li, M. El-Gamil, S. L. Schwarz, C. Laurencot, S. A. Rosenberg, Tumor regression in patients with metastatic synovial cell sarcoma and melanoma using genetically engineered lymphocytes reactive with NY-ESO-1. *J. Clin. Oncol.* **29**, 917–924 (2011).

31. A. G. Chapuis, D. N. Egan, M. Bar, T. M. Schmitt, M. S. McAfee, K. G. Paulson, V. Voillet, R. Gottardo, G. B. Ragnarsson, M. Bleakley, C. C. Yeung, P. Muhlhauser, H. N. Nguyen, L. A. Kropp, L. Castelli, F. Wagener, D. Hunter, M. Lindberg, K. Cohen, A. Seese, M. J. McElrath, N. Duerkopp, T. A. Gooley, P. D. Greenberg, T cell receptor gene therapy targeting WT1 prevents acute myeloid leukemia relapse post-transplant. *Nat. Med.* **25**, 1064–1072 (2019).

32. A. P. Rapoport, E. A. Stadtmauer, G. K. Binder-Scholl, O. Goloubeva, D. T. Vogl, S. F. Lacey, A. Z. Badros, A. Garfall, B. Weiss, J. Finklestein, I. Kulikovskaya, S. K. Sinha, S. Kronsberg, M. Gupta, S. Bond, L. Melchiori, J. E. Brewer, A. D. Bennett, A. B. Gerry, N. J. Pumphrey, D.

Williams, H. K. Tayton-Martin, L. Ribeiro, T. Holdich, S. Yanovich, N. Hardy, J. Yared, N. Kerr, S. Philip, S. Westphal, D. L. Siegel, B. L. Levine, B. K. Jakobsen, M. Kalos, C. H. June, NY-ESO-1-specific TCR-engineered T cells mediate sustained antigen-specific antitumor effects in myeloma. *Nat. Med.* **21**, 914–921 (2015).

33. S. A. Rosenberg, B. S. Packard, P. M. Aebersold, D. Solomon, S. L. Topalian, S. T. Toy, P. Simon, M. T. Lotze, J. C. Yang, C. A. Seipp, C. Simpson, C. Carter, S. Bock, D. Schwartzentruber, J. P. Wei, D. E. White, Use of tumor-infiltrating lymphocytes and interleukin-2 in the immunotherapy of patients with metastatic melanoma. A preliminary report. *N. Engl. J. Med.* **319**, 1676–1680 (1988).

34. V. Gudipati, J. Rydzek, I. Doel-Perez, V. D. R. Goncalves, L. Scharf, S. Konigsberger, E. Lobner, R. Kunert, H. Einsele, H. Stockinger, M. Hudecek, J. B. Huppa, Inefficient CAR-proximal signaling blunts antigen sensitivity. *Nat. Immunol.* **21**, 848–856 (2020).

35. D. T. Harris, M. V. Hager, S. N. Smith, Q. Cai, J. D. Stone, P. Kruger, M. Lever, O. Dushek, T. M. Schmitt, P. D. Greenberg, D. M. Kranz, Comparison of T cell activities mediated by human TCRs and CARs that use the same recognition domains. *J. Immunol.* **200**, 1088–1100 (2018).

36. R. Oren, M. Hod-Marco, M. Haus-Cohen, S. Thomas, D. Blat, N. Duvshani, G. Denkberg, Y. Elbaz, F. Benchetrit, Z. Eshhar, H. Stauss, Y. Reiter, Functional comparison of engineered T cells carrying a native TCR versus TCR-like antibody-based chimeric antigen receptors indicates affinity/avidity thresholds. *J. Immunol.* **193**, 5733–5743 (2014).

37. N. Anikeeva, S. Panteleev, N. W. Mazzanti, M. Terai, T. Sato, Y. Sykulev, Efficient killing of tumor cells by CAR-T cells requires greater number of engaged CARs than TCRs. *J. Biol. Chem.* **297**, 101033 (2021).

38. A. I. Salter, A. Rajan, J. J. Kennedy, R. G. Ivey, S. A. Shelby, I. Leung, M. L. Templeton, V. Muhunthan, V. Voillet, D. Sommermeyer, J. R. Whiteaker, R. Gottardo, S. L. Veatch, A. G. Paulovich, S. R. Riddell, Comparative analysis of TCR and CAR signaling informs CAR designs with superior antigen sensitivity and in vivo function. *Sci. Signal.* **14**, eabe2606 (2021).

39. N. N. Shah, T. J. Fry, Mechanisms of resistance to CAR T cell therapy. *Nat. Rev. Clin. Oncol.* **16**, 372–385 (2019).

40. R. Vita, S. Mahajan, J. A. Overton, S. K. Dhanda, S. Martini, J. R. Cantrell, D. K. Wheeler, A. Sette, B. Peters, The Immune Epitope Database (IEDB): 2018 update. *Nucleic Acids Res.* **47**, D339–D343 (2019).

41. C. Hassan, M. G. Kester, A. H. de Ru, P. Hombrink, J. W. Drijfhout, H. Nijveen, J. A. Leunissen, M. H. Heemskerk, J. H. Falkenburg, P. A. van Veelen, The human leukocyte antigen-presented ligandome of B lymphocytes. *Mol. Cell. Proteomics* **12**, 1829–1843 (2013).

42. B. Y. Jin, T. E. Campbell, L. M. Draper, S. Stevanovic, B. Weissbrich, Z. Yu, N. P. Restifo, S. A. Rosenberg, C. L. Trimble, C. S. Hinrichs, Engineered T cells targeting E7 mediate regression of human papillomavirus cancers in a murine model. *JCI Insight* **3**, e99488 (2018).

43. A. Haga-Friedman, M. Horovitz-Fried, C. J. Cohen, Incorporation of transmembrane hydrophobic mutations in the TCR enhance its surface expression and T cell functional avidity. *J. Immunol.* **188**, 5538–5546 (2012).

44. C. J. Cohen, Y. F. Li, M. El-Gamil, P. F. Robbins, S. A. Rosenberg, R. A. Morgan, Enhanced antitumor activity of T cells engineered to express T-cell receptors with a second disulfide bond. *Cancer Res.* **67**, 3898–3903 (2007).

45. K. Ishii, J. S. Davies, A. L. Sinkoe, K. A. Nguyen, S. M. Norberg, C. P. McIntosh, T. Kadakia, C. Serna, Z. Rae, M. C. Kelly, C. S. Hinrichs, Multi-tiered approach to detect autoimmune cross-reactivity of therapeutic T cell receptors. *Sci. Adv.* **9**, eadg9845 (2023).

46. A. B. Riemer, D. B. Keskin, G. Zhang, M. Handley, K. S. Anderson, V. Brusic, B. Reinhold, E. L. Reinherz, A conserved E7-derived cytotoxic T lymphocyte epitope expressed on human papillomavirus 16-transformed HLA-A2$^+$ epithelial cancers. *J. Biol. Chem.* **285**, 29608–29622 (2010).

47. B. B. Duncan, C. E. Dunbar, K. Ishii, Applying a clinical lens to animal models of CAR-T cell therapies. *Mol. Ther. Methods Clin. Dev.* **27**, 17–31 (2022).

48. H. Cheroutre, F. Lambolez, Doubting the TCR coreceptor function of CD8αα. *Immunity* **28**, 149–159 (2008).

49. M. C. Miceli, P. von Hoegen, J. R. Parnes, Adhesion versus coreceptor function of CD4 and CD8: Role of the cytoplasmic tail in coreceptor activity. *Proc. Natl. Acad. Sci. U.S.A.* **88**, 2623–2627 (1991).

50. K. C. Garcia, C. A. Scott, A. Brunmark, F. R. Carbone, P. A. Peterson, I. A. Wilson, L. Teyton, CD8 enhances formation of stable T-cell receptor/MHC class I molecule complexes. *Nature* **384**, 577–581 (1996).

51. J. Sun, P. B. Kavathas, Comparison of the roles of CD8 alpha alpha and CD8 alpha beta in interaction with MHC class I. *J. Immunol.* **159**, 6077–6082 (1997).

52. W. Haso, D. W. Lee, N. N. Shah, M. Stetler-Stevenson, C. M. Yuan, I. H. Pastan, D. S. Dimitrov, R. A. Morgan, D. J. FitzGerald, D. M. Barrett, A. S. Wayne, C. L. Mackall, R. J. Orentas, Anti-CD22-chimeric antigen receptors targeting B-cell precursor acute lymphoblastic leukemia. *Blood* **121**, 1165–1174 (2013).

53. J. Jess, B. Yates, A. Dulau-Florea, K. Parker, J. Inglefield, D. Lichtenstein, F. Schischlik, M. Ongkeko, Y. Wang, S. Shahani, A. Cullinane, H. Smith, E. Kane, L. Little, D. Chen, T. J. Fry, H. Shalabi, H. W. Wang, A. Satpathy, J. Lozier, N. N. Shah, CD22 CAR T-cell associated hematologic toxicities, endothelial activation and relationship to neurotoxicity. *J. Immunother. Cancer* **11**, e005898 (2023).

54. J. H. Baird, M. J. Frank, J. Craig, S. Patel, J. Y. Spiegel, B. Sahaf, J. S. Oak, S. F. Younes, M. G. Ozawa, E. Yang, Y. Natkunam, J. Tamaresis, Z. Ehlinger, W. D. Reynolds, S. Arai, L. Johnston, R. Lowsky, E. Meyer, R. S. Negrin, A. R. Rezvani, P. Shiraz, S. Sidana, W. K. Weng, K. L. Davis, S. Ramakrishna, L. Schultz, C. Mullins, A. Jacob, I. Kirsch, S. A. Feldman, C. L. Mackall, D. B. Miklos, L. Muffly, CD22-directed CAR T-cell therapy induces complete remissions in CD19-directed CAR-refractory large B-cell lymphoma. *Blood* **137**, 2321–2325 (2021).

55. M. Norelli, B. Camisa, G. Barbiera, L. Falcone, A. Purevdorj, M. Genua, F. Sanvito, M. Ponzoni, C. Doglioni, P. Cristofori, C. Traversari, C. Bordignon, F. Ciceri, R. Ostuni, C. Bonini, M. Casucci, A. Bondanza, Monocyte-derived IL-1 and IL-6 are differentially required for cytokine-release syndrome and neurotoxicity due to CAR T cells. *Nat. Med.* **24**, 739–748 (2018).

56. T. Giavridis, S. J. C. van der Stegen, J. Eyquem, M. Hamieh, A. Piersigilli, M. Sadelain, CAR T cell-induced cytokine release syndrome is mediated by macrophages and abated by IL-1 blockade. *Nat. Med.* **24**, 731–738 (2018).

57. I. W. Abrahamsen, E. Stronen, S. Walchli, J. N. Johansen, S. Kjellevoll, S. Kumari, M. Komada, G. Gaudernack, G. Tjonnfjord, M. Toebes, T. N. Schumacher, F. Lund-Johansen, J. Olweus, Targeting B cell leukemia with highly specific allogeneic T cells with a public recognition motif. *Leukemia* **24**, 1901–1909 (2010).

58. L. Jahn, R. S. Hagedoorn, D. M. van der Steen, P. Hombrink, M. G. Kester, M. P. Schoonakker, D. de Ridder, P. A. van Veelen, J. H. Falkenburg, M. H. Heemskerk, A CD22-reactive TCR from the T-cell allorepertoire for the treatment of acute lymphoblastic leukemia by TCR gene transfer. *Oncotarget* **7**, 71536–71547 (2016).

59. L. Jahn, P. Hombrink, R. S. Hagedoorn, M. G. Kester, D. M. van der Steen, T. Rodriguez, T. Pentcheva-Hoang, A. H. de Ru, M. P. Schoonakker, M. H. Meeuwsen, M. Griffioen, P. A. van Veelen, J. H. Falkenburg, M. H. Heemskerk, TCR-based therapy for multiple myeloma and other B-cell malignancies targeting intracellular transcription factor BOB1. *Blood* **129**, 1284–1295 (2017).

60. T. L. A. Wachsmann, A. K. Wouters, D. F. G. Remst, R. S. Hagedoorn, M. H. Meeuwsen, E. van Diest, J. Leusen, J. Kuball, J. H. F. Falkenburg, M. H. M. Heemskerk, Comparing CAR and TCR engineered T cell performance as a function of tumor cell exposure. *Onco. Targets. Ther.* **11**, 2033528 (2022).

61. T. L. A. Wachsmann, M. H. Meeuwsen, D. F. G. Remst, K. Buchner, A. K. Wouters, R. Hagedoorn, J. H. F. Falkenburg, M. H. M. Heemskerk, Combining BCMA-targeting CAR-T

with TCR-engineered T-cell therapy to prevent immune escape of multiple myeloma. *Blood Adv.* **7**, 6178–6183 (2023).

62. M. Ali, E. Giannakopoulou, Y. Li, M. Lehander, S. Virding Culleton, W. Yang, C. Knetter, M. C. Odabasi, R. C. Bollineni, X. Yang, Z. Foldvari, M. L. Boschen, E. Taraldsrud, E. Stronen, M. Toebes, A. Hillen, S. Mazzi, A. H. de Ru, G. M. C. Janssen, A. Kolstad, G. E. Tjonnfjord, B. A. Lie, M. Griffioen, S. Lehmann, L. T. Osnes, J. Buechner, K. C. Garcia, T. N. Schumacher, P. A. van Veelen, M. Leisegang, S. E. W. Jacobsen, P. Woll, J. Olweus, T cells targeted to TdT kill leukemic lymphoblasts while sparing normal lymphocytes. *Nat. Biotechnol.* **40**, 488–498 (2022).

63. M. K. O'Reilly, H. Tian, J. C. Paulson, CD22 is a recycling receptor that can shuttle cargo between the cell surface and endosomal compartments of B cells. *J. Immunol.* **186** 1554–1563(2011).

64. M. Ruella, F. Korell, P. Porazzi, M. V. Maus, Mechanisms of resistance to chimeric antigen receptor-T cells in haematological malignancies. *Nat. Rev. Drug Discov.* **22**, 976–995 (2023).

65. G. Bajwa, I. Lanz, M. Cardenas, M. K. Brenner, C. Arber, Transgenic CD8alphabeta co-receptor rescues endogenous TCR function in TCR-transgenic virus-specific T cells. *J. Immunother. Cancer* **8**, e001487 (2020).

66. J. A. Rath, G. Bajwa, B. Carreres, E. Hoyer, I. Gruber, M. A. Martinez-Paniagua, Y. R. Yu, N. Nouraee, F. Sadeghi, M. Wu, T. Wang, M. Hebeisen, N. Rufer, N. Varadarajan, P. C. Ho, M. K. Brenner, D. Gfeller, C. Arber, Single-cell transcriptomics identifies multiple pathways underlying antitumor function of TCR- and CD8alphabeta-engineered human $CD4^+$ T cells. *Sci. Adv.* **6**, eaaz7809 (2020).

67. D. Hong, J. Clarke, T. Johanns, P. Kebriaei, J. Heymach, A. Galal, S. Saibil, A. Sacher, F. Brophy, G. Betts, N. Bath, S. William, A. Tipping, J. Tucci, R. Luke, T. Trivedi, Q. Lin, J. M. Navenot, P. Fracasso, K. Miller, E. Norry, M. Dudley, M. Butler, Initial safety, efficacy, and product attributes from the SURPASS trial with ADP-A2M4CD8, a SPEAR T-cell therapy incorporating an affinity optimized TCR targeting MAGE-A4 and a CD8 αCO-RECEPTOR. *J. Immunother. Cancer* **8**, A231–A231 (2020).

68. D. S. Hong, A. Asch, E. Calvo, J. Zugazagoitia, J. Charlson, M. O. Butler, V. M. Garcia, A. Cervantes, B. A. Van Tine, D. P. Lawrence, M. L. Johnson, Q. Lin, T. Annareddy, F. Brophy, R. Broad, A. D. Soria, J. M. Navenot, J. Saro, E. Norry, J. M. Clarke, Updated safety and efficacy from SURPASS, the phase I trial of ADP-A2M4CD8, a next-generation autologous T-cell receptor T-cell therapy, in previously treated patients with unresectable or metastatic tumors. *Ann. Oncol.* **33**, S879–S880 (2022).

69. D. S. Hong, J. M. Clarke, A. Asch, J. Charlson, T. M. Johanns, E. Calvo, V. Boni, M. J. de Miguel, V. M. Garcia, D. P. Lawrence, M. O. Butler, J. Zugazagoitia, M. A. B. Murphy, P. Kebriaei, G. R. Blumenschein, Q. Lin, H. Danesi, E. Norry, Safety and efficacy from the SURPASS trial with ADP-A2M4CD8, a SPEAR T-cell therapy incorporating a CD8α co-receptor and an affinity optimized TCR targeting MAGE-A4. *Ann. Oncol.* **32**, S604–S605 (2021).

70. D. S. Hong, S. I. Jalal, E. Elimova, J. A. Ajani, M. A. B. Murphy, A. Cervantes, T. R. J. Evans, H. Park, Q. Lin, P. Noto, M. Shnaidman, D. Campbell, M. Rosenberg, F. E. Brophy, H. Danesi, D. H. Ilson, SURPASS-2 trial design: A phase 2, open-label study of ADP-A2M4CD8 SPEAR T cells in advanced esophageal or esophagogastric junction cancers. *J. Clin. Oncol.* **40**, doi.org/10.1200/JCO.2022.40.4_suppl.TPS363 (2022).

71. R. G. Dossa, T. Cunningham, D. Sommermeyer, I. Medina-Rodriguez, M. A. Biernacki, K. Foster, M. Bleakley, Development of T-cell immunotherapy for hematopoietic stem cell transplantation recipients at risk of leukemia relapse. *Blood* **131**, 108–120 (2018).

72. E. F. Krakow, M. Brault, C. Summers, T. M. Cunningham, M. A. Biernacki, R. G. Black, K. B. Woodward, N. Vartanian, S. B. Kanaan, A. C. Yeh, R. G. Dossa, M. Bar, R. D. Cassaday, A. Dahlberg, B. G. Till, A. E. Denker, C. C. S. Yeung, T. A. Gooley, D. G. Maloney, S. R. Riddell, P. D. Greenberg, A. G. Chapuis, E. W. Newell, S. N. Furlan, M. Bleakley, HA-1-targeted T-cell receptor T-cell therapy for recurrent leukemia after hematopoietic stem cell transplantation. *Blood* **144**, 1069–1082 (2024).

73. M. Shakiba, P. Zumbo, G. Espinosa-Carrasco, L. Menocal, F. Dundar, S. E. Carson, E. M. Bruno, F. J. Sanchez-Rivera, S. W. Lowe, S. Camara, R. P. Koche, V. P. Reuter, N. D. Socci, B.

Whitlock, F. Tamzalit, M. Huse, M. D. Hellmann, D. K. Wells, N. A. Defranoux, D. Betel, M. Philip, A. Schietinger, TCR signal strength defines distinct mechanisms of T cell dysfunction and cancer evasion. *J. Exp. Med.* **219**, e20201966 (2022).

74. L. Wooldridge, B. Laugel, J. Ekeruche, M. Clement, H. A. van den Berg, D. A. Price, A. K. Sewell, CD8 controls T cell cross-reactivity. *J. Immunol.* **185**, 4625–4632 (2010).

75. L. Wooldridge, M. Clement, A. Lissina, E. S. Edwards, K. Ladell, J. Ekeruche, R. E. Hewitt, B. Laugel, E. Gostick, D. K. Cole, R. Debets, C. Berrevoets, J. J. Miles, S. R. Burrows, D. A. Price, A. K. Sewell, MHC class I molecules with superenhanced CD8 binding properties bypass the requirement for cognate TCR recognition and nonspecifically activate CTLs. *J. Immunol.* **184**, 3357–3366 (2010).

76. M. Clement, L. Knezevic, T. Dockree, J. E. McLaren, K. Ladell, K. L. Miners, S. Llewellyn-Lacey, A. Rubina, O. Francis, D. K. Cole, A. K. Sewell, J. S. Bridgeman, D. A. Price, H. A. van den Berg, L. Wooldridge, CD8 coreceptor-mediated focusing can reorder the agonist hierarchy of peptide ligands recognized via the T cell receptor. *Proc. Natl. Acad. Sci. U.S.A.* **118**, e2019639118 (2021).

77. L. Knezevic, T. L. A. Wachsmann, O. Francis, T. Dockree, J. S. Bridgeman, A. Wouters, B. de Wet, D. K. Cole, M. Clement, J. E. McLaren, E. Gostick, K. Ladell, S. Llewellyn-Lacey, D. A. Price, H. A. van den Berg, Z. Tabi, R. B. Sessions, M. H. M. Heemskerk, L. Wooldridge, High-affinity CD8 variants enhance the sensitivity of pMHCI antigen recognition via low-affinity TCRs. *J. Biol. Chem.* **299**, 104981 (2023).

78. H. A. van den Berg, L. Wooldridge, B. Laugel, A. K. Sewell, Coreceptor CD8-driven modulation of T cell antigen receptor specificity. *J. Theor. Biol.* **249**, 395–408 (2007).

79. J. Feucht, J. Sun, J. Eyquem, Y. J. Ho, Z. Zhao, J. Leibold, A. Dobrin, A. Cabriolu, M. Hamieh, M. Sadelain, Calibration of CAR activation potential directs alternative T cell fates and therapeutic potency. *Nat. Med.* **25**, 82–88 (2019).

80. S. Ramakrishna, S. L. Highfill, Z. Walsh, S. M. Nguyen, H. Lei, J. F. Shern, H. Qin, I. L. Kraft, M. Stetler-Stevenson, C. M. Yuan, J. D. Hwang, Y. Feng, Z. Zhu, D. Dimitrov, N. N. Shah, T. J. Fry, Modulation of target antigen density improves CAR T-cell functionality and persistence. *Clin. Cancer Res.* **25**, 5329–5341 (2019).

81. M. Boulch, M. Cazaux, A. Cuffel, M. Ruggiu, V. Allain, B. Corre, Y. Loe-Mie, B. Hosten, S. Cisternino, S. Auvity, C. Thieblemont, S. Caillat-Zucman, P. Bousso, A major role for $CD4^+$ T cells in driving cytokine release syndrome during CAR T cell therapy. *Cell Rep. Med.* **4**, 101161 (2023).

82. C. Bove, S. Arcangeli, L. Falcone, B. Camisa, R. El Khoury, B. Greco, A. De Lucia, A. Bergamini, A. Bondanza, F. Ciceri, C. Bonini, M. Casucci, CD4 CAR-T cells targeting CD19 play a key role in exacerbating cytokine release syndrome, while maintaining long-term responses. *J. Immunother. Cancer* **11**, e005878 (2023).

83. D. Alizadeh, D. Wang, C. E. Brown, Uncovering the role of CD4+ CAR T cells in cancer immunotherapy. *Cancer Res.* **83**, 2813–2815 (2023).

84. J. Borst, T. Ahrends, N. Babala, C. J. M. Melief, W. Kastenmuller, $CD4^+$ T cell help in cancer immunology and immunotherapy. *Nat. Rev. Immunol.* **18**, 635–647 (2018).

85. M. E. Kohler, T. J. Fry, $CD4^+$ CAR T cells—More than helpers. *Nat. Cancer* **4**, 928–929 (2023).

86. D. Y. Oh, L. Fong, Cytotoxic $CD4^+$ T cells in cancer: Expanding the immune effector toolbox. *Immunity* **54**, 2701–2711 (2021).

87. D. E. Speiser, O. Chijioke, K. Schaeuble, C. Munz, $CD4^+$ T cells in cancer. *Nat. Cancer* **4**, 317–329 (2023).

88. D. Sommermeyer, M. Hudecek, P. L. Kosasih, T. Gogishvili, D. G. Maloney, C. J. Turtle, S. R. Riddell, Chimeric antigen receptor-modified T cells derived from defined $CD8^+$ and $CD4^+$ subsets confer superior antitumor reactivity in vivo. *Leukemia* **30**, 492–500 (2016).

89. A. Schietinger, M. Philip, R.B. Liu, K. Schreiber, H. Schreiber, Bystander killing of cancer requires the cooperation of $CD4^+$ and $CD8^+$ T cells during the effector phase. *J. Exp. Med.* **207**, 2469–2477 (2010).

90. D. Wang, B. Aguilar, R. Starr, D. Alizadeh, A. Brito, A. Sarkissian, J. R. Ostberg, S. J. Forman, C. E. Brown, Glioblastoma-targeted $CD4^+$ CAR T cells mediate superior antitumor activity. *JCI Insight* **3**, e99048 (2018).

91. Y. Yang, M. E. Kohler, C. D. Chien, C. T. Sauter, E. Jacoby, C. Yan, Y. Hu, K. Wanhainen, H. Qin, T. J. Fry, TCR engagement negatively affects CD8 but not CD4 CAR T cell expansion and leukemic clearance. *Sci. Transl. Med.* **9**, eaag1209, (2017).

92. M. Boulch, M. Cazaux, Y. Loe-Mie, R. Thibaut, B. Corre, F. Lemaitre, C. L. Grandjean, Z. Garcia, P. Bousso, A cross-talk between CAR T cell subsets and the tumor microenvironment is essential for sustained cytotoxic activity. *Sci. Immunol.* **6**, eabd4344, (2021).

93. M. Boulch, M. Cazaux, A. Cuffel, M. V. Guerin, Z. Garcia, R. Alonso, F. Lemaitre, A. Beer, B. Corre, L. Menger, C. L. Grandjean, F. Morin, C. Thieblemont, S. Caillat-Zucman, P. Bousso, Tumor-intrinsic sensitivity to the pro-apoptotic effects of IFN-gamma is a major determinant of $CD4^+$ CAR T-cell antitumor activity. *Nat. Cancer* **4**, 968–983 (2023).

94. K. Ishii, M. Pouzolles, C. D. Chien, R. A. Erwin-Cohen, M. E. Kohler, H. Qin, H. Lei, S. Kuhn, A. K. Ombrello, A. Dulau-Florea, M. A. Eckhaus, H. Shalabi, B. Yates, D. A. Lichtenstein, V. S. Zimmermann, T. Kondo, J. F. Shern, H. A. Young, N. Taylor, N. N. Shah, T. J. Fry, Perforin-deficient CAR T cells recapitulate late-onset inflammatory toxicities observed in patients. *J. Clin. Invest.* **130**, 5425–5443 (2020).

95. M. B. Leick, S. Han, K. M. E. Gallagher, H. Silva, G. Martin, S. Camp, H. Y. Chu, R. Gillani, M. C. Kann, B. D. Choi, R. Larson, M. Phillips, T. Kienka, S. R. Bailey, C. Graham, C. R. Reilly, M. Jan, E. Gonzalez, N. K. Horick, J. Budka, S. Filosto, R. R. Shen, E. Van Allen, S. H. AlDubayan, M. V. Maus, Patients with deleterious germline variants in STXBP2 develop toxicity after CAR-T cell therapy with axicabtagene ciloleucel. *Blood* **140**, 646–647 (2022).

96. S. E. Stepp, R. Dufourcq-Lagelouse, F. Le Deist, S. Bhawan, S. Certain, P. A. Mathew, J. I. Henter, M. Bennett, A. Fischer, G. de Saint Basile, V. Kumar, Perforin gene defects in familial hemophagocytic lymphohistiocytosis. *Science* **286**, 1957–1959(1999).

97. M. R. Jenkins, A. Tsun, J. C. Stinchcombe, G. M. Griffiths, The strength of T cell receptor signal controls the polarization of cytotoxic machinery to the immunological synapse. *Immunity* **31**, 621–631 (2009).

98. M. E. Kohler, Y. M. Yang, G. Sepulveda, T. J. Fry, A direct comparison of the in vivo efficacy and in vitro signaling of chimeric antigen receptors and endogenous T cell receptors. *Blood* **130**, 4451 (2017).

99. J. Mansilla-Soto, J. Eyquem, S. Haubner, M. Hamieh, J. Feucht, N. Paillon, A. E. Zucchetti, Z. Li, M. Sjostrand, P. L. Lindenbergh, M. Saetersmoen, A. Dobrin, M. Maurin, A. Iyer, A. Garcia Angus, M. M. Miele, Z. Zhao, T. Giavridis, S. J. C. van der Stegen, F. Tamzalit, I. Riviere, M. Huse, R. C. Hendrickson, C. Hivroz, M. Sadelain, HLA-independent T cell receptors for targeting tumors with low antigen density. *Nat. Med.* **28**, 345–352 (2022).

100. A. M. Tousley, M. C. Rotiroti, L. Labanieh, L. W. Rysavy, W. J. Kim, C. Lareau, E. Sotillo, E. W. Weber, S. P. Rietberg, G. N. Dalton, Y. Yin, D. Klysz, P. Xu, E. L. de la Serna, A. R. Dunn, A. T. Satpathy, C. L. Mackall, R. G. Majzner, Co-opting signalling molecules enables logic-gated control of CAR T cells. *Nature* **615**, 507–516 (2023).

101. M. E. Hoekstra, L. Bornes, F. E. Dijkgraaf, D. Philips, I. N. Pardieck, M. Toebes, D. S. Thommen, J. van Rheenen, T. N. M. Schumacher, Long-distance modulation of bystander tumor cells by $CD8^+$ T cell-secreted IFNgamma. *Nat. Cancer* **1**, 291–301 (2020).

102. V. Hoyos, B. Savoldo, C. Quintarelli, A. Mahendravada, M. Zhang, J. Vera, H. E. Heslop, C. M. Rooney, M. K. Brenner, G. Dotti, Engineering CD19-specific T lymphocytes with interleukin-15 and a suicide gene to enhance their anti-lymphoma/leukemia effects and safety. *Leukemia* **24**, 1160–1170 (2010).

103. M. Chmielewski, C. Kopecky, A. A. Hombach, H. Abken, IL-12 release by engineered T cells expressing chimeric antigen receptors can effectively muster an antigen-independent macrophage response on tumor cells that have shut down tumor antigen expression. *Cancer Res.* **71**, 5697–5706 (2011).

104. H. J. Pegram, J. C. Lee, E. G. Hayman, G. H. Imperato, T. F. Tedder, M. Sadelain, R. J. Brentjens, Tumor-targeted T cells modified to secrete IL-12 eradicate systemic tumors without need for prior conditioning. *Blood* **119**, 4133–4141 (2012).

105. M. Chmielewski, H. Abken, CAR T cells releasing IL-18 convert to T-Bet(high) FoxO1(low) effectors that exhibit augmented activity against advanced solid tumors. *Cell Rep.* **21**, 3205–3219 (2017).

106. K. Adachi, Y. Kano, T. Nagai, N. Okuyama, Y. Sakoda, K. Tamada, IL-7 and CCL19 expression in CAR-T cells improves immune cell infiltration and CAR-T cell survival in the tumor. *Nat. Biotechnol.* **36**, 346–351 (2018).

107. L. A. Johnson, B. Heemskerk, D. J. Powell Jr., C. J. Cohen, R. A. Morgan, M. E. Dudley, P. F. Robbins, S. A. Rosenberg, Gene transfer of tumor-reactive TCR confers both high avidity and tumor reactivity to nonreactive peripheral blood mononuclear cells and tumor-infiltrating lymphocytes. *J. Immunol.* **177**, 6548–6559 (2006).

108. S. Stevanovic, A. Pasetto, S. R. Helman, J. J. Gartner, T. D. Prickett, B. Howie, H. S. Robins, P. F. Robbins, C. A. Klebanoff, S. A. Rosenberg, C. S. Hinrichs, Landscape of immunogenic tumor antigens in successful immunotherapy of virally induced epithelial cancer. *Science* **356**, 200–205 (2017).

109. B. Rodenko, M. Toebes, S. R. Hadrup, W. J. van Esch, A. M. Molenaar, T. N. Schumacher, H. Ovaa, Generation of peptide-MHC class I complexes through UV-mediated ligand exchange. *Nat. Protoc.* **1**, 1120–1132 (2006).

110. Z. Zheng, N. Chinnasamy, R. A. Morgan, Protein L: A novel reagent for the detection of chimeric antigen receptor (CAR) expression by flow cytometry. *J. Transl. Med.* **10**, 29 (2012).

111. I. F. Hermans, J. D. Silk, J. Yang, M. J. Palmowski, U. Gileadi, C. McCarthy, M. Salio, F. Ronchese, V. Cerundolo, The VITAL assay: A versatile fluorometric technique for assessing CTL- and NKT-mediated cytotoxicity against multiple targets in vitro and in vivo. *J. Immunol. Methods* **285**, 25–40 (2004).

112. G. G. Kim, V. S. Donnenberg, A. D. Donnenberg, W. Gooding, T. L. Whiteside, A novel multiparametric flow cytometry-based cytotoxicity assay simultaneously immunophenotypes effector cells: Comparisons to a 4 h 51Cr-release assay. *J. Immunol. Methods* **325**, 51–66 (2007).

113. J. Stanke, C. Hoffmann, U. Erben, H. von Keyserling, S. Stevanovic, G. Cichon, A. Schneider, A. M. Kaufmann, A flow cytometry-based assay to assess minute frequencies of $CD8^+$ T cells by their cytolytic function. *J. Immunol. Methods* **360**, 56–65 (2010).

114. R. H. Vonderheide, W. C. Hahn, J. L. Schultze, L. M. Nadler, The telomerase catalytic subunit is a widely expressed tumor-associated antigen recognized by cytotoxic T lymphocytes. *Immunity* **10**, 673–679 (1999).

115. Y. Kim, J. Ponomarenko, Z. Zhu, D. Tamang, P. Wang, J. Greenbaum, C. Lundegaard, A. Sette, O. Lund, P. E. Bourne, M. Nielsen, B. Peters, Immune epitope database analysis resource. *Nucleic Acids Res.* **40**, W525–W530 (2012).

116. M. Nielsen, C. Lundegaard, P. Worning, S. L. Lauemoller, K. Lamberth, S. Buus, S. Brunak, O. Lund, Reliable prediction of T-cell epitopes using neural networks with novel sequence representations. *Protein Sci.* **12**, 1007–1017 (2003).

117. C. Lundegaard, K. Lamberth, M. Harndahl, S. Buus, O. Lund, M. Nielsen, NetMHC-3.0: Accurate web accessible predictions of human, mouse and monkey MHC class I affinities for peptides of length 8–11. *Nucleic Acids Res.* **36**, W509 –W512 (2008).

118. M. Andreatta, M. Nielsen, Gapped sequence alignment using artificial neural networks: Application to the MHC class I system. *Bioinformatics* **32**, 511–517 (2016).

119. B. Peters, A. Sette, Generating quantitative models describing the sequence specificity of biological processes with the stabilized matrix method. *BMC Bioinformatics* **6**, 132 (2005).

120. J. Sidney, E. Assarsson, C. Moore, S. Ngo, C. Pinilla, A. Sette, B. Peters, Quantitative peptide binding motifs for 19 human and mouse MHC class I molecules derived using positional scanning combinatorial peptide libraries. *Immunome Res.* **4**, 2 (2008).

121. E. de Castro, C. J. Sigrist, A. Gattiker, V. Bulliard, P. S. Langendijk-Genevaux, E. Gasteiger, A. Bairoch, N. Hulo, ScanProsite: Detection of PROSITE signature matches and ProRule-associated functional and structural residues in proteins. *Nucleic Acids Res.* **34**, W362–W365 (2006).

122. D. Bordo, P. Argos, Suggestions for "safe" residue substitutions in site-directed mutagenesis. *J. Mol. Biol.* **217**, 721–729 (1991).

123. S. Henikoff, J. G. Henikoff, Amino acid substitution matrices from protein blocks. *Proc. Natl. Acad. Sci. U.S.A.* **89**, 10915–10919 (1992).

124. C. Pommie, S. Levadoux, R. Sabatier, G. Lefranc, M. P. Lefranc, IMGT standardized criteria for statistical analysis of immunoglobulin V-REGION amino acid properties. *J. Mol. Recognit.* **17**, 17–32 (2004).

125. S. F. Altschul, W. Gish, W. Miller, E. W. Myers, D. J. Lipman, Basic local alignment search tool. *J. Mol. Biol.* **215**, 403–410 (1990).

126. G. Ehx, J. Somja, H. J. Warnatz, C. Ritacco, M. Hannon, L. Delens, G. Fransolet, P. Delvenne, J. Muller, Y. Beguin, H. Lehrach, L. Belle, S. Humblet-Baron, F. Baron, Xenogeneic graft-versus-host disease in humanized NSG and NSG-HLA-A2/HHD mice. *Front. Immunol.* **9**, 1943 (2018).

127. A. Ochi, R. G. Hawley, T. Hawley, M. J. Shulman, A. Traunecker, G. Kohler, N. Hozumi, Functional immunoglobulin M production after transfection of cloned immunoglobulin heavy and light chain genes into lymphoid cells. *Proc. Natl. Acad. Sci. U.S.A.* **80**, 6351–6355 (1983).

128. N. Ali, B. Flutter, R. Sanchez Rodriguez, E. Sharif-Paghaleh, L. D. Barber, G. Lombardi, F. O. Nestle, Xenogeneic graft-versus-host-disease in NOD-scid IL-2Rgammanull mice display a T-effector memory phenotype. *PLOS ONE* **7**, e44219 (2012).
